# Extending fluorescence anisotropy to large complexes using reversibly switchable proteins

**DOI:** 10.1038/s41587-022-01489-7

**Published:** 2022-10-10

**Authors:** Andrea Volpato, Dirk Ollech, Jonatan Alvelid, Martina Damenti, Barbara Müller, Andrew G York, Maria Ingaramo, Ilaria Testa

**Affiliations:** 1 Department of Applied Physics and Science for Life Laboratory, KTH Royal Institute of Technology, Stockholm, Sweden; 2 Department of Infectious Diseases, Virology, Centre for Integrative Infectious Disease Research, University Hospital Heidelberg, Heidelberg, Germany; 3 Calico Life Sciences LLC, South San Francisco, CA USA

**Keywords:** Biological fluorescence, Nanoscale biophysics

## Abstract

The formation of macromolecular complexes can be measured by detection of changes in rotational mobility using time-resolved fluorescence anisotropy. However, this method is limited to relatively small molecules (~0.1–30 kDa), excluding the majority of the human proteome and its complexes. We describe selective time-resolved anisotropy with reversibly switchable states (STARSS), which overcomes this limitation and extends the observable mass range by more than three orders of magnitude. STARSS is based on long-lived reversible molecular transitions of switchable fluorescent proteins to resolve the relatively slow rotational diffusivity of large complexes. We used STARSS to probe the rotational mobility of several molecular complexes in cells, including chromatin, the retroviral Gag lattice and activity-regulated cytoskeleton-associated protein oligomers. Because STARSS can probe arbitrarily large structures, it is generally applicable to the entire human proteome.

## Abstract (web summary)

STARSS measures rotational dynamics of large molecules and their complexes.

## Main

In the past two decades, reversible photoswitching between ON (fluorescent) and OFF (dark) molecular states in fluorescent molecules has enabled techniques operating at high spatial resolution, such as stimulated emission depletion microscopy (STED)^1^, reversible saturable/switchable optical fluorescence transitions (RESOLFT)^2^ and ground-state depletion microscopy^3^, as well as new sensitive Förster resonance energy transfer (FRET) approaches^4,5^.

Here we take advantage of the same photoswitchable states, not to improve the spatial resolution of light microscopy but rather to increase the observable time window of dipole orientation in rotational diffusion measurements by several orders of magnitude.

Rotational diffusion provides direct information on the size and local environment of molecular complexes in solution and cells. It is usually measured via fluorescence anisotropy (FA) in steady-state and time-resolved (TR-FA) modes^6^. TR-FA probes changes in molecular orientation over a time window defined by fluorescence lifetime, which is 1–5 ns for frequently used fluorophores. FA is widely used within life sciences for drug screening applications and other small molecule binding assays^7^ due to its molecular specificity and sensitivity, high throughput and compatibility with microscopy^8–10^. However, conventional TR-FA cannot reveal mass and its changes upon binding of most of the human proteome because the molecular complexes are too large (>30 kDa)—that is, they tumble too slowly to be distinguished from stationary within the nanosecond-scale time window of conventional TR-FA. The use of phosphorescence, or delayed fluorescence emission, rather than conventional fluorescence^11^ can in principle extend the time domain of TR-FA but at the cost of long acquisition times, poor signal and high light doses.

**Fig. 1:**
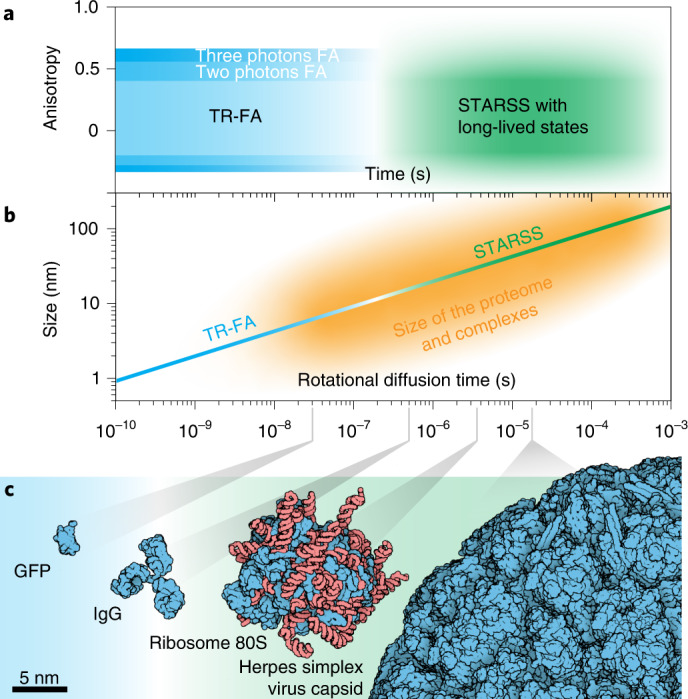
STARSS. **a**, Anisotropy values and temporal regime accessible to conventional time-resolved anisotropy and STARSS. By continuously probing the same subset of molecules populating the same reversible switchable state, it is possible to extend the observable time window to microseconds and milliseconds. This previously inaccessible temporal regime enables measurement of slower rotational diffusion. **b**, STARSS can measure slower rotational diffusion coefficients and thereby quantify the mass and mass changes of large protein complexes, as well as complex dynamics, in a local viscous environment. The relationship between rotational diffusion time and the size of complexes is shown, together with the domain of applicability of techniques. **c**, Size of proteins and biological complexes that can be investigated with STARSS.

Here we propose STARSS (Fig. 1), an approach that overcomes these limitations in anisotropy measurements using long-lived (microseconds to minutes) ON–OFF reversible molecular transitions. When induced by polarized light, molecular transitions such as *cis–trans* isomerization enable extension of the time window used to probe molecular relaxation by several orders of magnitude compared with the lifetime of fluorescence emission as in conventional TR-FA (Fig. 1). This is pivotal to measurement of the rotational dynamics of larger molecules and their complexes in aqueous or viscous solution. Because STARSS is compatible with live cell imaging and protein tagging, it can be applied to study changes in mass, local viscosity or degree of rotational freedom of most proteins in their native environment. As a proof of principle, we use STARSS to probe chromatin flexibility, viral maturation states and activity-regulated cytoskeleton-associated protein (Arc) oligomerization in highly concentrated subcellular compartments.

## STARSS with long-lived molecular states: breaking the mass limit

Reversibly photoswitchable fluorescent proteins (rsFPs), such as rsEGFP^12^ or Dronpa^13^, can be cycled between the ON and OFF states many times via ultraviolet and cyan light, and these states can persist for several minutes. If photoswitching is induced with polarized light, a subset of oriented molecules can be selected and their orientation probed for orders-of-magnitude longer time than in conventional TR-FA. This is possible only thanks to the long-lived and light-tunable lifetimes of the ON and OFF states (microseconds to minutes) as compared with the faster fluorescence lifetime (ns). Due to the extended temporal window, relatively slow rotational diffusivity—which was previously not accessible with conventional TR-FA—can now be measured. In turn, the mass of most proteins, either large or in a viscous local environment, can be addressed with STARSS when rigidly tagged with rsFPs.

**Fig. 2:**
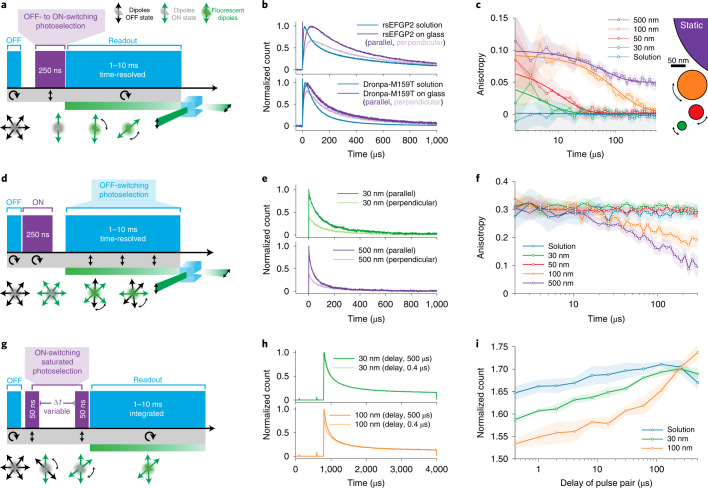
STARSS with long-lived states in reversibly photoswitchable fluorescent proteins. **a**, STARSS method 1 pulse scheme with photoselection during ON-switching, circular cyan light for probing and polarization-sensitive, two-channel detection. This methodology is the closest to typical TR-FA experiments. **b**, Experimental raw data recorded with STARSS method 1, showing distinct relaxation of the parallel and perpendicular channels for DronpaM159T and rsEGFP2 after ON-switching photoselection. **c**, STARSS method 1 experiments on beads of varying diameter. The logarithmic *x* axis shows the extended temporal observation window up to 500 µs, which allows measurement of tumbling of spheres of diameter 30–100 nm (500-nm beads are a reference static sample). Monoexponential fittings are shown, with parameters reported in Supplementary Information 3.2. **d**, STARSS method 2 pulse scheme with photoselection during OFF-switching, and with circular ON-switching and polarized-sensitive detection. **e**, Detected raw signal recorded with STARSS method 2 for beads of varying size, reporting the decay of parallel and perpendicular channels. **f**, STARSS method 2 experiments on beads of varying diameter. **g**, STARSS method 3 consists of photoselection with polarized ON-switching pulses delivered at two distinct time points and circular cyan light to read out the fluorescence detected with polarized-sensitive detection. **h**, Detected raw signal with STARSS method 3 under two delay conditions for beads of varying size. **i**, STARSS method 3 curve derived from beads of varying size. The information on rotational diffusion is encoded in the increase in count for different delays between ON-switching pulses. Counts are normalized by the signal obtained from a scheme with a single ON-switching pulse. Shaded regions of anisotropy values are 95% confidence intervals evaluated from detector noise (Supplementary Information 20).

Because the ON and OFF states are both long lived, photoselection can be applied in both directions allowing diverse implementations of the concept (Fig. 2). First we tested photoselection from the OFF–ON transition by applying a short pulse of ON-switching light (250 ns at 405 nm) followed by a long probing pulse of cyan light (1–10 ms at 488 nm; STARSS method 1, Fig. 2a). The fluorescence signal is mainly generated by the photoselected molecules, which are uniquely populating the ON states while the majority or randomly oriented molecules remain in the OFF and nondetectable state. The population of rsFPs in the ON state can be excited several times, and their fluorescence photons collected in p- and s-polarized detection channels.

The orientation of the transition dipole moments of rsFPs during photoselection and emission needs to be aligned to avoid anisotropy loss. Additionally, rsFPs should be photoselected more rapidly than rotational diffusivity such that sufficent numbers of photons can be recorded before randomization of orientation. Based on these prerequisites, we identified the fast-switching fluorophores rsEGFP2 (ref. ^12^) and DronpaM159T^14^ as suitable. Both proteins showed a long-lived anisotropy signal when adhered to a glass surface, proving that OFF–ON chromophore restructuring largely preserves orientational information (Fig. 2b). Next, we demonstrated that our method can measure rotational diffusion dynamics in the micro-to-millisecond temporal regime (Fig. 2c), extending the mass limit of TR-FA from a few kDa to the MDa range. We measured the tumbling dynamics of rsEGFP2-conjugated silica spheres in aqueous buffer for different sphere diameters. The anisotropy curves of spheres of diameter 30–100 nm (~15–60 MDa) resulted in good separation and distance from larger (500-nm) static beads. It was also possible to detect fine size differences of **~**10 nm (Supplementary Information 1). Because this probing scheme is nearly equivalent to conventional TR-FA, we can quantify our curves by direct extension of the traditional theory to a longer time window (Supplementary Information 2 and 3). Bead size was estimated using a monoexponential fit (Fig. 2c), and the rotational diffusion of spherical objects was extrapolated by the Stokes–Einstein relationship^6^. As a result, the measured and expected sizes of beads were in excellent agreement (Supplementary Information 4).

The rate of OFF-switching of rsEGFP and Dronpa variants can be tuned from microseconds to minutes by increasing or reducing the power density of 488-nm illumination. Due to this flexibility, the temporal probing window in STARSS can be adapted to the rotational diffusion range of interest. At minimal OFF-switching rate, STARSS is capable of measuring slow rotational diffusion and therefore distinguishing masses, even those of the largest biomolecular complexes. Instead, our ability to measure arbitrarily rapid diffusion with this approach is limited by ON-switching kinetics (Supplementary Information 5).

To bypass the limitations imposed by the ON-switching process, we developed a second approach where both photoselection and probing are done via the ON–OFF transition (STARSS method 2; Fig. 2d). At early time points in the process (1–10 μs), before much OFF-switching can occur, this approach yields the standard steady-state FA, which is sensitive to very rapid rotational diffusion occurring during fluorescence lifetime, as well as to other processes like homo-FRET^5^. At later time points, the polarized light responsible for excitation and OFF-switching will selectively silence any fluorophores similarly aligned. Thus, remaining long-lived fluorescence signals can be generated only by the slow tumbling of molecules with orthogonal orientation at the start.

This approach yields more photons, because all molecules can be switched ON at the beginning of the pulse scheme without temporal limitation, which translates to roughly tenfold higher photon yield for each cycle of the pulse scheme (100% of molecules switched ON compared with ~10% for method 1). Moreover, because photoselection and readout are performed simultaneously on the very same state, orientational misalignment induced by the *cis–tran*s conformational change of the fluorophore will not affect measurements.

In this approach, competition between OFF-switching kinetics and rotational diffusion governs anisotropy curves. If OFF-switching is the more rapid process, this will result in pronounced anisotropy decay as molecules aligned with the electric field are efficiently silenced, decreasing counts in the parallel channel. In contrast, if rotational diffusion is more rapid there will be rapid reshuffling of the orientation of the fluorophores and anisotropy will be stationary.

During measurement of rsEGFP2-labeled beads in this modality, we observed more pronounced decay curves for beads of 100 and 500 nm, where OFF-switching outcompetes rotational diffusion. The resulting curves differ in shape compared with the first approach and require interpretation of a different theory, which deviates from the conventional TR-FA model (Supplementary Information 6). We developed an eight-state analytical model built on photoswitching and diffusion to interpret and quantify our experimental curves (Supplementary Information 7).

Like method 1, this method also benefits from the light-driven tunability of the OFF-switching rate and it is readily adapted to the desired regime of rotational diffusion with no theoretically defined upper boundary. Instead, the lower bound is limited by the most rapidly achievable OFF-switching kinetics, which cannot be pushed to arbitrarily rapid rates. At high power densities (>15 kW cm^–2^) the OFF-switching curves of rsEGFP2 become multiexponential with a slow component of ~50 µs (Supplementary Information 8). Under these conditions, light-induced, excited-state processes are no longer the rate-determining step: instead, ground-state intermediate processes, such as protonation of the chromophore, limit the speed of the OFF-switching process.

To measure even more rapid tumbling rates, and to close the temporal gap between conventional TR-FA and STARSS, we developed a third STARSS approach based on ON-switching photoselection (Fig. 2g–i). Two linearly polarized **~**50-ns short pulses at 405 nm, separated by a variable time delay of 0.2–500 µs. are used to photoselect and drive rsFPs into the ON state. The fluorescence signal is then probed with cyan light (STARSS method 3; Fig. 2g). The modulation of these two signals is determined by the rotational diffusion that occurs during the delay between pump pulses (Fig. 2h and Supplementary Information 9). Following the trend of the ON-switching curves (Supplementary Information 10), the first pulse should switch ON roughly 50% of molecules for optimal modulation. The second pulse attains a saturation effect because a substantial number of the molecules are already populating the ON state. We also validated this method using beads of known size (Fig. 2i). In this case, the curves increased at longer delay times for 100-nm beads, whose diffusion is slower. Because the kinetics of ON-switching are much more rapid than OFF-switching in rsEGFP2^15,16^ and Dronpa^14^, this method is the most sensitive to fast rotational diffusion. We observed rotational diffusivity of molecules as small as rsEGFP2 (∼16.5 ns of EGFP)^17^.

Like the second method, this third pulse scheme is not affected by anisotropy loss caused by OFF–ON state transition because photoselection is performed on the same molecular OFF state by the pulse pair. The hardware complexity in the third approach is further reduced by the absence of polarization-sensitive or time-resolved detection, providing future avenues for potential simplification of STARSS.

Taken together, these three approaches enable us to distinguish molecular weights from those comparable to a GFP of 27 kDa (5-nm hydrodynamic diameter) and all the way up to 60 MDa (100-nm hydrodynamic diameter), covering the size range of nearly the entire ensemble of molecules and protein complexes in eukaryotic cells.

## STARSS with rigidly linked rsEGFP2 reveals flexibility of membrane proteins and nucleosomes in living cells

In STARSS experiments it is pivotal to minimize the local motion of the probe to ensure a representative measure of the global rotational dynamics. We validated different strategies to increase the rigidity between tag and target protein to measure the rotational motion of large molecular complexes in living cells.

**Fig. 3:**
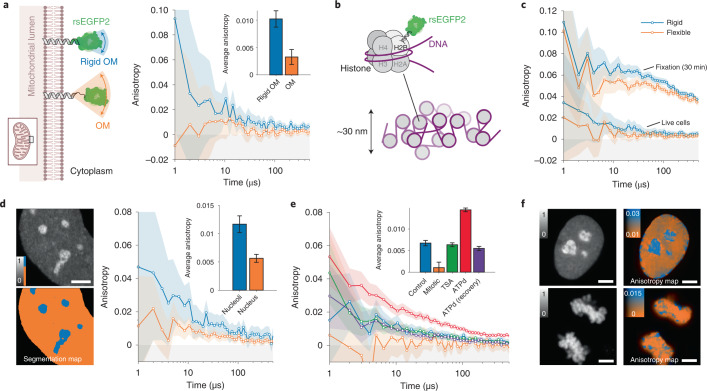
Probing biomolecular structures in living cells with STARSS. **a**, Live human U2OS cells expressing rsEGFP2 targeted to the mitochondrial outer membrane (OM) with different linker sizes were measured using STARSS method 1. A short rigid helical linker (blue, integrated over *n* = 9 cells) resulted in higher anisotropy values compared with a longer flexible linker (orange, integrated over *n* = 7 cells). **b**, Cartoon of histone H2B fused with rsEGFP2 via a prolonged terminal alpha-helix (upper left) as a unit of chromatin filaments of size of ~30 nm (bottom). **c**, Anisotropy recorded in the rigid and flexible constructs of H2B-rsEGFP2 in U2OS cells with STARSS method 1, in live cells and fixed cells (integrated over *n* = 3 cells). **d**, Representative fluorescence intensity image and segmentation map of nucleoli/nucleus (left, scale bar 4 μm) used to split the anisotropy measured with STARSS method 1 in blue and orange datasets (right, integrated over *n* = 8 cells). **e**, Anisotropy decays recorded with STARSS method 1 in HeLa cells expressing rigid H2B-rsEGFP2 for interphase chromatin (integrated over *n* = 8 cells), mitotic chromosomes (integrated over *n* = 18 cells), treatment of interphase chromatin with TSA (integrated over *n* = 8 cells) and treatment and recovery of interphase chromatin for ATP depletion (ATPd, both integrated over *n* = 8 cells). **f**, Fluorescence count maps (left) and fluorescence anisotropy maps (right) for two representative fields of view of chromatin in an interphase nucleus after ATPd (top), and mitotic chromosomes (bottom). Scale bars, 4 μm. In all relevant panels, error bars and shaded regions of anisotropy values are 95% confidence intervals evaluated from detector noise according to Supplementary Information 20.

First, we reduced the size of the peptide linker that connects rsEGFP2 to the target structure (Fig. 3a). We compared a flexible mitochondrial outer-membrane-localized protein, rsEGFP2-OMP25 (ref. ^18^), with a purposely designed rigid TOM20-rsEGFP2 using STARSS method 1. The flexible rsEGFP2 C terminus was linked to the 30-amino-acid-long N-terminal loop structure of OMP25 (10.5 nm in stretched conformation (Supplementary Information 11.1)), providing full rotational freedom of the rsFP-tag. To increase rigidity we connected the translocation domain of TOM20, which contains only five unstructured membrane proximal residues, directly to an N-terminally truncated rsEGFP2 via the short and rigid helix-forming linker EAAAKA^19^ (estimated linker length 2 nm (Supplementary Information 11.2)). The short linker reduces the rotational freedom of the probe, resulting in a threefold increase in anisotropy values compared with the flexible tag. This demonstrates the applicability of STARSS to probing the intracellular rotational freedom of membrane-targeted proteins.

Second, we applied STARSS method 1 to the study of nonmembrane-bound proteins. For proteins comprising a terminal alpha-helix, it is possible to generate a rigid fusion variant by blending it into the N-terminal helix of GFP-related FPs^19,20^. We applied this strategy by prolonging the C-terminal helix of histone protein H2B with the EAAAKA linker fused to the truncated rsEGFP2 (Fig. 3b and Supplementary Information 11.3). Built by two copies of each core histone, H2A, H2B, H3 and H4, nucleosomes are an essential component of chromatin and facilitate the regulation of gene expression and integrity of chromosomal DNA. Although nucleosome core particles can be adequately studied by methods such as FRET due to their compact size (<11 nm)^20^, their higher-order assemblies into chromatin structures are still poorly understood due to the lack of methods providing dynamic information at the nanoscale but beyond the Förster radius in living cells. STARSS can fill this gap by probing the global motion of chromatin fibers.

**Extended Data Fig. 1:**
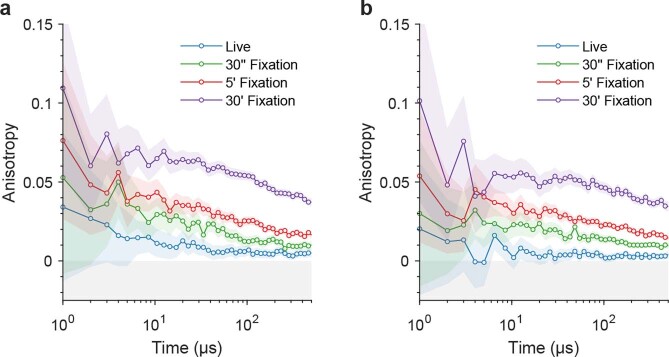
STARSS method 1 data of H2B-rsEGFP2 rigid and flexible labels. STARSS method 1 data of **(a)** rigid H2B-rsEGFP2 label and **(b)** flexible H2B-rsEGFP2 label in U2OS cells (each curve integrated over n = 8 cells). Living cells and a few conditions of fixation are shown. The times given in the legend refer to the incubation time with PFA. This is an extended dataset of Fig. 3c of the main manuscript, with same experimental conditions.

Applying STARSS method 1 on mammalian cells expressing the rigidly fused H2B-rsEGFP2, we observed twofold higher anisotropy values in interphase chromatin compared with a H2B-rsEGFP2 fusion construct with a classical flexible linker of the same number of amino acids (six amino acids (DPPVAT), >2.1 nm; Fig. 3c and Supplementary Information 11.3). No significant differences were observed using traditional steady-stated FA (Supplementary Information 12). To test the maximal anisotropy practically achievable under the given experimental conditions, we fixed both samples with 4% paraformaldehyde for up to 30 min. Rigidity increased with progressive crosslinking, as shown by increasing anisotropy values for both H2B-rsEGFP2 constructs (Fig. 3c and Extended Data Fig. 1). However, for all conditions, the flexible fusion protein showed lower anisotropy values reflecting the higher local mobility of rsFPs occurring within the first microseconds after the ON-switching pulse. Together, these results confirmed that the helix fusion strategy reduces rsEGFP2 local motion thereby increasing the sensitivity of STARSS experiments.

**Extended Data Fig. 2:**
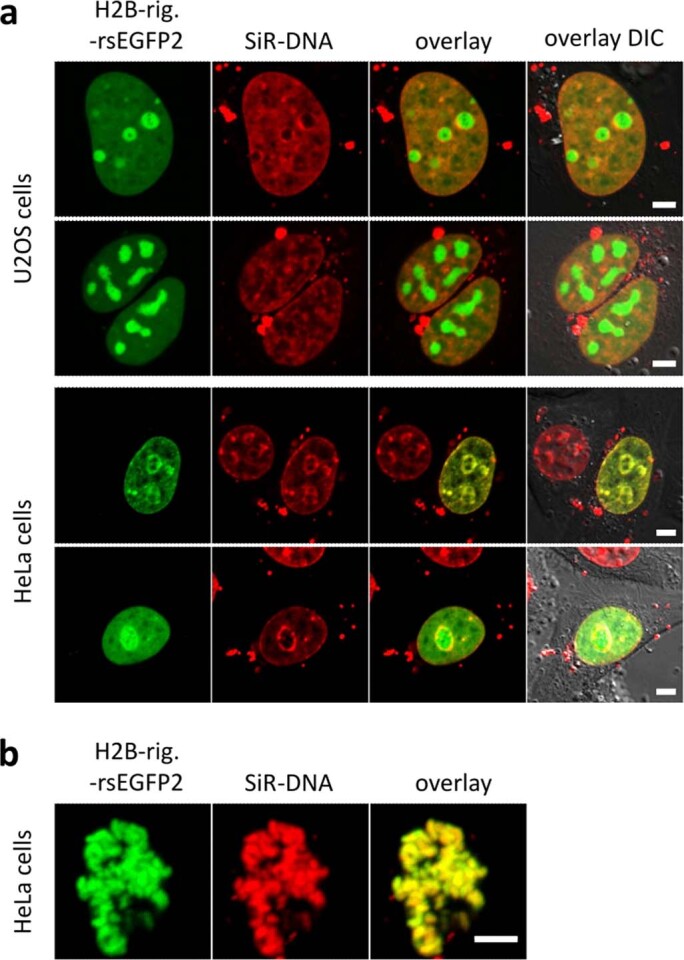
Chromatin staining in cells expressing H2B-rsEGFP2. U2OS and HeLa cells were transiently transfected with H2B-rig.-rsEGFP2 and stained with SiR-DNA before live cell confocal imaging. **(a)** The localization of rigidly fused H2B-rsEGFP2 correlates with chromatin structures and additional accumulation in nucleoli. Nucleoli are identified as subnuclear structures with low DNA but high protein density, which are surrounded by areas of high DNA density. **(b)** H2B-rsEGFP2 stays associated with DNA in condensed chromosomes during mitosis. Scale bar 5 μm.

Taking advantage of the newly designed histone probe, we set out to compare the rigidity of chromatin under different conditions in human cancer cell lines. Our marker protein was uniformly associated with chromatin throughout the nucleus but was also found in structures at higher concentration. These subnuclear compartments were confirmed to be nucleoli by costaining for DNA (Extended Data Fig. 2). Using intensity-based segmentation, we were able to observe roughly twofold higher anisotropy values in nucleoli compared with the remainder of the interphase nucleus (Fig. 3d). This reflects a reduced tumbling of nucleosome core particles within the densely packed and transcriptionally inactive heterochromatin regions typically associated with nucleoli, compared with euchromatin, which is more dynamic to facilitate transcriptional activity.

Next, we measured interphase nuclei under treatments that interfere with chromatin compaction. Depletion of ATP led to a marked increase in anisotropy due to the induced chromatin compaction, consistent with previous observations^20^. The reduced rotational mobility of nucleosomes was most pronounced in nucleoli and along the nuclear periphery (Fig. 3e,f, blue). The effect was completely reversed within 1 h after the treatment was stopped (Fig. 3e). In contrast, chromatin decondensation via treatment with the histone deacetylase inhibitor trichostatin A (TSA) had no effect on anisotropy values (Fig. 3e). These results suggest that loss of contacts between neighboring nucleosomes along a string of DNA does not cause dissociation of whole chromatin clusters, which are probably being held together by noncore histones and further scaffolding proteins.

**Extended Data Fig. 3:**
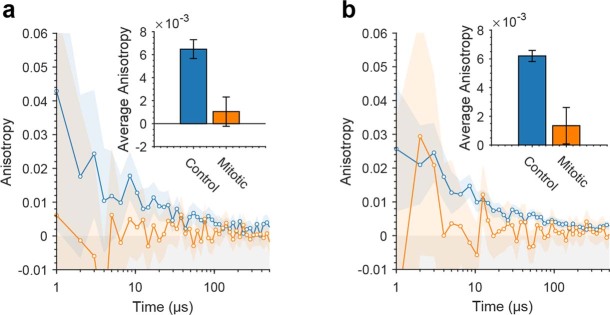
STARSS method 1 data of H2B-rsEGFP2 mitotic. STARSS method 1 data of **(a)** HeLa live cells and **(b)** U2OS live cells transfected with rigid H2B-rsEGFP2 (Hela control averaged over n = 8 cells, Hela mitotic averaged over n = 18).

Finally, we measured chromatin dynamics of fully condensed chromosomes in mitotic cells (Fig. 3e,f; Extended Data Fig. 2). The anisotropy values measured in mitotic chromosomes were significantly lower than those from interphase nuclei. The changes in chromatin dynamics were similar in U2OS and HeLa cells, but more pronounced in the latter (Extended Data Fig. 3). Fluorescence anisotropy decay curves exhibited a stretched exponential decay trend, suggesting no discrete or dominant range of chromatin cluster sizes under any tested condition. Thus, nucleosome core particles seem to be arranged in rotationally constrained clusters with a continuous spectrum of hydrodynamic diameters.

Our findings are in agreement with previous studies showing no differences in the diameter of chromatin fibers^21^ between chromosomes during mitosis or interphase, despite the higher compaction of nucleosomes^20^. Taken together, our STARSS data suggest that chromatin in mitotic chromosomes is arranged in fibers with densely packed nucleosomes, but that these fibers are arranged in flexible loops or loosely packed clusters that allow a wide range of rotational movements.

## STARSS studies of protein assemblies in viral particles and cells

**Fig. 4:**
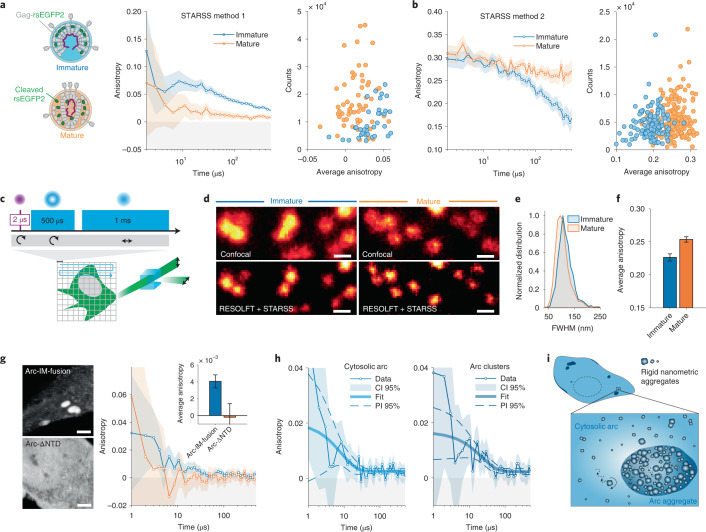
Oligomerization studies in viral particles and cells with STARSS. **a**, Anisotropy curves measured with STARSS method 1 in immature and mature HIV-1 VLPs labeled with rsEGFP2. Fluorophores are tightly constrained within the Gag lattice in immature particles (blue) but can diffuse within the particle upon proteolytic Gag maturation (orange). Average anisotropy values were extracted per individual VLP, showing two distinguishable distributions (right). **b**, Anisotropy curves measured with STARSS method 2 from HIV-1 labeled with rsEGFP2 (left) detected immature and mature particles in time-resolved mode with increased sensitivity. Single-particle average anisotropy values were extracted from small regions of interest including one VLP (right). **c**, Schematic representation of STARSS with super-resolution imaging. **d**, Images of mature and immature virions labeled with rsEGFP2 extracted from STARSS method 2 data. Confocal and RESOLFT super-resolution images are shown. Scale bars, 250 nm. **e**, Particle size distribution (full width at half-maximum, FWHM) of a Lorentzian fitting in the two conditions measured in super-resolved images (Supplementary Information 12). **f**, Average STARSS method 2 anisotropy values for immature and mature particles measured on RESOLFT images (integrated over 122 and 185 particles, respectively). **g**, Representative fluorescence images in grayscale of Arc and mutant (left, scale bars 4 μm) and fluorescence anisotropy curves (right). STARSS method 1 was applied to live HeLa cells expressing Arc-∆NTD-rsEGFP2 (averaged over *n* = 5 cells) and Arc-rsEGFP2-IM-fusion (averaged over *n* = 12 cells). **h**, Decay of fluorescence anisotropy recorded with STARSS method 1 for Arc-rsEGFP2-IM-fusion in the cytosol (left) and in micrometric clusters (right). Fitting using a monoexponential model is reported; fitting model and functional prediction interval (PI) are shown. **i**, Cartoon illustrating the nanometric aggregation of Arc in the cytosol and micrometric clusters. Diffusing rigid nanoparticles of tens of nanometers are present in both compartments, with higher tendency of aggregation in highly concentrated clusters. In all relevant panels, unless otherwise stated, error bars and shaded regions of anisotropy values are 95% confidence intervals (CIs) evaluated from detector noise according to Supplementary Information 20.

Another general strategy utilized to minimize the local motion of rsFPs targeted to a complex of known tertiary or quaternary structure is to impose rotational constrains via two anchoring points. This tagging strategy will be referred to as intramolecular (IM) fusion, since it works by insertion of the tag within an exposed loop or unstructured intramolecular region. To validate this approach, we inserted rsEGFP2 as a separate domain between the MA and CA domains within the structural polyprotein Gag in HIV-1 virus-like particles (VLPs; Fig. 4a). HIV-1 is initially released from cells in the form of immature particles, with a semispherical shell of ~2,400 Gag molecules lining the viral envelope. In this case rsEGFP2 is rigidly anchored within the Gag polyproteins, which form a tight hexagonal lattice whereby orientation of the fluorophores is highly constrained. Upon virus maturation, HIV-1 protease incorporated in the particle processes Gag into its mature subdomains, releasing rsEGFP2 from the tagged polyprotein^22^. This allows diffusion of the fluorophore molecules within the constraints of the particle, facilitating rapid tumbling of the fluorescence probe. We prepared mature VLPs tagged with rsEGFP2 from tissue culture supernatant of HEK293T cells transfected with proviral plasmids. To obtain immature particles, transfected cells were grown in the presence of the HIV-1 protease inhibitor lopinavir. The processing status was assessed by immunoblot (Supplementary Information 13). We then applied different STARSS methods to characterize the properties of immature and mature VLPs labeled with rsEGFP2.

Using STARSS method 1, immature particles displayed a slow degree of fluorophore rotation because rsEGFP2 is tightly packed in the lattice (Fig. 4a), showing average anisotropy values of ~0.03. In mature particles, however, cleaved rsEGFP2 rotated much faster, resulting in about threefold lower anisotropy values. By fitting the curves measured from VLPs in solution, we found a corresponding hydrodynamic radius of 101 ± 9 nm (Supplementry Information 14), in agreement with virion size distribution obtained by electron microscopy and super-resolution fluorescence images^22,23^. By switching most of the rsFPs (>80%, corresponding to ~1,950 fluorophores per particle) into the ON state we obtained a higher signal-to-noise ratio, further increasing the sensitivity of this method (Fig. 4b, right). The number of photons detected with STARSS method 2 was sufficient to further apply this method in combination with super-resolution microscopy (Fig. 4c–e). Because rsEGFP2 is a probe compatible with RESOLFT nanoscopy^2,24^, the size of immature and mature particles VLPs was subresolved with mean values of 115 ± 1.3 and 105 ± 1.5 nm, respectively (Fig. 4d and Supplementary Information 15). Based on the largely overlapping size distribution quantified in super-resolved RESOLFT images, mature and immature particles cannot be distinguished (Fig. 4e). By classifying the emitted photons with a polarization-sensitive system, as in STARSS methods 1 and 2, in the same measurement, we could detect a difference in average anisotropy values of mature and immature particles (Fig. 4f). While RESOLFT provides the spatial resolution needed to distinguish single VLPs, STARSS measures their rotational dynamics.

The IM-fusion tagging strategy can also be extended to the study of Gag-homologous proteins, which appear to retain the characteristic oligomerization tendency albeit being functionally repurposed throughout evolution. For example, Arc is pivotal to the creation of long-term memory in neurons^25^, and purified rat protein was recently found to oligomerize in vitro into capsid-like particles with a size distribution of 30–100 nm^26^. Nevertheless, the capability of Arc to form complexes in cellular environments has never been assessed. We used STARSS method 1 to quantify Arc oligomerization in the cellular environment.

rsEGFP2 was inserted into the full-length Arc protein downstream of the N-terminal domain (NTD) at the beginning of an unstructured linker region. The rotationally constrained Arc-rsEGFP2-IM-fusion construct was expressed in HeLa cells, resulting in a cytosolic homogeneous component and bright aggregates (Fig. 4g, top left). Arc-rsEGFP2-IM-fusion exhibits a clear exponential component in anisotropy decay with an approximate time constant of tens of microseconds (Fig. 4g, right), suggesting the presence of aggregates of tens of nanometers. As a control, we expressed Arc-∆NTD-rsEGFP2, an Arc mutant missing the NTD, preventing oligomerization^26^. Unlike the full-length protein, Arc-∆NTD-rsEGFP2 appears homogenously distributed in the cytosol (Fig. 4g, bottom left) with complete loss of anisotropy.

The full-length Arc anisotropy curves displayed rotational diffusion times of 22.2 ± 12.3 μs when measured in bright aggregates and 8.1 ± 6.4 μs in the remainder of the cytosol (Supplementary Information 16). Assuming uniform viscosity of water, these values translate to an apparent size of rigid nanometric particles of 55 ± 11 and 40 ± 12 nm, respectively (293 K, 1 cP; Fig. 4h), suggesting more pronounced clustering in bright aggregates compared with the cytosol (Fig. 4i). Low-copy-number oligomers are consistently present, as supported by the substantial fraction of anisotropy lost at time scales <1 µs. Together, our data support the tendency of Arc to form not only low-copy-number oligomers but also larger rigid assemblies in the tens of nanometers when expressed in living cells.

## Discussion and Conclusion

Fluorescence anisotropy has been widely employed in biomedical research as a tool to study protein–protein^27^, protein–DNA^28^ and DNA–DNA interactions^7^ with high throughput^29^, but only for small molecules. Here, we extended the potential applications of FA to larger macromolecular complexes (>30 kDa) with the use of reversible photoswitching. We demonstrated that ON–OFF state transitions can be efficiently photoselected and probed to overcome current limitations in TR-FA, namely its inability to probe orientation beyond fluorescence lifetime. We present three independent experimental approaches and analytical theoretical descriptions of STARSS with long-lived ON and OFF states, which can all be used to probe rotational diffusion and discriminate between static and dynamic objects of diverse sizes. Each method was developed as an optimal solution for specific applications. Method 1 is ideal for accurate quantification of slow rotational diffusion and robustness to photobleaching (Supplementary Information 17), method 2 maximizes the photon budget and sensitivity (Supplementary Information 18) and method 3 simplifies the hardware requirement, which is ideal for future development of easily accessible technology for screening studies.

Rotational diffusivity is a useful observable in cell biology, because it directly reflects changes in mass due to molecular interactions or in the viscosity of the local environment. By extending the potential to measure rotation at both microsecond and millisecond time scale, we can address the dynamics of higher-order structures such as chromatin fibers, thereby filling the gap between traditional FRET studies and electron microscopy or super-resolution imaging. While FRET is highly efficient in probing molecular interaction at short distances, it rapidly loses sensitivity above Förster radius >10 nm, precluding the study of higher-order molecular complexes. In contrast, STARSS can access a continuous size range especially if the three proposed methods are combined. Rigid labeling for STARSS can be easier to obtain than an efficient FRET pair, because only a single rsFP has to be placed at any position of the target protein that represents the protein or biocomplex rotation. For structural studies, FRET and STARSS can be highly complementary. In combination with FRET, STARSS can be used to measure the orientation of specific subunits and determine whether a minimal value of FRET efficiency is correlated to a fast tumbling of the probe rather than to longer distances. Additionally, pilot STARSS analysis can pinpoint optimal binding sites, which can be precisely targeted with FRET. STARSS can also be optimized for precise unraveling of molecular tumbling and orientation in three dimensions with the use of isotropic illumination patterns^30^.

The STARSS readout is also compatible with advanced microscopy methods, as shown by the simultaneous acquisition of super-resolution images and anisotropy data on VLPs, adding rapid dynamic information to the imaging data. The sensitivity of STARSS allowed detection of maturation states of the structural polyprotein Gag at the single-particle level. As a note, when studying large macromolecular complexes, the hydrodynamic radius extracted from STARSS curves is much less affected by labeling densities than super-resolution imaging. When rigidly attached, even a single fluorescent molecule carries the diffusion properties of the whole complex, allowing extraction of representative sizes of scarcely labeled particles. Instead, in super-resolved imaging, ideally, all molecules should be detected to show the correct size of an entire complex.

Other methodologies, including polarization-resolved fluorescence correlation spectroscopy (FCS), can also cover molecular motion within nanosecond–microsecond time scales, with the advantage of working with conventional fluorophores^31^. Nevertheless, these rely mainly on translational diffusion which is less sensitive to molecular size because it is inversely proportional to the hydrodynamic radius as opposed to the inverse cubic dependence of the rotational diffusion coefficient. Furthermore, unlike fluctuation-based FCS studies, STARSS has the ability to probe highly concentrated environments and it is scalable to large volumes. The STARSS signal is not restricted to the single-molecule regime because it does not lose information when averaged over large regions of interest or when probing small and bright aggregates. In fact, STARSS measurements demonstrated the tendency of the Gag-homologous protein Arc to oligomerize even in crowded subcellular regions not measurable with conventional FCS due to the difficulties in detection of signal fluctuations. Considering the tendency of Arc to oligomerize and bind RNAs, it is tempting to speculate that these regions might represent liquid–liquid-phase-separated condensates resulting from the high density of proteins and RNAs, in analogy to viral factories^32,33^.

Because the STARSS signal can be collected in large volumes, it has the potential to massively increase the throughput of studies, especially when coupled with wide-field, light-sheet or other parallelized illumination schemes, paving the way for future screening studies.

## Methods

### STARSS setup and analysis

#### STARSS setup

**Extended Data Fig. 4:**
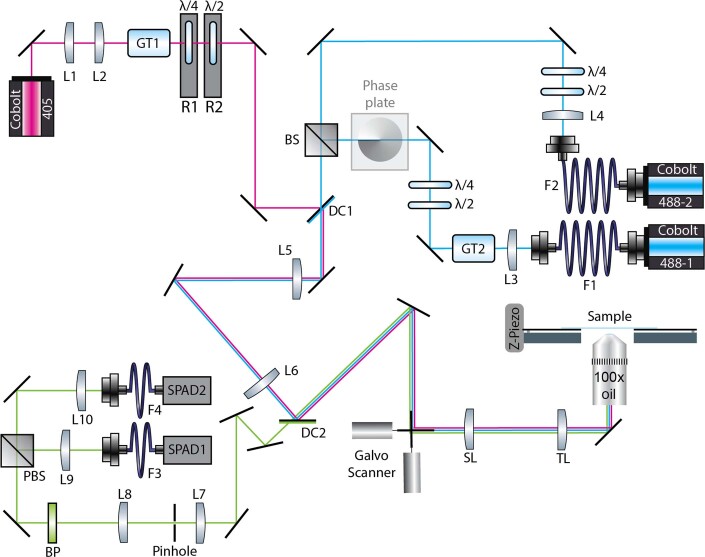
Microscopy setup used for STARSS signal acquisition. **Lenses**: L1: 19 mm, L3: 19 mm (AC127-019-A-ML, Thorlabs, Newton, NJ, USA), L2: 100 mm, L4: 30 mm, L5: 400 mm, L6: 400 mm, L7: 50 mm, L8: 200 mm, L9: 100 mm, L10: 50 mm (all AC254-XXX-A-ML, Thorlabs), SL: 50 mm (Leica Microsystems, Wetzlar, Germany), TL: 200 mm (Leica), OB1J: HC PL APO 100x/1.40 Oil STED White (Leica). **Filters**: BP: E535/70M (Chroma Technology, Bellows Falls, VT, USA). Dichroic mirrors}: DC1: FF556-Sdi01 (Semrock, NY, USA), DC2: ZT488rdc (Chroma). **Lasers**: 405: 06-01-MLD at 120 mW (405 nm, Cobolt AB, Solna, Sweden), 488-1 and 488-2: 06-01-MLD at 70 mW (488nm, Cobolt AB). **Detectors**: SPAD1: PD-100-CTD-FC (Micro Photon Devices, Bolzano, Italy), SPAD2: PD-050-CTB-FC (Micro Photon Devices). **Scanners**: Galvo Scanner XY: 6215H Galvanometric mirrors + 71215HHJ Servo Driver (Cambridge Technology, Bedford, MA, USA), Piezo Stage Z: LT-Z-100 (Piezoconcept, Lyon, France). **Fiber optics**: F3: FG105LGA (Thorlabs), F4: AFS50 (Thorlabs). **Polarization optics**: λ/4: 460-680 achr. (B. Halle Nachfl, Berlin, Germany), λ/2: 460-680 achr. (B. Halle Nachfl), PBS: CCM2- PBS251/M (Thorlabs), GT1 and GT2: GTH10M-A (Thorlabs). **Misc.**: R1 and R2: K10CR1/M (Thorlabs), Phase plate: VPP-1 (RPC Photonics, NY, USA), BS: CCM1-BS013/M (Thorlabs), Pinhole: P50HD (Thorlabs), detection mirrors: BB1-E02 (Thorlabs), all other mirrors: PF10-03-P01 (Thorlabs).

Signals and images were acquired using a custom-built setup originally presented in Dreier et al.^34^, and further modified for polarization measurements (Extended Data Fig. 4). Three modulable continuous-wave lasers (Cobolt) were combined into an *xy*-galvo scan setup (Cambridge Technology) and further directed into a Leica stand. Fine and independent control of polarization of each beam was accomplished with dedicated waveplates in each beam line. The polarization of the laser beams was changed according to the STARSS experiment to be performed. Fluorescence emission was descanned and collected with a pair of single-photon avalanche detectors (MPD) after splitting of the polarizing beam. The sample was moved in the *z*-direction using a piezo stage (Piezoconcept). The *xy*-galvo, the z-piezo and laser pulsing were controlled using a field-programmable gate array card (FPGA, PCIe-7852, National Instruments) and custom designed software in LabVIEW. The system can generate laser pulses as short as 50 ns, and time bins for photon counting as short as 10 ns. All reported pulse schemes were implemented in the same general-purpose control scheme with ten programmable modulation windows per laser. Moreover, a separate perpetual photon counter was coupled to a synchronized time window for photon binning, with an arbitrary number of time bins. Spatial scanning was also performed at the same time, and one or more pulse schemes could be performed at every spatial position. A more detailed description and schematic can be found in Supplementary Information 19. The acquisition parameters are reported in Supplementary Table 7.

#### STARSS data analysis

Fluorescence anisotropy observables *r* are computed using the following equation^6^:

$$r = \frac{{I_\parallel - GI_ \bot }}{{I_\parallel + 2GI_ \bot }},$$

where *I*_||_ and $I_ \bot$ are the counts obtained from the parallel and perpendicular detectors, respectively, defined according to the specific STARSS experiment. *G* is an experimental correction factor that removes the unbalance of the detectors and is measured using a fluorescence bar and a single, circularly polarized excitation beam. At the end of all STARSS experiments with long-lived states there is a long off-switching pulse that resets the system to the initial conditions. The fluorescent level at the end of the off-switching is the background due to the cross-talk. When computing *r* the background was subtracted from *I*_||_ and $I_ \bot$. The signal is acquired scanning the sample in *xy*, and thus includes the spatial information. The raw data from each detector are a three-dimensional array of photon count bins, with two dimensions spatial and the third temporal. In time-resolved fluorescence anisotropy curves and average anisotropy values, spatial dimensions are integrated. In H2B-rsEGFP2 data, fluorescence intensity thresholding was used to compute a spatial segmentation map of STARSS method 1 photons integrated according to segmented zones, generating the signal for both nucleoli and nucleus. Further details on data analysis and uncertainty estimation are reported in Supplementary Information 20.

### Sample preparation

#### Protein-decorated silica beads

Proteins rsEGFP2 and DronpaM159T were expressed in *Escherichia coli* BL21-RILP from pQE31 expression vectors and purified via Ni-nitriloacetic acid (Ni-NTA) affinity chromatography (HisTrap Fast Flow column, GE Healthcare) following the manufacturer’s instructions. Purified proteins were washed and concentrated in 100 mM Tris-HCl and 150 mM NaCl pH 7.5 by ultrafiltration.

Ni-NTA was used to functionalize silica beads (Micromod Partikeltechnologie; 10 g l^–1^) which were incubated for 30 min with rsEGFP2 protein in 100 mM Tris-HCl and 150 mM NaCl pH 7.5. Protein concentration varied depending on the size of the beads, and was tuned to be proportional to their total surface area. In particular, starting from a stock of protein of 50 μM, the concentration was reduced to have one protein every 150 nm^2^ of total bead surface.

Samples were measured in 4-μl drops deposited on coverslips pretreated with bovine serum albumin 1% for 15 min, and washed twice with buffer. To avoid drying of the drops, a closed chamber with silicon barriers was used (Ibidi) with the addition of 1 ml of buffer inside one of the sectors.

#### DronpaM159T and rsEGFP2 deposition on coverslips

Drops of purified protein (5 μl) of Dronpa159T and rsEGFP2 at 5 μM concentration were deposited on coverslips, which were precleaned with 99% ethylic alcohol. The samples were pressed on a slide and sealed with two-component dental glue. After 15 min, samples were measured. Physisorption of protein to the glass was sufficient to anchor a stable layer just below the glass interface.

#### Rigid tagging

To create H2B-rsEGFP2 (pDOS66), emiRFP703 was replaced in pH2B-emiRFP703 (VV020)^35^ with rsEGFP2. In brief, emiRFP703 was cut out using AgeI/NotI restriction enzymes and rsEGFP2 was PCR amplified from N-cadherin-rsEGFP2 (pDOS03); the two fragments were then ligated via Gibson assembly. The rigid linker modification for H2B-rigid-rsEGFP2 (pDOS73) was introduced via PCR amplification of two fragments from H2B-rsEGFP2 (pDOS66), which were subsequently assembled in a Gibson assembly reaction.

TOM20 and rsEGFP2 fragments were PCR amplified from plasmids mitoLAMA-F98 (Addgene, no. 130704) and UrsG7 (pDOS64, derived from plasmid UG7; Addgene, no. 135990) and ligated into the NheI/XhoI linearized plasmid backbone of UrsG7 to form TOM20-rigid-rsEGFP2 (pDOS79). Primers are detailed in Supplementary Information 21, with their respective template and target plasmids.

#### Cell transfection and fixation

HeLa cells (ATCC CCL-2) and U2OS cells (ATCC HTB-96) were maintained in DMEM (Thermo Fisher Scientific, no. 41966029) supplemented with 10% (*v/v*) fetal bovine serum (FBS; Thermo Fisher Scientific, no. 10270106) and 1% (*v/v*) penicillin/streptomycin (P/S; Sigma-Aldrich, no. P4333) in a humidified incubator at 37 °C and 5% CO_2_. One day before transfection, 10^4^ cells cm^–2^ were seeded on no. 1.5 glass coverslips in a six-well plate or chambered coverslips (Ibidi, no. 80827). Cells were transfected using FuGENE (Promega, no. E2311) according to the manufacturer’s protocol and maintained under standard conditions overnight. For STARSS measurement, the medium was replaced with phenol-red-free, CO_2_-independent imaging medium (Leibovitz’s L-15 Medium; Thermo Fisher Scientific, no. 21083027) with 10% (*v/v*) FBS and 1% (*v/v*) P/S).

For fixation experiments, transfected cells were washed with PBS solution followed by incubation in 4% (*w/v*) paraformaldehyde in PBS pH 7.4 for 0.5–30 min, washed 2x with PBS and finally measured in PBS.

#### Chromatin labeling

Cells on cover glasses were incubated with 2 µM SiR-DNA and 10 µM verapamil (both spirochrome, Switzerland) in complete imaging medium for 1 h and imaged live using a confocal microscope (Zeiss LSM 880 equipped with ×63 oil objective) after washing with complete imaging medium.

#### Chromatin (de)condensation

For ATP depletion (ATPd), cells were treated with 10 mM sodium azide and 50 mM 2-deoxyglucose mixed in complete medium for 1 h. Trichostatin A was administered at a final concentration of 200 ng ml^–1^ in complete medium 24 h before measurement.

#### Preparation of VLPs

Plasmid pCHIV^irsEGFP2^ was derived from plasmid pCHIV^36^, which comprises the complete HIV-1_NL4-3_ coding sequence except for *nef*. Deletion of both long terminal repeats renders this proviral derivative noninfectious. The rsEGFP2 coding sequence, flanked at both termini by coding sequence for two copies of SQNY-PIV HIV-1 PR cleavage sites, was amplified by PCR and inserted between the matrix and capsid-coding regions of the *gag* open reading frame via MluI and XbaI restriction sites, as previously described for other fluorescent proteins^37^. Virus-like particles were prepared as described in ref. ^38^. Briefly, HEK293T cells were seeded in six-well plates (3 × 10^5^ cells per well) and transfected the following day with an equimolar ratio of pCHIV and pCHIV-rsEGFP2 (2 µg per well) using polyethylenimine according to standard procedures. Cells were grown in the presence (immature particles) or absence (mature particles) of 2 µM lopinavir (LPV; no. Cay13854, BioMol). At 2 days post transfection the tissue culture supernatant was harvested, filtered through a 0.45-µm nitrocellulose filter and purified by ultracentrifugation through a 20% (*w/v*) sucrose cushion at 130,000 *g* for 90 min at 4 °C. Particle pellets were gently resuspended in storage buffer (PBS supplemented with 10 mM HEPES pH 7.4 and 5% FBS) and stored in aliquots at −80 °C. For immunoblot characterization, particle preparations were separated by SDS–polyacrylamide gel electrophoresis (15%, acrylamide/bis-acrylamide 200:1). Proteins were transferred to a nitrocellulose membrane by semidry blotting (0.8 A cm^–2^, 1 h). Particle-associated proteins were detected using polyclonal antisera raised against recombinant HIV-1 capsid (sheep; in house) or GFP (rabbit; in house) and secondary antibodies donkey anti-sheep IgG DyLight 680 (no. 613-744-168, Rockland Immunochemicals) or IRdye800CW donkey anti-rabbit IgG (no. 926-32213; LI-COR Biosciences), respectively. Bound antibodies were detected using a LI-COR CLx infrared scanner.

#### HIV-1 samples for STARSS

To adhere VLPs, coverslips were cleaned with absolute ethanol, air dried and treated with poly-l-lysisne 0.1% in water (with 0.01% thimerosal as preservative; Sigma-Aldrich) for 15 min, then washed twice with buffer (100 mM Tris-HCl and 150 mM NaCl pH 7.5). One drop (4 μl) of HIV-1 samples was deposited on coverslips; after 15 min the drop was removed and 4 μl of buffer added. Samples for measurement of HIV-1 particles freely diffusing in solution were prepared by placing one drop (4 μl) of sample on a coverslip. All samples were prepared in closed chambers with silicon sectors (Ibidi), with the addition of 1 ml of buffer in one of the sectors to avoid drying.

#### Preparation of Arc samples

The plasmids used for Arc exogenous expression in HeLa cells were pAAV-EF1a_Arc-IM-fusionfusion-rsEGFP2-IRES-WGA-Cre and pAAV-EF1a_Arc-∆NTD-rsEGFP2-IRES-WGA-Cre. rsEGFP2 was subcloned between amino acid residues 137 and 138 for the IM-fusion construct. The Arc-∆NTD-rsEGFP2 mutant is missing amino acid residues 1–129, and rsEGFP2 is subcloned at the C terminus of the protein via the short linker GSGSGS. Plasmids were prepared from transformants and verified via Sanger sequencing. Primers are given in Supplementary Information 21, with their respective template and target plasmids.

For transfection, 2 × 10^5^ HeLa cells per well were seeded on coverslips in a six-well plate. After 1 day cells were transfected using FuGENE (Promega, no. E2311) according to the manufacturer’s instructions. Cells were then washed in PBS solution, placed in a chamber with imaging medium and imaged at room temperature.

#### Spatial segmentation of Arc data

Photons contributing to the STARSS method 1 signal were split into cytosol and micrometric clusters based on the intensity of the corresponding spatial images. The total counts obtained from a scanning point were normalized using the maximum signal from the brightest cluster in the field of view. Thus, cytosolic pixels were obtained from a 0.05–0.30 threshold and micrometric cluster pixels from values >0.5. Spatially segmented STARSS decays were then fitted using a monoexponential model including a constant static component.

### Reporting summary

Further information on research design is available in the Nature Research Reporting Summary linked to this article.

## Online content

Any methods, additional references, Nature Research reporting summaries, source data, extended data, supplementary information, acknowledgements, peer review information; details of author contributions and competing interests; and statements of data and code availability are available at 10.1038/s41587-022-01489-7.

## Supplementary information

Supplementary Information Supplementary Information 1–21.

Reporting Summary

## Data Availability

Sample raw datasets that support the findings of this study are available at 10.5281/zenodo.7010471, including data processing scripts. Any other data can be obtained from the corresponding author upon reasonable request.

## Code availability

The simulation tool for fluorescence anisotropy experiments with reversible switchable kinetics is available at https://github.com/TestaLab/rotational_diffusion_photophysics and 10.5281/zenodo.7009614. All other custom scripts will be made available upon request.
