# Supplementary material for: Extending fluorescence anisotropy to large complexes using reversibly switchable proteins
Source: Nat Biotechnol. 2022 Oct 10;41(4):552–9. doi: 10.1038/s41587-022-01489-7 (PMC10110461; doi:10.1038/s41587-022-01489-7)
Supplement: Supplementary file 1 — Supplementary Information 1–21. [file 41587_2022_1489_MOESM1_ESM.pdf]

nature biotechnology

Article https://doi.org/10.1038/s41587-022-01489-7

Extending fluorescence anisotropy to large complexes using reversibly switchable proteins

In the format provided by the authors and unedited

Supplementary Information

Breaking the mass limits of fluorescence anisotropy with reversibly switchable states

Andrea Volpato[1], Dirk Ollech[1], Jonatan Alvelid[1], Martina Damenti[1], Barbara Müller[2], Andrew York[3], Maria Ingaramo[3], and Ilaria Testa[1]

[1] *Department of Applied Physics and Science for Life Laboratory, KTH Royal Institute of Technology, 100 44 Stockholm, Sweden*
[2] *Department of Infectious Diseases, Virology, Centre for Integrative Infectious Disease Research (CIID), University Hospital Heidelberg, , D-69120 Heidelberg, Germany*
[3] *Calico Life Sciences LLC, South San Francisco, CA 94080, USA*

Contents

S1 STARSS method 1 size of silica beads coated with rsE-FGP2 and monoclonal antibodies 6
S1.1 Particle size from rotational diffusion time 7
S1.2 Mono-exponential model for anisotropy decays 7

S2 Analytical models for rotational diffusion and reversibly switchable states kinetics 9
S2.1 Time evolution of orientational probabilities 9
S2.2 Spherical harmonics expansion 10
S2.3 Solution of rigid isotropic rotational diffusion 11
S2.4 Coupling of diffusion with reversibly switchable states kinetics . 12
S2.5 Analytical solution of the full problem 13
S2.6 High-NA photo-selection 14
S2.7 High-NA polarized detection 15
S2.8 Four states model for negative rsFPs 16
S2.9 Eight states model for negative rsFPs 18
S2.10 Parametrization of rsEGFP2 19

S3 STARSS method 1 simulation of experiments 21

S4 STARSS method 1 size of silica beads 26

S5 STARSS method 1 and anisotropy variance 27

S6 STARSS method 2 simulation of experiments 32

S7 STARSS method 2 fit of silica beads data 35

S8 High-power OFF-switching photo-physics of rsEGFP2 42

S9 STARSS method 3 simulation of experiments 45

S10 Saturation curve of ON-switching photo-physics of rsEGFP2 48

S11 Structural predictions for rigid fusion of rsEGFP2 49
S11.1 OMP25 50
S11.2 TOM20 52
S11.3 H2B 54

S12 STARSS method 1 compared with steady-state FA for H2B-rsEGFP2 rigid and flexible labels 56

S13 HIV-1 virus-like particle maturation assessment 57

S14 STARSS method 1 size of HIV-1 virus-like particle 58

S15 RESOLFT imaging size of HIV-1 virus-like particles 59

S16 STARSS method 1 fit of Arc-rsEGFP2-IM-fusion data . . 64

S17 Photo-bleaching in STARSS experiments 65

S18 Sensitivity of STARSS for distinguishing complexes of similar sizes 68

S19 STARSS with long-lived states setup 70
S19.1 Control of the setup using an FPGA 70

S20 Notes on data analysis and anisotropy variance estimation 73
S20.1 Estimation of anisotropy variance 73
S20.2 Log-scale time binning of counts 74
S20.3 Average and time resolved anisotropy data 74

S21 Primer list 76

References 78

List of Figures

S1 Fit of STARSS method 1 data of beads and antibodies 6
S2 Illustration of the transition dipole moment orientation and the laboratory fixed three dimensional reference frame. 10
S3 Kinetic models for negative rsFPs 17
S4 Power density used in the simulation of STARSS method 1 . . . 21
S5 Simulated detector signals of STARSS method 1 22
S6 Simulated fluorescence anisotropy decays of STARSS method 1 . 23
S7 Time evolution of orientational probabilities for STARSS method 1 24
S8 Simulated STARSS method 1 signal and variance changing t_H . . 28
S9 Simulated STARSS method 1 signal and variance changing I_0 and I_{bg} 29
S10 Simulated STARSS method 1 signal and variance changing r_0 . . 30
S11 Simulated STARSS method 1 signal and variance changing τ_R . . 31
S12 Power density used in the simulation of STARSS method 2 . . . 32
S13 Simulated detector signals of STARSS method 2 33
S14 Simulated fluorescence anisotropy decays of STARSS method 2 . 34
S15 Time evolution of orientational probabilities for STARSS method 2 34
S16 Fit of STARSS method 2 data of rsEGFP2 in solution 37
S17 Fit of STARSS method 2 data of 500 nm beads 38
S18 Fit of STARSS method 2 data of 30 nm beads 39
S19 Fit of STARSS method 2 data of 50 nm beads 40
S20 Fit of STARSS method 2 data of 100 nm beads 41
S21 High-power OFF-switching photo-physics of rsEGFP2 43
S22 Power density used in the simulation of STARSS method 3 . . . 46
S23 Simulated detector signals of STARSS method 3 46
S24 Simulated signals of STARSS method 3 47
S25 ON-switching saturation curve of rsEGFP2 48
S26 rsEGFP2 crystal structure. 49
S27 Protein sequence for rsEGFP2-OMP25. 51
S28 Protein sequence for rigidly fused TOM20-rsEGFP2. 53
S29 Crystal structure of a nucleosome 54
S30 Protein sequence for the rigidly fused H2B-rsEGFP2. 55
S31 Protein sequence for the flexibly fused H2B-rsEGFP2. 55
S32 STARSS method 1 and steady-state FA data of labeled H2B in live cells 56
S33 Immunoblot characterization of virus-like particles 57
S34 Fit of STARSS method 1 decay of HIV-1 virus-like particles . . . 58
S35 Confocal and RESOLFT images of mature HIV-1 virus-like particles 60
S36 Confocal and RESOLFT images of immature HIV-1 virus-like particles 61
S37 Fitting of RESOLFT images of mature HIV-1 virus-like particles 62
S38 Fitting of RESOLFT images of immature HIV-1 virus-like particles 63
S39 Effect of photo-bleaching in STARSS method 1 experiments . . . 66

S40 Effect of photo-bleaching in STARSS method 2 experiments . . . 67
S41 Simulations of STARSS method 1 and 2 for complexes of similar size 69

List of Tables

S1 Fitting parameters of STARSS method 1 data of beads and antibodies 7
S2 Fitting parameters of STARSS method 1 data of beads 26
S3 Fitting parameters of STARSS method 2 data of beads 36
S4 Fitting parameters of OFF-switching kinetics of rsEGFP2 44
S5 Fitting parameters of STARSS method 1 data of HIV-1 virus-like particles 58
S6 Fitting parameters of STARSS method 1 data of Arc 64
S7 STARSS with long-lived states experimental conditions 72

S1 STARSS method 1 size of silica beads coated with rsEFGP2 and monoclonal antibodies

A sample of 100 nm silica beads bearing rsEGFP2 on the surface attached through Ni-NTA-HisTag was measured with STARSS method 1. The experiments was repeated on a second bead sample of the same type but with the addition of monoclonal antibody IgG-anti-GFP (clone EPR14104, abcam, ab 183734). A 1:1 stoichiometric amount of IgG:rsEGFP2 was used. As the mass of the sample slightly changed due to antibody binding, the rotational diffusion slowed down. In turn, the STARSS curve showed a slower trend compared to the sample missing the antibody, demonstrating the ability to detect small size variations in the tens of nanometers.

The pulse scheme was defined as: 3 ms of circularly polarized 488 nm light at 21.5 kW/cm^2 and 250 ns of linearly polarized 405 nm light at 109 kW/cm^2 after a delay of 1.5 ms. The result of the fitting gives a difference of roughly 16 nm between the two samples. Due to the sample preparation, the beads without and with antibody were probed in slightly different buffers, in aqueous buffer and 32% glycerol buffer respectively. This was taken into account by using the appropriate viscosity in the calculation of the size (Table S1). In the two buffers slightly different photo-physics were observed, as shown in Figure S1c. However, a modulation of the intensity of the curves does not influence the results because of the intrinsic definition of anisotropy. The most striking difference is the longer rise time of fluorescence in glycerol buffer, which could be induced by slower protonation transfer processes in this medium. Moreover the starting anisotropy is decreased in glycerol buffer, which could be because of an increased local mobility of rsEGFP2 on the surface of the beads or reduced Ni-NTA-HisTag binding affinity.

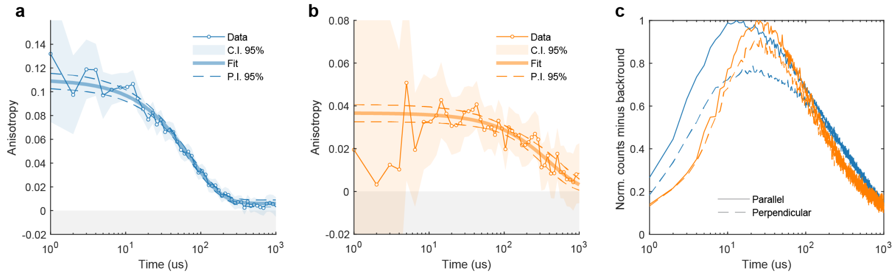

Figure S1: STARSS method 1 data of (a) 100 nm beads coated with rsEGFP2 and (b) 100 nm beads coated with rsEGFP2 and monoclonal antibodies. (c) Normalized fluorescence signals recorded in the p- and s-polarized detection channels for a water (blue) and glycerol (orange) bead sample buffer.

Sample	τ_R (μs)	r_0	r_∞	d (nm)
100 nm	72.4 (3.3)	0.104 (0.002)	0.0061 (0.0010)	82.4 (1.25)
100 nm + IgG	410 (52)	0.036 (0.016)	0.000 (0.017)	98.9 (4.1)

Table S1: Silica beads mono-exponential fitting of data reported in Figure S1. Parameter values and standard errors between brackets (expressed as the square root of the estimated variance from the Jacobian of the residuals of the fit). The model function is reported in eq. S5. For calculations of the sizes with eq. S3, 1.0049 cP was used for aqueous environment at 20°C, and 3.2763 cP for 32% glycerol aqueous environment at 20°C.

S1.1 Particle size from rotational diffusion time

Assuming an object with a spherical shape, the following Einstein-Stokes equations can be used

$$D_R = \frac{k_B T}{8\pi\eta r^3}, \tag{S1}$$

where D_R is the rotational diffusion coefficient, k_B is the Boltzmann constant, η is the viscosity of the medium, and r is the hydrodynamic radius of the spherical object. The observed rotational diffusion time constant can also be related to the rotational diffusion coefficient as

$$D_R = \frac{1}{6\tau_R}, \tag{S2}$$

where τ_R is the observed rotational diffusion time constant. The diameter d of the object as a function of the diffusion time is then retrieved directly as

$$d(\tau_R) = \sqrt[3]{\frac{6k_B T\tau_R}{\pi\eta}}. \tag{S3}$$

When not differently stated, calculations in the manuscript are performed with viscosity of water at 20°C, $\eta^{20°\mathrm{C}}_{\mathrm{water}} = 0.0010049$ Ns/m^2.

Given the uncertainty of τ_R expressed as its variance $\sigma^2_{\tau_R}$, we can propagate the uncertainty to the diameter of the object using the equation

$$\begin{aligned}\sigma_d^2 &= \left(\frac{\mathrm{d}d(\tau_R)}{\mathrm{d}\tau_R}\right)^2 \sigma^2_{\tau_R} \\ &= \left(\frac{\left(\frac{6k_B T}{\pi\eta}\right)^{1/3}}{3\tau_R^{2/3}}\right)^2 \sigma^2_{\tau_R}.\end{aligned} \tag{S4}$$

S1.2 Mono-exponential model for anisotropy decays

The model function used in the fitting of experimental fluorescence anisotropy decays is specified as

$$r(t) = r_0 \exp(-t/\tau_R) + r_\infty \tag{S5}$$

where $r(t)$ is the anisotropy observable, r_0 is the amplitude of the anistotropy decay, and r_∞ is the residual constant anisotropy. The fitting was biased using $1/\mathrm{Var}(r)$ as weights.

S2 Analytical models for rotational diffusion and reversibly switchable states kinetics

In this section the description of polarized fluorescence experiments of photo-reactive systems in solution will be discussed. In particular we will focus our interest on fluorescence signals produced by reversible switchable fluorescent proteins (rsFPs) and dyes for STED microscopy. We will use a rigid isotropic model for rotational diffusion, and we will assume that the diffusive properties of our system are conserved for all the species in the photo-physics kinetic scheme. We are also assuming that the orientation of the molecule can be identified with the orientation of the transition dipole moment, which is conserved in the same orientation for every species. We are seeking an analytical solution of the diffusive problem, that is suitable for model fitting of experimental data.

S2.1 Time evolution of orientational probabilities

The conditional probability of a species ϵ, starting with orientation (θ_0, ϕ_0) at time $t = 0$ from any possible species, and being at orientations (θ, ϕ) at time t, is specified as $p_\epsilon(t, \theta, \phi | t = 0, \theta_0, \phi_0)$, or for shortness $p_\epsilon(t, \theta, \phi)$. Definitions of the angles are illustrated in the sketch in figure S2. Following the theory developed in [1, 2], the time evolution of the orientational conditional probabilities can be specified using a set of differential equations as

$$\frac{\partial p_\epsilon(t, \theta, \phi)}{\partial t} = D_R \nabla^2 p_\epsilon(t, \theta, \phi) + \sum_\eta k_{\epsilon\eta}(\theta, \phi) p_\eta(t, \theta, \phi), \tag{S6}$$

where D_R is the rotational diffusion coefficient for a rigid isotropic system, ∇^2 is the square gradient in the angular space, defined as

$$\nabla^2 p_\epsilon(t, \theta, \phi) = \frac{1}{\sin(\theta)} \frac{\partial}{\partial \theta} \left(\sin(\theta) \frac{\partial p_\epsilon(t, \theta, \phi)}{\partial \theta} \right) + \frac{1}{\sin^2(\theta)} \frac{\partial^2 p_\epsilon(t, \theta, \phi)}{\partial^2 \phi}, \tag{S7}$$

and $k_{\epsilon\eta}$ is the kinetic constant for the reaction that transform the η-th species into the ϵ-th species when $\eta \neq \epsilon$, i.e. $\eta \to \epsilon$. The kinetic constant $k_{\epsilon\epsilon}$ where both indexes are the same is different than the others. It refers to the depopulation of state ϵ, and it is computed summing all the possible rates that can remove population from that state

$$k_{\epsilon\epsilon}(\theta, \phi) = -\sum_{\eta \neq \epsilon} k_{\eta\epsilon}(\theta, \phi). \tag{S8}$$

In our problem, kinetic constants might depend on the orientation of the fluorophore, in particular this happens if the transition is induced by light-matter interaction. Moreover, in eq. S6, we can recognize two main terms: the first term models the free isotropic rotational diffusion of the system, while the second term models the kinetics and photo-physics of the system.

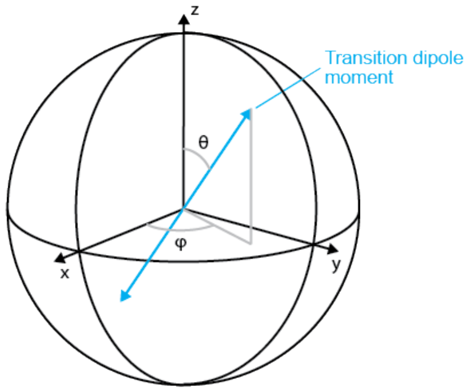

Figure S2: Illustration of the transition dipole moment orientation and the laboratory fixed three dimensional reference frame.

S2.2 Spherical harmonics expansion

All the angular functions can be expanded using a spherical harmonics (SH) basis. This approach has several advantages because SH are well behaving functions and they can simplify many aspect of the solution of the kinetic-diffusive problem. In particular the SH basis set is orthogonal and complete, and there are simple expressions to calculate products of SH functions [3, 4]. Orientational probabilities and kinetic constants can be expanded as

$$\begin{aligned} p_\epsilon(t,\theta,\phi) &= \sum_{l=0}^{\infty}\sum_{m=-l}^{+l} p_{lm}^{\epsilon}(t)Y_{lm}(\theta,\phi), \\ k_{\epsilon\eta}(\theta,\phi) &= \sum_{l=0}^{\infty}\sum_{m=-l}^{+l} k_{lm}^{\epsilon\eta}Y_{lm}(\theta,\phi) \end{aligned} \tag{S9}$$

where $Y_{lm}(\theta,\phi)$ are the spherical harmonics functions of order l and m, $p_{lm}^{\epsilon}(t)$ are the SH coefficients for the expansion of the orientational probability of state ϵ, and $k_{lm}^{\epsilon\eta}$ are the coefficients for the expansion of the kinetic constants for the $\eta \to \epsilon$ reaction. The expansions in eq. S9 separate the angular dependence that is fully embedded in the SH basis set, from the temporal dependence in the coefficients that weight the SH basis set. In general, if a function contains sharp angular shapes higher l values will be needed to fully map its content on the SH basis set. There is an analogy with the expansion in Fourier series, in which function with higher frequency content will require higher order sinusoidal functions to be expanded completely. In this sense SH are mapping higher angular frequency content for higher l values. Since the SH basis set is complete, any orientational probability can be expanded. Unconventional orientational probabilities with very sharp shapes could be achieved for example through saturation of photo-physics transitions.

Linear light-matter interaction introduces a substantial simplification on the expansion of kinetic constants of light driven processes, e.g. the transition from ground state to first excited state of a fluorophore. For all linear processes the probability of transition is proportional to the square of the scalar product of the electric field vector of radiation and the transition dipole moment of the fluorophore [5]. This dependence directly implies that only $l = 0$ and $l = 2$ are involved in the expasion in eq. S9. Moreover, if a kinetic constant does not depend on molecular orientation the expansion collapse just on the $l = 0$ and $m = 0$ term. Thus we can get these simplified expressions

$$\begin{aligned} k_{\epsilon\eta}(\theta,\phi) &= \sum_{l=0,2}\sum_{m=-l}^{+l} k_{lm}^{\epsilon\eta} Y_{lm}(\theta,\phi), && \text{(light-induced)} \\ k_{\epsilon\eta}(\theta,\phi) &= k_{00}^{\epsilon\eta}\, Y_{00}(\theta,\phi) = k_{\epsilon\eta}. && \text{(orientation-independent)} \end{aligned} \tag{S10}$$

Note that when using "4π" normalized SH functions $Y_{00}(\phi,\theta) = 1$, and $k_{00}^{\epsilon\eta} = k_{\epsilon\eta}$. This simplification greatly reduces the computation needed when solving eq. S6. If higher order light-matter interaction are present analogous simplifications are possible, e.g. two photon absorption only involve SH terms with $l = 0, 2, 4$.

S2.3 Solution of rigid isotropic rotational diffusion

Spherical harmonics expansion is convenient in the context of finding a solution for rotational diffusion problems [2], and in particular for isotropic rotational diffusion, i.e. the rotational diffusion of a rigid sphere without preferential axis of rotation. Spherical harmonics are eigen-functions of the rigid rotational diffusion operator, thus if we rewrite the free rotational diffusion problem in eq. S6 with the expansion in eq. S9, excluding for now the kinetics induced by photo-physics, we get

$$\begin{aligned} \frac{\partial p_\epsilon(t,\theta,\phi)}{\partial t} &= D_R \nabla^2 p_\epsilon(t,\theta,\phi) \\ \sum_{lm} \frac{\partial p_{lm}^\epsilon(t)}{\partial t} Y_{lm}(\theta,\phi) &= \sum_{lm} -D_R\; l(l+1)\; p_{lm}^\epsilon(t) Y_{lm}(\theta,\phi). \end{aligned} \tag{S11}$$

It can be shown that to solve this set of differential equations it is only necessary to solve the differential equations on the expansion coefficients [1]. Thus we can rewrite a new set of differential equations

$$\frac{dp_{lm}^\epsilon(t)}{dt} = -D_R\; l(l+1)\; p_{lm}^\epsilon(t). \tag{S12}$$

From this set of equations we can compute the solution for the SH probability coefficients, obtaining an exponential decay of each $p_{lm}^\epsilon(t)$ coefficient starting from the initial conditions $p_{lm,0}^\epsilon = p_{lm}^\epsilon(t = 0)$,

$$p_{lm}^\epsilon(t) = p_{lm,0}^\epsilon\; e^{-t/\tau_R}, \tag{S13}$$

where the relaxation times of the diffusion terms are specified as $\tau_R = 1/D_R\, l(l+1)$. A consequence coming from this solution, and the fact that linear light-matter interaction involves only SH terms with $l = 0, 2$, is that free rotational diffusion of an out of equilibrium orientational state prepared in linear regime exhibits a decay time of $\tau_R = 1/D_R 2(2+1) = 1/6D_R$ and a stationary component.

S2.4 Coupling of diffusion with reversibly switchable states kinetics

The coupling between rotational diffusion and kinetics induced by photo-physics is embedded in each kinetic contribution "rate × population" of eq. S6. By expanding on the SH basis set, we can greatly simplify the effort of computing the coupling of the two dynamics. Then using eq. S9 we can write

$$\begin{aligned} k_{\epsilon\eta}(\theta,\phi)p_\eta(t,\theta,\phi) &= \sum_{l_1 m_1} k^{\epsilon\eta}_{l_1 m_1} Y_{l_1 m_1}(\theta,\phi) \sum_{l_2 m_2} p^{\eta}_{l_2 m_2}(t) Y_{l_2 m_2}(\theta,\phi) \\ &= \sum_{l_1 m_1}\sum_{l_2 m_2} k^{\epsilon\eta}_{l_1 m_1} p^{\eta}_{l_2 m_2}(t)\; Y_{l_1 m_1}(\theta,\phi) Y_{l_2 m_2}(\theta,\phi). \end{aligned} \tag{S14}$$

Spherical harmonics have nice properties that we can exploit. In particular, since the SH basis set is complete, the product of two SH functions can be expressed as an expansion weighted by Wigner-3j coefficients [6]:

$$\begin{aligned} Y_{l_1 m_1}(\theta,\phi) Y_{l_2 m_2}(\theta,\phi) =& \sum_{lm} \sqrt{(2l_1+1)(2l_2+1)(2l+1)} \\ & \times \begin{pmatrix} l_1 & l_2 & l \\ m_1 & m_2 & -m \end{pmatrix} \begin{pmatrix} l_1 & l_2 & l \\ 0 & 0 & 0 \end{pmatrix} (-1)^m Y_{lm}(\theta,\phi), \end{aligned} \tag{S15}$$

where $\begin{pmatrix} l_1 & l_2 & l \\ m_1 & m_2 & m \end{pmatrix}$ is a Wigner-3j coefficient. The mathematics involved in the product of spherical harmonics is formally similar to the coupling of angular momenta in quantum mechanics, where two angular momenta l_1 and l_2 are summed giving the total angular momentum l. In this sense part of the mathematical background is shared also when rotational diffusion and kinetics are "summed" together.

We can rewrite the kinetic term in eq. S14, using eq. S15, and giving

$$k_{\epsilon\eta}(\theta,\phi)p_\eta(t,\theta,\phi) = \sum_{lm}\sum_{l_2 m_2} k^{\epsilon\eta}_{lm,l_2 m_2} p^{\eta}_{l_2 m_2}(t) Y_{lm}(\theta,\phi), \tag{S16}$$

where we defined a new set of kinetic constants $k^{\epsilon\eta}_{lm,l_2 m_2}$, that embed the algebra of the products of SH. In other words, the full description of the coupling of rotational diffusion and kinetics is found in the definition of these kinetics

constants, and they can be specified as

$$k^{\epsilon\eta}_{lm,l_2m_2} = \sum_{l_1m_1} k^{\epsilon\eta}_{l_1m_1} \sqrt{(2l_1+1)(2l_2+1)(2l+1)} \\ \times \begin{pmatrix} l_1 & l_2 & l \\ m_1 & m_2 & m \end{pmatrix} \begin{pmatrix} l_1 & l_2 & l \\ 0 & 0 & 0 \end{pmatrix} (-1)^m. \tag{S17}$$

Note that the kinetic constant $k^{\epsilon\eta}_{lm,l_2m_2}$ should be interpret as the rate that connects the l_2m_2 SH coefficient of the orientational probability of the reagent species η to the lm SH coefficient of the orientational probability of the product species ϵ. Furthermore, in this picture it is convenient to organize the set of kinetic constants $k^{\epsilon\eta}_{lm,l_2m_2}$ in a matrix $\mathbf{K}$. The two indexes of matrix elements K_{ij} are meant to explore kinetic species and SH coefficients, such that i indexes all the possible combinations of (ϵ, l, m) and j indexes all the possible combinations of (η, l_2, m_2). The structure of matrix $\mathbf{K}$ can also be subdivided in blocks $\mathbf{K}^{\epsilon\eta}$ that include all the terms involved in the $\eta \to \epsilon$ transition.

When the kinetics constant does not depend on the orientation of the fluorophore only the terms with $l_1 = 0$ and $m_1 = 0$ survive in eq. S17. As a consequence the expression is simplified as

$$k^{\epsilon\eta}_{lm,l_2m_2} = k_{\epsilon\eta}\ \delta_{l,l_2}\delta_{m,m_2}, \tag{S18}$$

which implies that the kinetic block for an orientational-independent kinetic constant is diagonal and can be specified as $\mathbf{K}^{\epsilon\eta} = \mathbf{1}k_{\epsilon\eta}$ where $\mathbf{1}$ is the identity matrix.

S2.5 Analytical solution of the full problem

The SH expansions in eq. S9 and the kinetic term in eq. S16 can be directly plugged in eq. S6 and a new set of differential equation for the full problem is is retrieved,

$$\frac{\partial p^{\epsilon}_{lm}(t)}{\partial t} = -D_R\ l(l+1)\ p^{\epsilon}_{lm}(t) + \sum_{l_2m_2} k^{\epsilon\eta}_{lm,l_2m_2} p^{\eta}_{l_2m_2}(t), \tag{S19}$$

that is derived analogously to eq. S12, reducing the full set of differential equations to differential equations only on the SH coefficients of the orientational probabilities.

We can collect all the rotational diffusion rates on the diagonal of matrix $\mathbf{D}$, indexed in the same way as matrix $\mathbf{K}$, such that $D_{ii} = -D_R l(l+1)$. Analogously, we can collect all the SH probability coefficients p^{ϵ}_{lm} in a vector $\mathbf{p}$ sharing the same indexing of matrices $\mathbf{D}$ and $\mathbf{K}$. In this way we can simplify the notation rewriting eq. S19 as

$$\frac{d\mathbf{p}(t)}{dt} = \mathbf{D}\mathbf{p} + \mathbf{K}\mathbf{p} \\ \frac{d\mathbf{p}(t)}{dt} = \mathbf{M}\mathbf{p}, \tag{S20}$$

where the matrix $\mathbf{M} = \mathbf{D} + \mathbf{K}$ collects all the rotational diffusion rates and kinetic rates. Finally, we can write the solution of this equation, in analogy to eq. S13, as

$$\mathbf{p}(t) = e^{\mathbf{M}t}\mathbf{p}_0, \tag{S21}$$

where $\mathbf{p}_0$ is a vector that collects all the coefficients $p^{\epsilon}_{lm,0}$ defining the starting conditions of the system. Note that the matrix exponential can be conveniently computed by diagonalizing matrix $\mathbf{M}$, obtaining

$$e^{\mathbf{M}t} = \mathbf{U}e^{\mathbf{\Lambda}t}\mathbf{U}^{-1}, \qquad \text{with} \quad \mathbf{M} = \mathbf{U}\mathbf{\Lambda}\mathbf{U}^{-1}, \tag{S22}$$

where the exponential of the diagonal matrix $\mathbf{\Lambda}t$ is again a diagonal matrix with elements $e^{\Lambda_{ii}t}$, $\mathbf{U}$ is the eigenvectors matrix, and $\mathbf{\Lambda}$ is the diagonal eigenvalues matrix. From an implementation point of view, solving the full problem only requires to construct a suitable matrix $\mathbf{M}$ based on the properties of the system of interestr, and generate a suitable starting condition for the experiment, from which we will compute $\mathbf{p}_0$. The orientational probability of the fluorophores at any time delays is then promptly computed with eq. S21.

The simulation of light-modulated experiments, in which laser powers are controlled with square waves, can be seen as a simple extension of the solution here proposed. The way to approach this issue it to divide the experiment in a series of time windows in which the laser powers are constant. Then for each time window a rate matrix $\mathbf{M}$ is constructed and used to propagate the probabilities. The first time window will propagate the initial conditions of the experiment, whereas the following time windows will propagate the ending conditions of the previous time window.

S2.6 High-NA photo-selection

The angular dependence of $k_{\epsilon\eta}$ is the way we can create a photo-selected state in which the excited molecules have a preferential orientation. If we shine light polarized along the $\hat{\mathbf{x}}$ direction of the laboratory fixed coordinate system, we can write the following expression for the kinetic constant in the low-NA limit [7]

$$\begin{aligned} k_{\epsilon\eta}(\theta,\phi) &= |\hat{\mathbf{r}}(\theta,\phi)\cdot\hat{\mathbf{x}}|^2\sigma_{\epsilon\eta}\rho \\ &= F_x\sigma_{\epsilon\eta}\rho \end{aligned} \tag{S23}$$

where $\hat{\mathbf{r}}$ is a unitary vector pointing in the direction of the absorption transition dipole moment, $\hat{\mathbf{x}}$ is the unitary vector pointing in the direction of the electric field of the electromagnetic radiation, F_x is the function encoding the pure angular dependence of the kinetic constant trough the scalar product of the two versors, $\sigma_{\epsilon\eta}$ is the cross-section for the transition $\eta \rightarrow \epsilon$ expressed in cm^2, and ρ is the photon flux expressed in $\text{cm}^{-2}\text{s}^{-1}$. Cross-section and photon flux can be also expressed in terms of other widely used quantities as [8]

$$\sigma_{\epsilon\eta} = \frac{2303}{N_A}\varepsilon, \qquad \text{and} \quad \rho = I\frac{\lambda}{hc}, \tag{S24}$$

where N_A is the number of Avogadro, ε is molar extinction coefficient expressed in $\mathrm{M}^{-1}\mathrm{cm}^{-1}$, I is the power density of the electromagnetic radiation expressed in $\mathrm{W/cm}^2$, λ is the wavelength, h is the Planck constant, and c is the speed of light.

When using high-NA objectives, eq. S23 must be corrected taking in consideration the polarization mixing introduced by the focusing of the light [7]. The correction is obtained by integrating all the contribution to the excitation coming from the rays inside the focusing cone of the objective. The angle of the cone of the rays, α_0, is obtained from the numerical aperture NA and the refractive index of the medium n, i.e. through the equation NA= $n \sin\alpha_0$. The corrected kinetic constant is expressed as

$$k_{\epsilon\eta}(\theta,\phi) = (K_a F_z + K_b F_y + K_c F_x)\,\sigma_{\epsilon\eta}\rho \tag{S25}$$

where we assumed $\hat{\mathbf{z}}$ as the propagation direction, $\hat{\mathbf{y}}$ as the perpendicular direction, and where K_a, K_b, and K_c are the normalized Axelrod coefficients [7], specified as

$$\begin{aligned} K_a &= 1/6\ (2 - 3\cos\alpha_0 + \cos^3\alpha_0)/(1-\cos\alpha_0) \\ K_b &= 1/24\ (1 - 3\cos\alpha_0 + 3\cos^2\alpha_0 - \cos^3\alpha_0)/(1-\cos\alpha_0) \\ K_c &= 1/8\ (5 - 3\cos\alpha_0 - \cos^2\alpha_0 - \cos^3\alpha_0)/(1-\cos\alpha_0). \end{aligned} \tag{S26}$$

The correction coefficients are normalized such that $K_a + K_b + K_c = 1$, and in the low-NA limits they tend to $K_c = 1$ and $K_a = K_b = 0$.

S2.7 High-NA polarized detection

The fluorescence signal emitted by the sample and recorded from a polarized detector is derived with a similar formalism to the high-NA photo-selection correction [7]. For example, if we are observing with two cross polarized detectors, measuring the polarization along $\hat{\mathbf{x}}$ and $\hat{\mathbf{y}}$, the fluorescence signals are computed as

$$\begin{aligned} I_x(t) &= A\ \Phi^{\epsilon}_{\text{fluo}} \int\int p_\epsilon(t,\theta,\phi)\ \ (K_a F_z + K_b F_y + K_c F_x)\sin\theta d\theta d\phi \\ I_y(t) &= A\ \Phi^{\epsilon}_{\text{fluo}} \int\int p_\epsilon(t,\theta,\phi)\ \ (K_a F_z + K_b F_x + K_c F_y)\sin\theta d\theta d\phi \end{aligned} \tag{S27}$$

where $p_\epsilon(t,\theta,\phi)$ is the orientational probability of the fluorescent excited state, A is a constant embedding all the experimental factors and the size of the sample, and $\Phi^{\epsilon}_{\text{fluo}}$ is the quantum yield of fluorescence of state ϵ. Note that the constants K_a, K_b, and K_c are related again to propagation direction, perpendicular direction and polarization direction respectively, as in eq. S26. We are also assuming that there is only one fluorescent state ϵ. These expressions can be easily generalized for multiple fluorescent states. The integral in equations S27 can be easily computed exploiting the SH expansion of $p_\epsilon(t,\theta,\phi)$.

S2.8 Four states model for negative rsFPs

Photo-physics of rsFPs can be modeled as a 4 states system, with a ground on-state, a ground off-state, and the correspondent first excited states. The on- and off-state absorb in a different spectral region and we can use two laser with different wavelength to promote the interconversion among the states. The on-off switching happens through an excited state reaction, usually a cis-trans isomerization of the chromophore. Further processes might happen in the ground state, and in fast time-scales several intermediates can be revealed [9, 10]. Usually, in green negative rsFPs the great change of spectroscopical properties happens a after a proton transfer in the ground state [11].

Using the scheme in Figure S2.8(a), we can specify all the kinetic constants using parameters that are commonly found in rsFP literature

$$\begin{aligned} k_{21} &= \sigma_{21}(\lambda)\rho(\lambda) \\ k_{12} &= 1/\tau_{\text{on}} \\ k_{32} &= (1/\tau_{\text{on}})\ \Phi_{\text{on}\to\text{off}} \\ k_{43} &= \sigma_{43}(\lambda)\rho(\lambda) \\ k_{34} &= 1/\tau_{\text{off}} \\ k_{14} &= (1/\tau_{\text{off}})\ \Phi_{\text{off}\to\text{on}} \end{aligned} \tag{S28}$$

where τ_{on} and τ_{off} are the lifetimes of the excited states, $\Phi_{\text{on}\to\text{off}}$ and $\Phi_{\text{off}\to\text{on}}$ are the quantum yield for the off- and on- switching processes respectively, and λ the wavelength of the laser used to promote the ground to excited state transition. We are assuming that the quantum yields do not depend on the wavelength of the exciting laser.

In order to implement the rsFP photo-physics in the full rotational diffusion and kinetics model, we need to compute the kinetic constants as in eq. S17, thus we associate a block matrix $\mathbf{K}^{\epsilon\eta}$ to each kinetic rate. The block matrix include the details regarding high-NA photoselection, polarization state of the light, and the kinetic rate itself. For transitions that do not depend on the orientation of the fluorophores the block is simplified, such that $\mathbf{K}^{\epsilon\eta} = \mathbf{1}k_{\epsilon\eta}$. The block structure of the full matrix $\mathbf{K}$ can then be further specified as

$$\mathbf{K} = \begin{bmatrix} \square & \mathbf{K}^{12} & & \mathbf{K}^{14} \\ \mathbf{K}^{21} & \square & & \\ & \mathbf{K}^{32} & \square & \mathbf{K}^{34} \\ & & \mathbf{K}^{43} & \square \end{bmatrix} = \begin{bmatrix} \square & \mathbf{1}k_{12} & & \mathbf{1}k_{14} \\ \mathbf{K}^{21} & \square & & \\ & \mathbf{1}k_{32} & \square & \mathbf{1}k_{34} \\ & & \mathbf{K}^{43} & \square \end{bmatrix}. \tag{S29}$$

The out of diagonal matrix blocks that are missing in this expression are zeros, or in other words there are not processes that interconvert the specific couple of species. The diagonal matrix blocks, that are represented with squares $\square$ for sake of simplicity of notation, are obtained from the out of diagonal ones as in eq. S8, e.g. $\mathbf{K}^{22} = -\mathbf{1}k_{12} - \mathbf{1}k32$. Note that when multiple laser excitation are used the same species might absorb at all wavelength, and thus the corresponding kinetic matrix block will be the sum of the contributions due to different

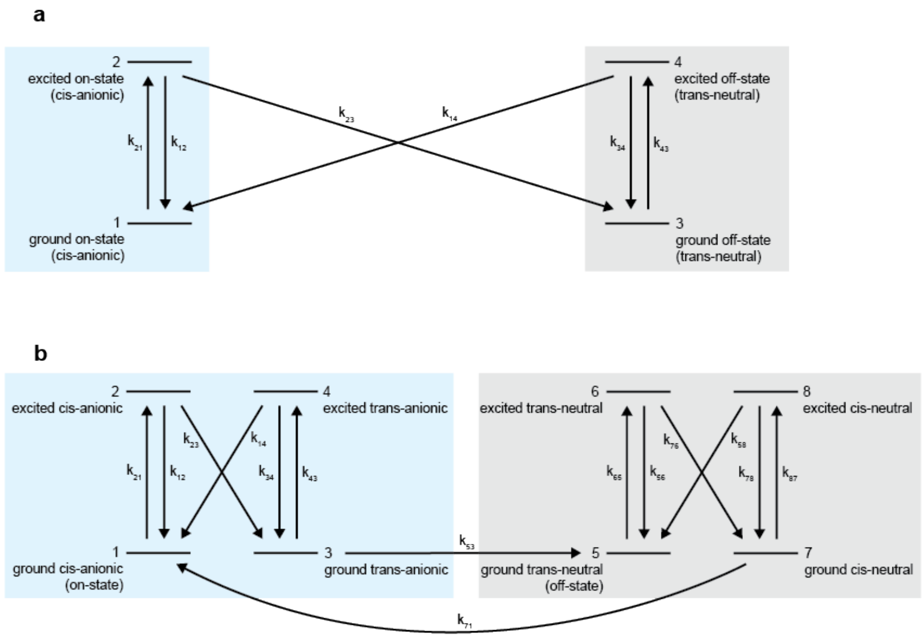

Figure S3: Kinetic models for negative rsFPs. (a) Four states model, and (b) eight states model. Light blue boxes highlight the anionic states, including the on-state, whereas gray boxes highlight the neutral states, which are all non-fluorescent. Fluorescence photons are emitted only from the state 2 (excited cis-anionic), when the pathway $1 \rightarrow 2$ is followed.

wavelengths, e.g. $\mathbf{K}^{21} = \mathbf{K}^{21}(405\text{nm}) + \mathbf{K}^{21}(488\text{nm})$. This is generally referred as crosstalk of the switching processes of rsFPs.

S2.9 Eight states model for negative rsFPs

When the power densities employed for the switching are high enough, the excited state cis-trans isomerization might not be the rate determining step anymore. In this condition we can have the accumulation of concentration of intermediates. For example starting from the ground on-state, after the excited state flipping from cis- to trans-conformers, a protonation must occur to reach the final ground off-state. This process usually take several μs or tens of μs, and it might involve several intermediates. The simplest way to include this process in the photo-physics is to use an 8 states model. Two intermediate species are inserted between on- and off- states. The resulting scheme is represented in Figure S2.8(b). For additional clarity, the states can be named after the conformation of the chromophore and the protonation state: cis or trans and anionic or neutral. The on-state is then also the cis-anionic state indexed with $\epsilon = 1$, and the off-state is also the trans-neutral state indexed with $\epsilon = 5$. The intermediate state during the off-switching process is the trans-anionic state indexed with $\epsilon = 3$, whereas the intermediate state during the on-switching process is the cis-neutral state indexed with $\epsilon = 7$. The excited states are respectively indexed with $\epsilon = 2, 4, 6, \text{and } 8$. We can specify all the kinetic constants involved in this model as

$$\begin{aligned}
k_{21} &= \sigma_{\text{on}}(\lambda)\rho(\lambda) \\
k_{12} &= 1/\tau_{\text{on}} \\
k_{32} &= (1/\tau_{\text{on}})\ \Phi^{\text{anionic}}_{\text{cis}\to\text{trans}} \\
k_{43} &= \sigma_{\text{on}}(\lambda)\rho(\lambda) \\
k_{34} &= 1/\tau_{\text{on}} \\
k_{14} &= (1/\tau_{\text{on}})\ \Phi^{\text{anionic}}_{\text{trans}\to\text{cis}} \\
k_{53} &= 1/\tau_{\text{prot}} \\
k_{65} &= \sigma_{\text{off}}(\lambda)\rho(\lambda) \\
k_{65} &= 1/\tau_{\text{off}} \\
k_{76} &= (1/\tau_{\text{off}})\ \Phi^{\text{neutral}}_{\text{trans}\to\text{cis}} \\
k_{87} &= \sigma_{\text{off}}(\lambda)\rho(\lambda) \\
k_{78} &= 1/\tau_{\text{off}} \\
k_{58} &= (1/\tau_{\text{off}})\ \Phi^{\text{neutral}}_{\text{cis}\to\text{trans}} \\
k_{17} &= 1/\tau_{\text{deprot}},
\end{aligned} \tag{S30}$$

where τ_{prot} and τ_{deprot} are the time constants for the ground state protonation and deprotonation processes. In this parametrization we are assuming that the absorption spectrum and the lifetimes of cis and trans species are the same.

This is a reasonable assumption in negative green rsFPs [12], but the model can easily accept different properties for cis and trans conformers.

The full kinetic matrix is constructed in analogy to eq. S29 as

$$\mathbf{K} = \begin{bmatrix} \Box & \mathbf{1}k_{12} & & \mathbf{1}k_{14} & & & \mathbf{1}k_{17} & \\ \mathbf{K}^{21} & \Box & & & & & & \\ & \mathbf{1}k_{32} & \Box & \mathbf{1}k_{34} & & & & \\ & & \mathbf{K}^{43} & \Box & & & & \\ & & \mathbf{1}k_{53} & & \Box & \mathbf{1}k_{56} & & \mathbf{1}k_{58} \\ & & & & \mathbf{K}_{65} & \Box & & \\ & & & & & \mathbf{1}k_{76} & \Box & \mathbf{1}k_{78} \\ & & & & & & \mathbf{K}_{87} & \Box \end{bmatrix}. \tag{S31}$$

Note that $\mathbf{K}^{21} = \mathbf{K}^{43}$ and $\mathbf{K}^{65} = \mathbf{K}^{87}$ because we are assuming the same absorption cross-sections for cis-anionic/trans-anionic states and trans-neutral/cis-neutral states respectively.

S2.10 Parametrization of rsEGFP2

The parametrization of the reversible switchable protein rsEGFP2 was mainly derived from [13]. In particular, the absorption properties of the on-state and off-state can be specified with the extinction coefficients [13],

$$\begin{aligned} \epsilon_{\text{on}}(488nm) &= 51560\ \text{M}^{-1}\text{cm}^{-1} \\ \epsilon_{\text{on}}(405nm) &= 5260\ \text{M}^{-1}\text{cm}^{-1} \\ \epsilon_{\text{off}}(488nm) &= 60\ \text{M}^{-1}\text{cm}^{-1} \\ \epsilon_{\text{off}}(405nm) &= 22000\ \text{M}^{-1}\text{cm}^{-1} \end{aligned} \tag{S32}$$

and then the cross-sections σ in cm^2 can be computed accordingly using the equation [8]

$$\sigma(\lambda) = \frac{2303}{N_A}\epsilon(\lambda), \tag{S33}$$

where N_A is the Avogadro number. The quantum yields of fluorescence and cis-trans isomerization are as follows [13]

$$\begin{aligned} \Phi_{\text{fluo}} &= 0.35 \\ \Phi^{\text{anionic}}_{\text{cis}\to\text{trans}} &= 1.65 \cdot 10^{-2} \\ \Phi^{\text{neutral}}_{\text{trans}\to\text{cis}} &= 0.33. \end{aligned} \tag{S34}$$

The fluorescence lifetimes are $\tau_{\text{on}} = 1.6$ ns [14] and $\tau_{\text{off}} \approx$ ps [10] (20 ps are assumed). Finally $\tau_{\text{deprot}} = 5.1$ us, in agreement to dynamics observed in transient absorption measurements [10], and $\tau_{\text{prot}} = 48\ \mu$s was determined from high-power off-switching data, see section S8. The absorption properties of cis and trans conformers of the same protonation state were taken as equal, i.e. $\epsilon_{\text{on}}(\lambda) = \epsilon^{\text{anionic}}_{\text{cis}}(\lambda) \approx \epsilon^{\text{anionic}}_{\text{trans}}(\lambda)$ and $\epsilon_{\text{off}}(\lambda) = \epsilon^{\text{neutral}}_{\text{trans}}(\lambda) \approx \epsilon^{\text{neutral}}_{\text{cis}}(\lambda)$. This is

a reasonable approximation given the fact that the absorption spectra are very close, see supporting information of [10].

Two remaining parameters are undefined, $\Phi^{\text{anionic}}_{\text{trans}\to\text{cis}}$ and $\Phi^{\text{neutral}}_{\text{cis}\to\text{trans}}$. These quantum yields enable the back-conversion from the intermediate states. $\Phi^{\text{anionic}}_{\text{trans}\to\text{cis}}$ will be fitted in section S7, whereas $\Phi^{\text{neutral}}_{\text{cis}\to\text{trans}}$ will be assumed as 0. Assuming a null quantum yield silences the associated process. This is a reasonable assumption if there is not an accumulation of intermediate in the on-switching process that is excited with 405 nm light. Further characterization will be needed to better characterize the back-conversion quantum yields.

S3 STARSS method 1 simulation of experiments

In STARSS method 1 experiments, a short pulse (about 250 ns) of polarized laser light at 405 nm is shined on a sample containing rsFPs. This induces the on-switching of a pool of fluorophores that are roughly oriented with the electric field of the light. The linear photo-selection process generates a "$\cos^2$" orientational probability distribution of on-switched molecules. Light-matter interaction limits the precision/sharpness of the orientational probability distribution that can be created. Additionally, high-NA objectives limit the degree of alignment of the on-switched pool of molecules because they introduce electric field components of the light in all dimensions, i.e. small components along Y and Z even if the direction of polarization of the beam is along X.

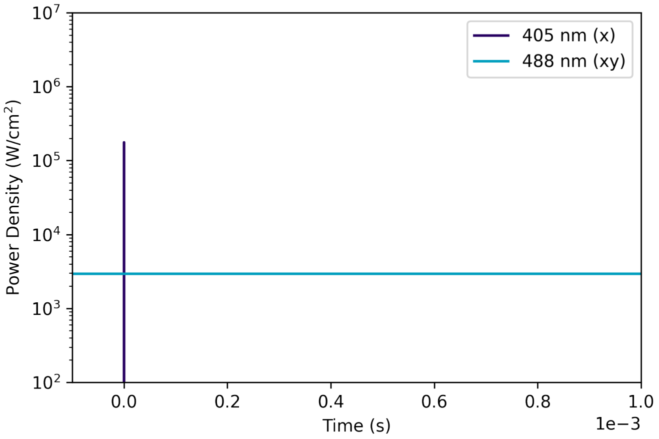

Figure S4: Power density of laser beams used in the simulation of STARSS with long-lived states method 1. The duration of the high power burst of 405 nm light is 250 ns. The circularly polarized 488 nm beam is kept on during the full duration of the pulse scheme.

At the beginning of the pulse scheme of this experiment the rsFPs are kept in the off-state by shining 488 nm light. The signal is not exactly zero because of the crosstalk between the on- and off-switching process, i.e. there is a small chance of on-switching the rsFPs using 488 nm light. After delivering the short high-power burst of 405 nm light, a fraction of rsFPs start the on-switching process. We can assume that the cis-trans conformational change, which is happening in the excited state, is instantaneous compared to the time-scale of the experiment. After the conformational change the rsFP chromophore is still not fluorescent, and a deprotonation in the ground state must happen in order to maturate the final fluorescent on-state (cis-anionic). The deprotonation process affects the data and generates an exponential rise of the fluorescence signal with a time scale of roughly 5 μs. For the simulation we used 5.1 μs which

is the time scale observed in transient spectroscopy experiments [10]. As soon as the proton transfer happens, there is a big shift of absorption properties of the rsFP chromophore. In the cis-anionic state there is a high probability of absorbing 488 nm photons, which can immediately trigger off-switching events. The competition of the maturation of the on-switching and the start of the off-switching generates fluorescence signals with a rise and then a decay, as shown in Figure S5. The mismatch between the detectors is induced by the on-switching photo-selection that lasts as long as the characteristic rotational diffusion time for the system. Note that repeating the same experiment with circularly polarized 405 nm light gives two identical signals in the cross-polarized detectors.

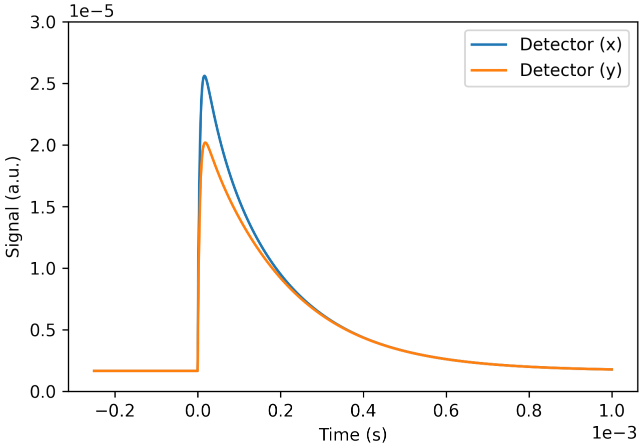

Figure S5: Detector signals for parallel (x) and perpendicular (y) channels in the simulation of STARSS with long lived states method 1. The rotational diffusion time in this simulated experiment is 100 μ and the fluorophore is rsEFGP2 with parameters presented in Section S2.10.

The on- and off-switching processes, inevitably coupled to the fluorescence readout of rsFPs, affect the experiments but do not sensibly modify the typical exponential decay observed in traditional time-resolved fluorescence anisotropy experiments. The simulated decay traces in Figure S6 (blue lines) are well approximated by a mono-exponential decay where the observed decay constant coincides with the rotational diffusion time (dashed orange lines). This suggests that we can borrow the traditional theory of time-resolved fluorescence anisotropy experiments [5] to extract the rotational diffusion coefficient and compute an estimate for the Stokes radius of the system labeled with rsFPs.

The main deviations from the mono-exponential behaviour are located at later times where slow rotational diffusive samples are faster than predicted with mono-exponential behavior. Fast rotational diffusive samples have a slight bi-exponential behavior with an additional small-amplitude fast component of

anisotropy. Nevertheless, there is a clear time window where blue and orange curves superimpose almost perfectly. When the off-switching process is almost completed (after a few hundreds of μs) the reactivation induced by circularly polarized 488 nm light adds a slow depolarization mechanism. This effect shortens the anisotropy decay when the rotational diffusion time is much slower than the off-switching time constant. In the simulated conditions and for the time window at 2-500 μs, the anisotropy traces are in good agreement with the mono-exponential signal expected from a standard time-resolved florescence anisotropy experiment. Note that slowing down the off-switching process by reducing the power density of the 488 nm light, can extend this window to much slower decays, at the cost of reducing the fluorescence counts.

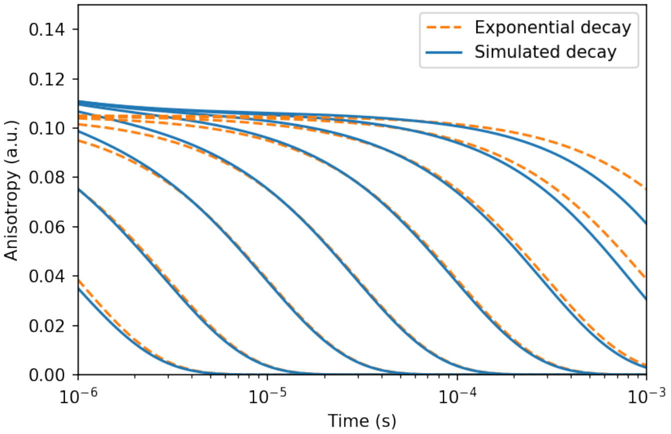

Figure S6: Fluorescence anisotropy decays computed for rsEFGP2 labeled systems in the simulation of STARSS with long lived states method 1 with rotational diffusion times of 1, 3, 10, 30, 100, 300, 1000, 3000 μs starting from left to right, respectively. Anisotropy is computed from similar curves to the ones presented in Figure S5, using the anisotropy equation and by subtracting the residual steady-state background obtained at infinity when the off-switching is considered completed.

The starting value r_0 of an anisotropy decay could theoretically start from a maximum value of 0.4, when linear photo-selection is involved. In a more realistic system several factors can diminish r_0. The rotation of the emission transition dipole moment with respect to the absorption transition dipole moment is a source of anisotropy loss. In green rsFPs, where a cis-trans isomerization is involved, the angle between the absorption transition dipole moment of the off-species and the emission transition dipole moment of the on-species is about $\alpha = 30$-$35°$ [15]. This induces a reduction of the maximum anisotropy to $r_0 = (3\cos^2\alpha - 1)/5 \approx 0.32$-$0.29$ [5]. Additional losses of anisotropy arise from: (i) saturation of photo-selected states, that happens when the on-switched

fraction is much higher than 10% (this could be used to increase the fluorescence photon counts); (ii) high-NA photo-selection and detection that mixes the polarization of the excitation light and the detection collection efficiency, thus reducing the maximum difference that can be measured between the detectors; and (iii) fast local mobility of the probe, that introduces anisotropy decay components that are lost in sub-microsecond timescales. The simulation proposed here includes all the effects that couple on- and off-switching processes to the losses of anisotropy, and also high-NA effects. Rotation of the transition dipole moments and a modelling of diffusion at fast local mobility are not included, but they will be the subjects of future studies.

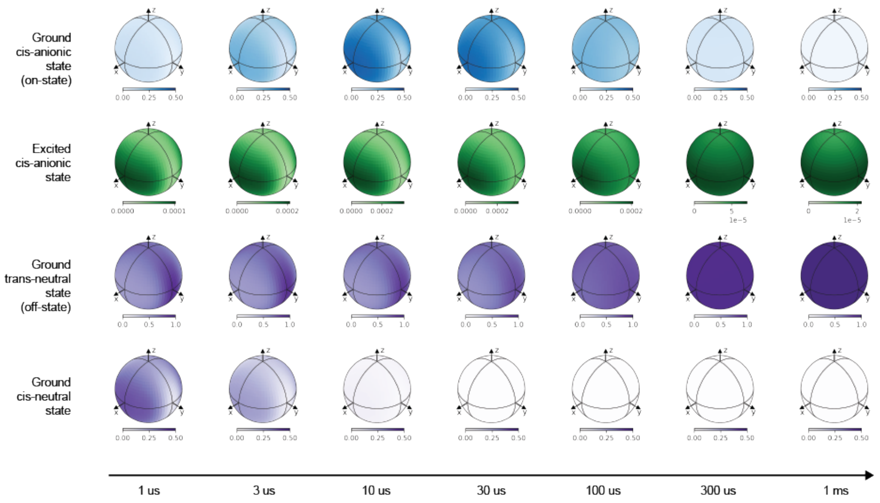

Figure S7: Time evolution of orientational probabilities of ground cis-anionic state (blue), excited cis-anionic state (green), ground trans-neutral state (purple, top), and ground cis-neutral state (purple, bottom) for STARSS method 1. The rotational diffusion time in this simulated experiment is 100 μs and the fluorophore is rsEFGP2.

The experiment can be followed best by exploring the time evolution of the orientational probabilities of the states involved in the kinetics of on- and off-switching. At the beginning of the experiment the system is prepared in a way such that almost all the fluorophores are in the off-state. After on-switching with a 405 nm laser pulse, a fraction of the fluorophores with the transition dipole moment aligned along X is converted from the trans-neutral to the cis-neutral state. In a few microseconds, the cis-neutral chromophores convert spontaneously to the cis-anionic (on-) state. This process generates a rise in the fluorescence signal, that is polarized if the rotational diffusion of the fluorescently labeled system is slow enough. If slow rotational diffusion is present the excited cis-anionic state population distribution resembles the one of the photo-selected state, i.e. the green distribution in figure S7 resemble the purple

distribution in the last row at early times. As the rotational diffusion evolves, the excited cis-anionic state population loses the polarized shape and becomes symmetric along x and y. At the same time the off-switching process, which for negative switchers is coupled to the readout, decreases the total population of the cis-anionic state, reaching again the initial condition of the experiment.

S4 STARSS method 1 size of silica beads

Sample	τ_R (μs)	r_0	r_∞	d (nm)
30 nm	5.5 (1.9)	0.051 (0.019)	0.0014 (0.0005)	35.0 (4.0)
50 nm	11.2 (1.7)	0.072 (0.010)	0.0020 (0.0006)	44.1 (2.2)
100 nm	105 (10)	0.087 (0.004)	0.0057 (0.0020)	93.2 (3.0)

Table S2: Silica beads mono-exponential fitting of data reported in Figure 3c of the main manuscript. Parameters and standard errors between brackets (expressed as square root of the estimated variance from the Jacobian of the residuals of the fit). The model function is reported in eq. S5 of Chapter S1. The calculations of the sizes was performed with eq. S3, using 1.0049 cP as viscosity of aqueous environment at 20°C. Power densities and pulse scheme are reported in Table S7 of Chapter S19.

S5 STARSS method 1 and anisotropy variance

rsEGFP and Dronpa variants display a highly efficient switching by 405 nm light, mainly inducing a cis-trans conformational change of the chromophore in the excited state or excited-state proton transfer[16, 17]. However, fluorescence emission also relies on other non-light-driven ground-state molecular transitions, such as spontaneous deprotonation of the chromophore and structural relaxations, which introduce further delays. The time constant of the rise of the fluorescence signal is an intrinsic property of the label (about 5 µs for DronpaM159T and rsEGFP2), and it sets an experimental trade-off between speed, i.e., smaller measurable molecular size, and photon budget. In fact, at times shorter than 5 µs only a few photo-selected rsFPs are able to emit fluorescence. As shown in Section S3, signals from STARSS with rsFPs modality 1 can be interpreted with the theory of traditional time-resolved fluorescence anisotropy. The major difference is the rise of the fluorescence signal in the initial transient. This happens because at the beginning of the experiment most of the molecules are in the off-state, and after the on-switching laser pulse the maturation of the fluorescent state will take a few microseconds. This time is required for the completion of the deprotonation process of the fluorophores in the ground state.

We can approximate the intensities of the detectors modifying the standard expression for fluorescence anisotropy decays [5], by adding crosstalk background and a rise at early times. The expected values in the detectors are approximated as

$$\begin{aligned} E(I_{\parallel}) &= b_{\parallel} + I_0 \left(1 - e^{-\frac{t}{\tau_H}}\right) e^{-\frac{t}{\tau_{\text{off}}}} \left(1 + 2r_0 e^{-\frac{t}{\tau_R}}\right) \\ E(I_{\perp}) &= b_{\perp} + I_0 \left(1 - e^{-\frac{t}{\tau_H}}\right) e^{-\frac{t}{\tau_{\text{off}}}} \left(1 - r_0 e^{-\frac{t}{\tau_R}}\right) \end{aligned} \quad \text{(S35)}$$

where I_0 are the counts that would be measured by a detector with polarization at the magic angle, τ_H is the deprotonation time constant, τ_{off} is off-switching time constant, r_0 is the anisotropy value at time equal zero, and τ_R is the rotational diffusion time. The expressions in eq. S35 represent a fluorescence signal that rises and decays over time as a result of the switching of the rsFPs, including a polarization term that affects the two channels as it would in a standard fluorescent anisotropy experiment.

Figures S8 to S11 reports theoretical signals computed for several rotational diffusion times and experimentally reasonable parameters. In these figures the background levels of the two channels are equal, $b_{\parallel} = b_{\perp} = I_{bg}$, and the confidence interval of the anisotropy value is computed with eq. S43. A common feature to note is that the confidence intervals decrease as the fluorescence signal increases. This behaviour imposes limits on the fastest rotational diffusion process that can be measured, given a certain signal to noise ratio and deprotonation time. In other words, higher signals and faster on-switching allow measuring faster rotational diffusion processes.

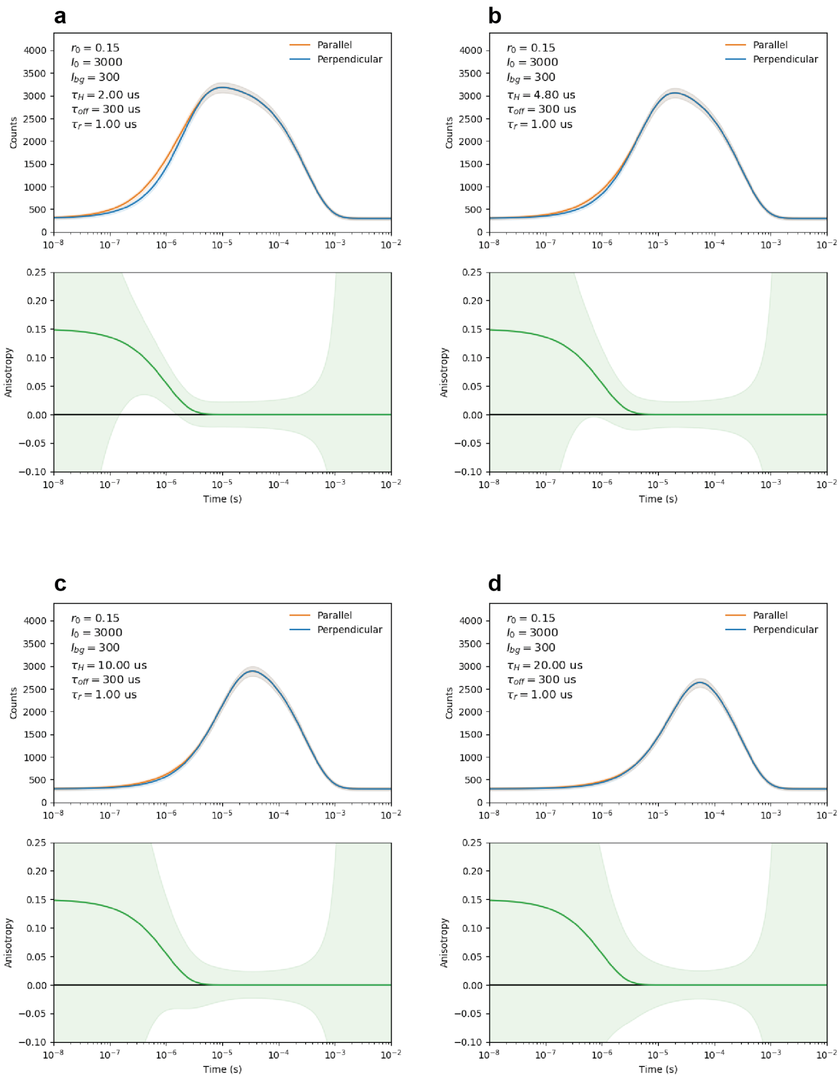

Figure S8: Examples of simulated STARSS method 1 signals using the simplified eq. S35. From (a) to (d) all the parameters are kept constant except t_H. In the sub-panels the top part shows the expectation values of the signals in the two detector channels, whereas the bottom sub-panel shows the anisotropy and confidence intervals computed with eq. S40 and S43. A shorter t_H allows to be more sensitive to fast rotational diffusion processes. For the examples in the figure, the anisotropy curve is significantly above the zero line only in panel (a) with the shortest t_H.

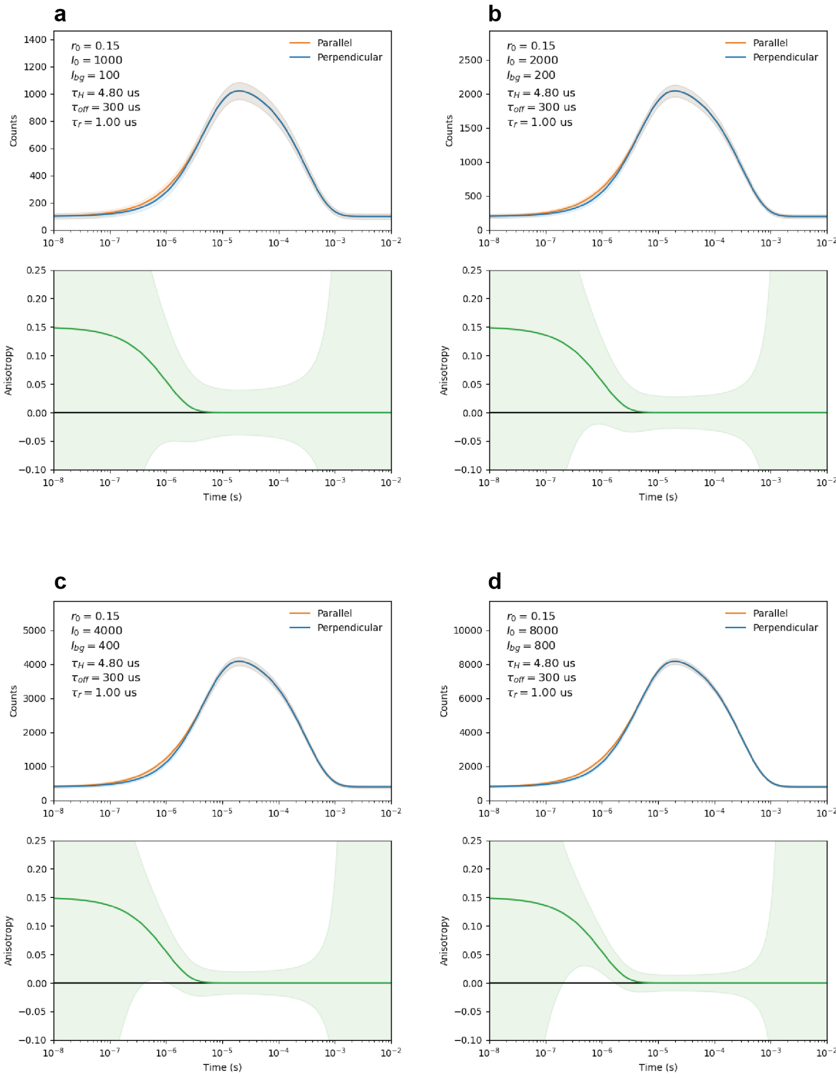

Figure S9: Examples of simulated STARSS method 1 signals using the simplified eq. S35. From (a) to (d) all the parameters are kept constant except I_0 and I_{bg}. In the sub-panels the top part shows the expectation values of the signals in the two detector channels, whereas the bottom sub-panel shows the anisotropy and confidence intervals computed with eq. S40 and S43. These examples emulate the increase in the total counts from an experiment, e.g. by increasing the exposure time or averaging of multiple signals. With more counts the variance reduces and the time window where the measured anisotropy can be distinguished from zero values of anisotropy widens.

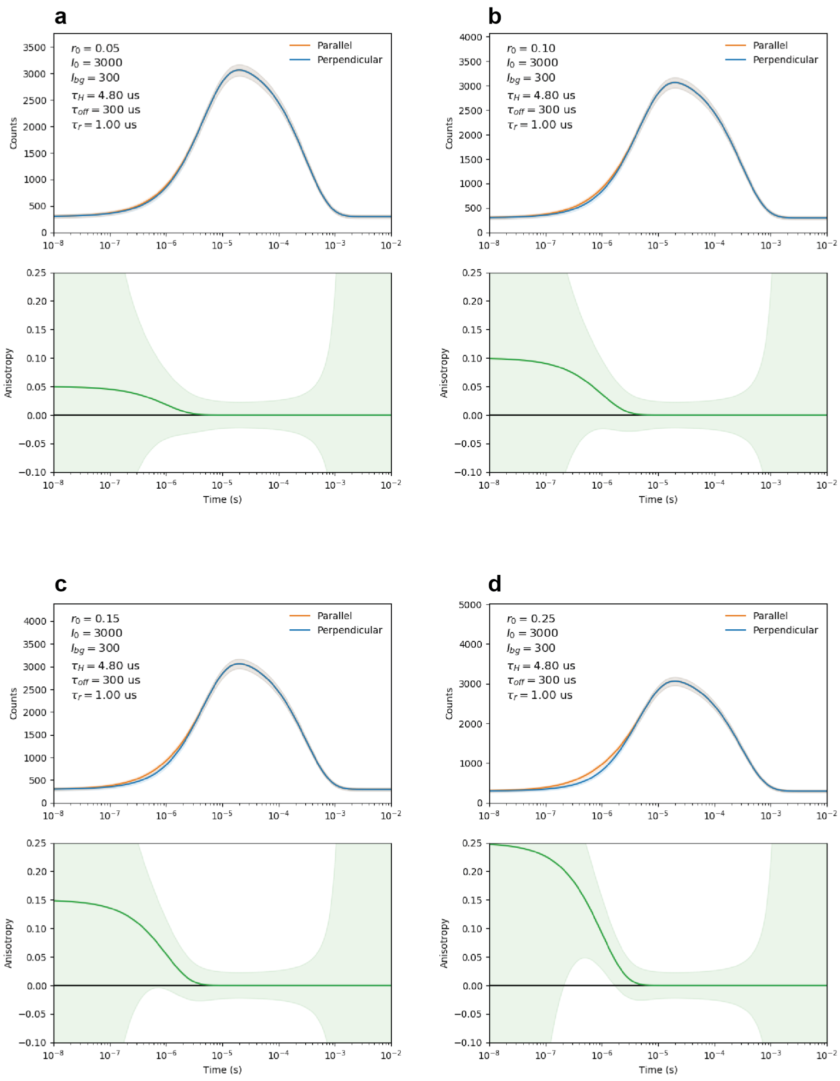

Figure S10: Examples of simulated STARSS method 1 signals using the simplified eq. S35. From (a) to (d) all the parameters are kept constant except r_0. In the sub-panels the top part shows the expectation values of the signals in the two detector channels, whereas the bottom sub-panel shows the anisotropy and confidence intervals computed with eq. S40 and S43. The bigger the starting anisotropy value and the easiest is to identify very fast rotational diffusion components.

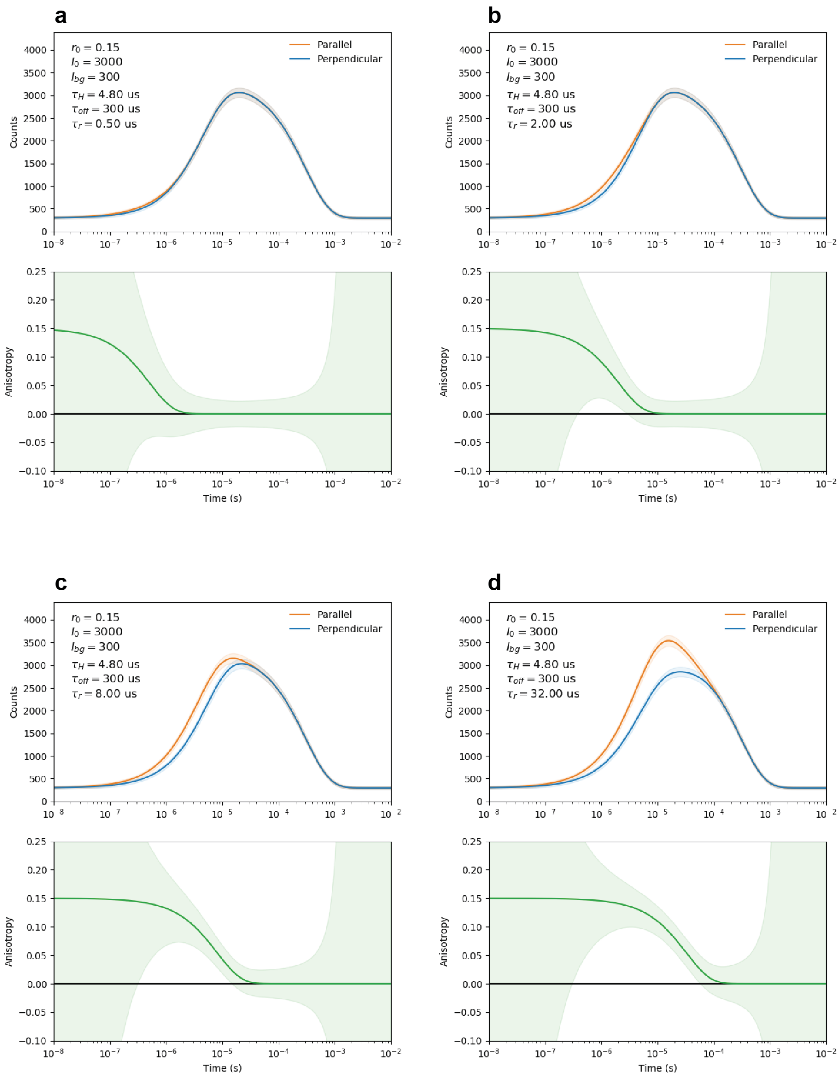

Figure S11: Examples of simulated STARSS method 1 signals using the simplified eq. S35. From (a) to (d) all the parameters are kept constant except τ_R. In the sub-panels the top part shows the expectation values of the signals in the two detector channels, whereas the bottom sub-panel shows the anisotropy and confidence intervals computed with eq. S40 and S43. Given a certain signal to noise ratio, there is a time window where the anisotropy has a variance small enough to be distinguished from zero. Processes with rotational diffusion time that are too fast might be not detectable.

S6 STARSS method 2 simulation of experiments

In STARSS with long-lived states methods 2, circularly polarized 405 nm laser light is used to activate a pool of molecules without preferential orientation along X or Y directions. A waiting time of 500 μs is used to let all the on-switching processes to be completed. The length of the waiting time also allows for the completion of slow relaxations of the fluorophore, which have been observed in transient absorption experiments [9, 10]. After on-switching, an X-linearly polarized 488 nm pulse is used to preferentially off-switch the molecules aligned with the electric field. Rotational diffusion will remove off-switched rsFPs from the X orientation, and it will repopulate the same orientation with on-switched rsFPs.

The competition of the diffusion and the off-switching processes strongly influences the fluorescence signals measured in the two detector channels. In particular, if rotational diffusion is the fast dominant dynamics, the anisotropy will stay high and constant during the full length of the experiment. In contrast, if the off-switching kinetics is competitive or faster than rotational diffusion, there will be a decay of the fluorescence anisotropy signal. The loss of anisotropy is due to a completely different phenomenon than in STARSS method 1, and negative values are also possible. This decay is due to a loss of on-switched fluorophores aligned along the parallel detection channel. With slow rotational diffusion these molecules are not exchanged for fresh ones, and a loss of signal in the parallel channel builds up. In the limit of extremely slow rotational diffusion, there could be an almost complete off-switching along the direction of the parallel channel, and negative anisotropy values are obtained.

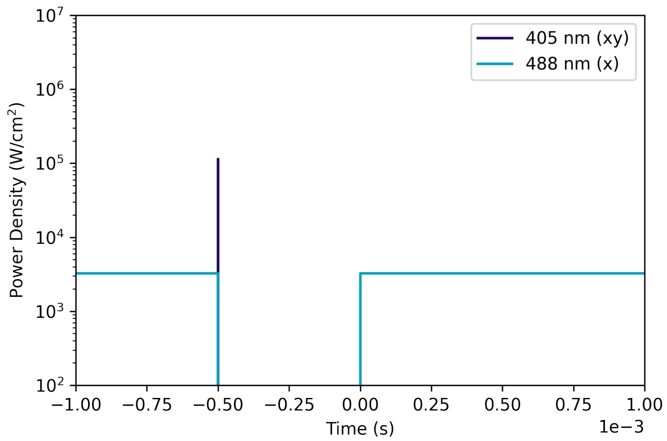

Figure S12: Power density of laser beams used in the simulation of STARSS with long-lived states method 2. During the waiting time of 500 μs, we are not shining light on the sample, letting the on-switching process fully complete.

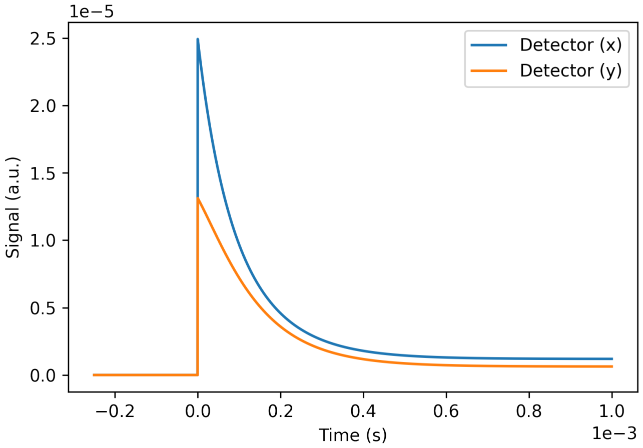

Figure S13: Detector signals for parallel (x) and perpendicular (y) channels in the simulation of STARSS with long-lived states method 2 for a rotational diffusion time of 100 μs and rsEFGP2 fluorophore.

The decay of fluorescence anisotropy observed in STARSS method 2 is not easily related to a numerical value of the rotational diffusion coefficient of the system, mainly because the resulting signal is intertwined with photo-physics of the probe. Thus, in order to retrieve the rotational diffusion coefficients, a simulation that includes photo-physical processes must be used. This require more complex modeling compared to STARSS method 1 where the analogy with the traditional time-resolved fluorescence anisotropy experiment allows for borrowing the theoretical background. STARSS method 2 experiments have a more convenient on-switching step, that allows saturation of the on-switched population without degradation of the anisotropy signal. This implies that more fluorophores can be on-switched at the same time and higher fluorescence counts can be obtained. Care must be taken if bleaching becomes a relevant factor.

The orientational populations in figure S15 complement the description of the experiment with an example for a rotational diffusion time of 100 μs. At early times a fraction of fluorophores is in the on-state without any preferential orientation, i.e. the first blue sphere is uniformly colored with the shade corresponding to the starting population of on-switched fluorophores. The excited cis-anionic state, represented in green, exhibits a "$\cos^2$" photo-selection at the beginning of the experiment. This happens because the fluorescence is excited using 488 nm linearly polarized light. The off-switching of fluorophores is more probable when molecules are aligned along X, thus a population *hole* builds-up along X, and the fluorescent photo-selected state will gradually change shape allowing more and more counts to reach the perpendicular detector. At the end of the experiment the system resets itself and is ready for the next pulse sequence.

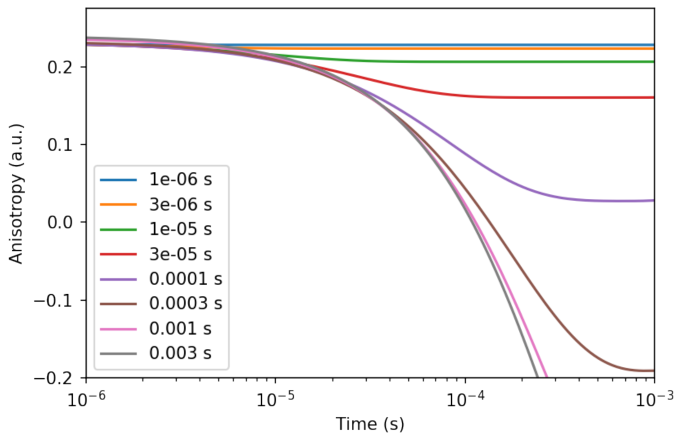

Figure S14: Fluorescence anisotropy decays computed for rsEFGP2-labeled systems for STARSS with long-lived states method 2 with rotational diffusion times of 1, 3, 10, 30, 100, 300, 1000, 3000 μs.

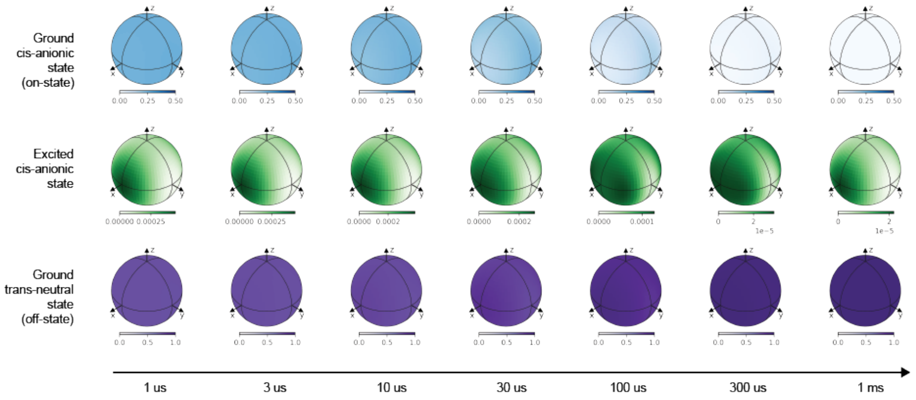

Figure S15: STARSS with long-lived states method 2. Time evolution of orientational probabilities for the ground cis-anionic state (blue), excited cis-anionic state (green), and ground trans-neutral state (purple) for a rotational diffusion time of 100 μs with rsEGFP2 as fluorophore.

S7 STARSS method 2 fit of silica beads data

The theoretical modelling is based on the analytical solution of the diffusion problem coupled with the kinetics of photo-switching, relying on the expansion with spherical harmonics [2] of the orientational probabilities of rsFP states. Data from STARSS method 2 were fitted using the model presented in Section S2. The 8-states model of rsEGFP2 was used as parametrized in Section S2.10. The experiment was simplified assuming that, at the beginning of the experiment, 100% of the population was localized on the cis-anionic (on-) state. The signal is then computed as the off-switching process is happening, stimulated by linearly polarized 488 nm excitation.

The first step was to fit experimental data from rsEGFP2 in solution recorded with STARSS method 2. The rotational correlation time of GFP is known, $\tau_R =$ 16.5 ns [18]. The same τ_R is assumed for rsEGFP2. This first fitting was used as a calibration for a few experimental parameters. In particular, two parameters were fitted: (i) the effective numerical aperture of the system $\mathrm{NA}_{\mathrm{eff}}$, and (ii) a correction factor I_{corr} for the power density measured experimentally at the back aperture. This experiment was performed at a lower power in order to reduce all the effects induced by the intermediate trans-anionic state, i.e. in order to have an off-switching profile closer to a mono-exponential. The effective power density for 488 nm light plugged in the model was defined as $I_{488}^{\mathrm{eff}} = I_{488}^{\mathrm{exp}} I_{\mathrm{corr}}$ 86%, where I_{488}^{exp} is the experimental power density and 86% is the transmittance of the objective (Leica, STED White, 100x, NA=1.4, n=1.516).

The second step was to fit experimental data from 500 nm silica beads labeled with rsEGFP2. These beads precipitate on the coverglass and thus were rigidly anchored on the surface. The experiment was performed with a higher power than the experiment on rsEGFP2 in solution, the same power was used in all the following experiments. The aim was to accelerate the off-switching process, such that it had a better chance to compete with the rotational diffusion in the smaller beads. Two parameters were fitted: (i) the apparent rotational diffusion time τ_R, and (ii) the quantum yield for the excited state conformational change from trans-anionic state back to cis-anionic state $\Phi_{\mathrm{trans}\to\mathrm{cis}}^{\mathrm{anionic}}$. The process that brings back the cis-conformer from the trans- is relevant only at high power densities when the rate determining step is not the light-driven process, but the protonation step in the ground state. In other word, the process is relevant if there is an accumulation of trans-anionic population that can absorb 488 nm light and inter-convert back to the starting condition.

Finally, the data from the library of freely diffusing beads (30, 50, 100 nm) were fitted. All the parameters of the model were kept fixed and only the effective rotational diffusion time was optimized. The full model reported in section S2 considers only one diffusive process, with one characteristic time constant. In a complex system as the studied one, it is a reasonable hypothesis to expect multiple rotational diffusion processes at several time scales. For example, rsEGFP2 probes might have a fast but very constrained wobble in the nanoseconds time scale and a much slower global tumbling of the full bead system in the microseconds time scale. The time constant extracted with the fitting takes

into account in an averaged way of all the rotational diffusive processes, thus it is reasonable to assume that it is faster than the real global tumbling. The global tumbling is by definition the slower rotational time constant of the system. Indeed the rotational diffusive constant extracted are all faster than what obtained from STARSS method 1, in Table S6, where it is easier to separate the slowest process as the last anisotropy decay component.

In table S3 are reported all the parameters of the model used in the fitting. Experimental data and fitted models are reported in figures S16, S17, S18, S19, and S20.

Parameter	rsEGFP2 (sol)	500 nm	30 nm	50 nm	100 nm
NA_{eff}	**1.1077**	1.105	1.105	1.105	1.105
n	1.516	1.516	1.516	1.516	1.516
I_{488}^{exp} (kW/cm^2)	6.58	19.6	19.6	19.6	19.6
I_{corr}	**0.326**	0.395	0.395	0.395	0.395
$\Phi_{trans \to cis}^{anionic}$	0	**0.0213**	0.0227	0.0227	0.0227
$\Phi_{cis \to trans}^{netural}$	0	0	0	0	0
τ_R (s)	$20 \cdot 10^{-9}$	$\mathbf{402 \cdot 10^{-6}}$	$\mathbf{2.98 \cdot 10^{-6}}$	$\mathbf{6.92 \cdot 10^{-6}}$	$\mathbf{96.3 \cdot 10^{-6}}$
d^{eff} (nm)	-	-	28.4	37.6	90.4

Table S3: Parameters of the model used to fit the experimental data recorded for the library of silica beads functionalized with rsEGFP2. The full set of parameters of rsEGFP2 include the parametrization provided in Section S2.10. Parameters in bold were optimized by the fitting routine, all the other parameters were kept fixed. The effective size d^{eff} is computed from the effective rotational diffusion time with equation eq. S3.

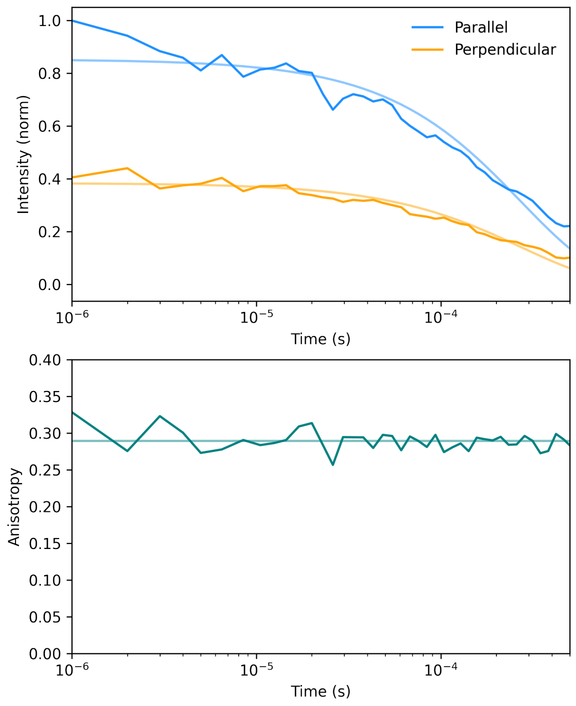

Figure S16: Experimental fluorescence anisotropy data recorded with STARSS method 2 for rsEGFP2 in solution. Normalized fluorescence intensity for parallel and perpendicular counts (top), and fluorescence anisotropy computed with eq. S40 (bottom). Lines with lighter colors show the observables predicted by the fitted theoretical model.

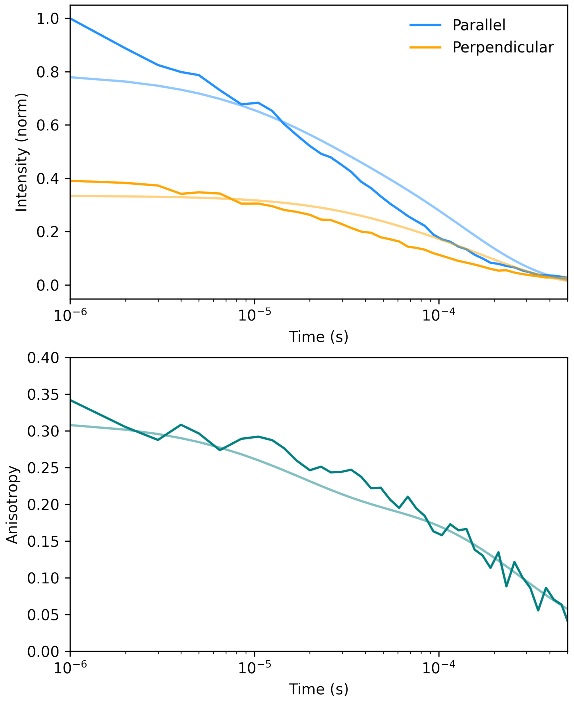

Figure S17: Experimental fluorescence anisotropy data recorded with STARSS method 2 for 500 nm silica beads functionalized with rsEGFP2. Normalized fluorescence intensity for parallel and perpendicular counts (top), and fluorescence anisotropy computed with eq. S40 (bottom). Lines with lighter colors show the observables predicted by the fitted theoretical model.

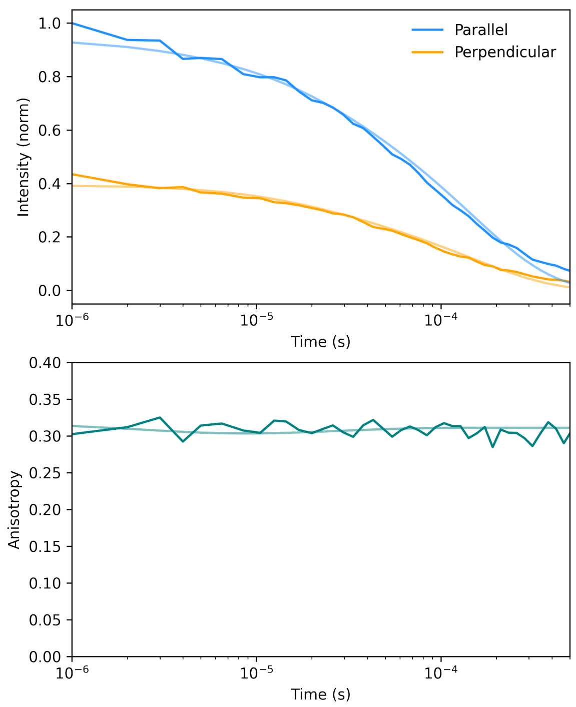

Figure S18: Experimental fluorescence anisotropy data recorded with STARSS method 2 for 30 nm silica beads functionalized with rsEGFP2. Normalized fluorescence intensity for parallel and perpendicular counts (top), and fluorescence anisotropy computed with eq. S40 (bottom). Lines with lighter colors show the observables predicted by the fitted theoretical model.

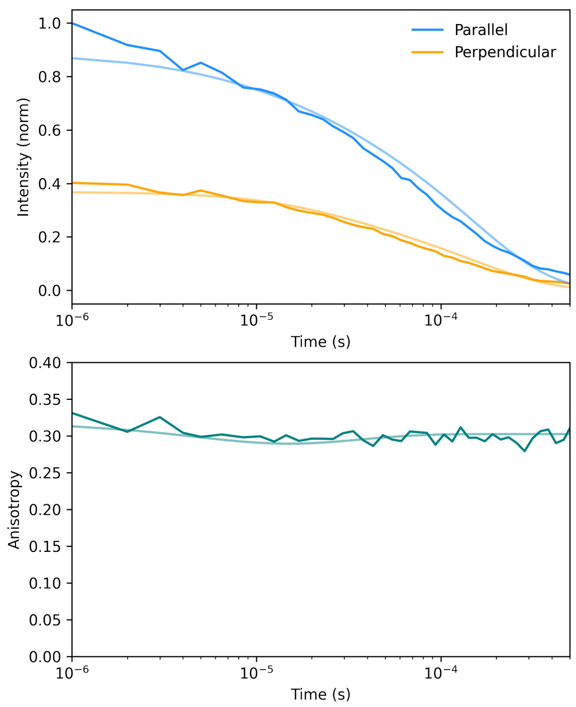

Figure S19: Experimental fluorescence anisotropy data recorded with STARSS method 2 for 50 nm silica beads functionalized with rsEGFP2. Normalized fluorescence intensity for parallel and perpendicular counts (top), and fluorescence anisotropy computed with eq. S40 (bottom). Lines with lighter colors show the observables predicted by the fitted theoretical model.

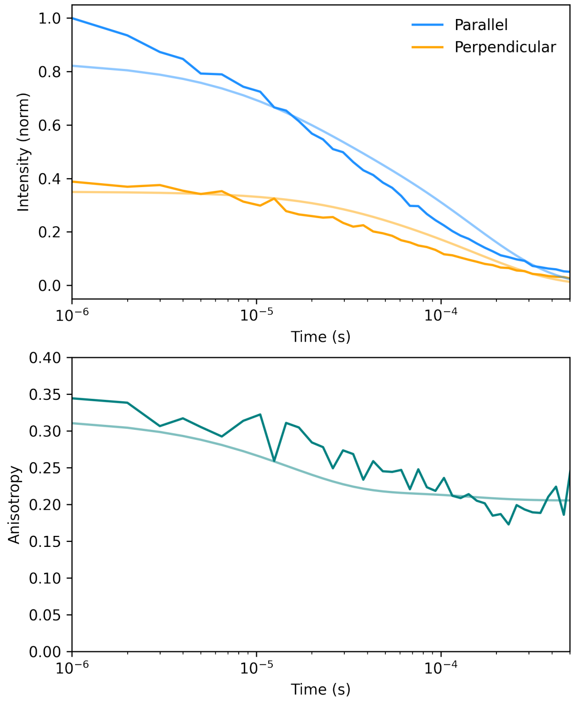

Figure S20: Experimental fluorescence anisotropy data recorded with STARSS method 2 for 100 nm silica beads functionalized with rsEGFP2. Normalized fluorescence intensity for parallel and perpendicular counts (top), and fluorescence anisotropy computed with eq. S40 (bottom). Lines with lighter colors show the observables predicted by the fitted theoretical model.

S8 High-power OFF-switching photo-physics of rsEGFP2

The 8-states model for the switching of rsFPs presented in section S2 implies that there are two main steps in order to switch between long-lived states. The first step is induced by light-absorption, and thus the corresponding time constant can be modulated by changing the power density of the laser light used for switching. The second step is happening in the ground state (protonation/derpotonation for rsEGFP2 and DronpaM159T) and the corresponding time constant is an intrinsic property of the system. At low power densities ($<$ KW/cm^2), the slowest process is the light-induced conformational change for most green negative rsFPs. In this condition, a mono-exponential behaviour can be observed in the fluorescence off-switching curves, and the protonation kinetics does not affect the observed decay. At high power densities, where the protonation is the rate determining step, a clear bi- or multi-exponential behaviour emerges in the off-switching decays. Since the protonation rate is not affected by light-absorption, it's rate is not affected by the power density of the laser.

In Figure S21, we reported experimental off-switching decays of rsEGFP2 recorded with the confocal system used for STARSS measurments. Switching was performed with circular 488 nm light. The on-switched population was prepared shining a 5 μs pulse of circularly polarized 405 nm light at 4.45 KW/cm^2, and then waiting for 100 μs. Each pulse scheme was repeated 500 times for 25 spots of the fluorescent gel sample. The background counts induced by 488 nm were removed from the decays. This was measured as the fluorescence intensity when steady state condition was reached at the end of the off-switching process.

The data in Figure S21 were fitted using a tri-exponential model. The fitting was constrained keeping one of the decay times constant at 48 μs. This time was extracted from the unconstrained fitting of the decay at the highest power-density. The lowest power-density curve collapsed to a bi-exponential model, i.e. the 48 μs component is automatically rejected by the optimization. The need of at least two exponential components is motivated by the fact of performing the experiment in confocal settings, where the power density is not spatially constant across the point-spread-function of the system. Nevertheless at high power densities the bi-exponential model is not capable of fitting the data. This is due to ground state processes that lag behind the light-induced conformational change. Decay times and amplitudes are reported in Table S4. Overall, the fitted model supports the hypothesis of an intermediate ground state process with a time constant of roughly 48 μs in rsEGFP2. Further analysis using the full kinetic will be performed in future studies.

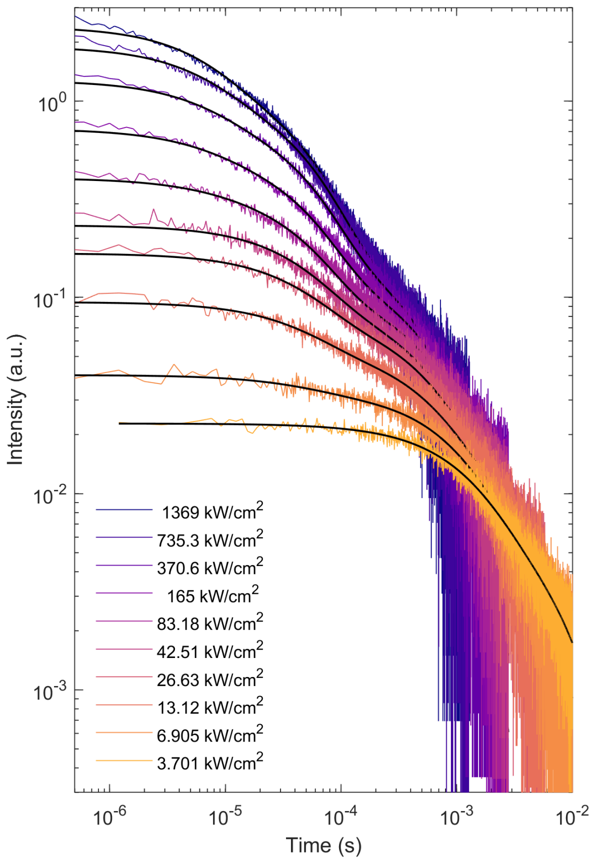

Figure S21: Fluorescence decay traces recorded from a poly-acrylamide embedded rsEGFP2 in Tris-NaCl (pH 7.5). The experiment was repeated at several power densities of 488 nm light. Power densities were computed as the power at the back aperture of the objective divided by the surface of a circular target with a diameter equal to the FWHM of the spatial point-spread-function.

Table S4: Fitted parameters and standard error as standard deviation for the tri-exponential fit reported in Figure S21. The equation for the fit is $y(t) = a_1 \exp(-t/\tau_1) + a_2 \exp(-t/\tau_2) + a_3 \exp(-t/48\text{us})$. Amplitudes are reported normalized, i.e. $a_1 + a_2 + a_3 = 1$.

Power Density (KW/cm^2)	a_1	s.d.	a_2	s.d.	a_3	s.d.	τ_1 (us)	s.d. (us)	τ_2 (us)	s.d. (us)
1369	0.484	0.004	0.086	0.001	0.430	0.004	7.2	0.1	410	5
735	0.419	0.004	0.108	0.001	0.473	0.004	7.0	0.1	431	4
371	0.341	0.004	0.137	0.001	0.522	0.004	7.0	0.2	494	3
165	0.240	0.005	0.184	0.001	0.575	0.005	6.9	0.3	621	3
83.2	0.117	0.007	0.257	0.001	0.626	0.006	6.7	0.8	715	3
42.5	0.228	0.005	0.186	0.008	0.586	0.004	365	16	1459	40
26.6	0.297	0.003	0.180	0.003	0.524	0.003	448	8	2021	24
13.1	0.312	0.004	0.280	0.005	0.407	0.005	458	12	2114	24
6.9	0.523	0.005	0.306	0.006	0.172	0.008	869	15	3836	54
3.7	0.664	0.008	0.334	0.009	0.002	0.012	1253	24	6750	172

S9 STARSS method 3 simulation of experiments

In STARSS method 3 an ensemble of rsFPs is on-switched with a couple of very short (50 ns) high power density 405 nm linearly polarized laser pulses. After a waiting time of 200 μs, the ensemble of on-switched fluorophores is readout with 488 nm circularly polarized light, on a detector without polarization analyzer. The power density of 405 nm laser pulses is tuned to induce some degree of saturation of the on-switching transition. In this way, when the second pulse arrives at the sample, there will be a different yield of the on-switching process depending on if the fluorophores are still aligned or if they diffused rotationally. Thus, changing the delay between the pulse pair generates a signal that depends on the rotational diffusion coefficient.

The combined fluorescence signal acquired after a pair of on-switching pulses is normalized by dividing by the fluorescence signal obtained from a single on-switching pulse. In this way, the normalized signal ranges between 1 and 2. If the first pulse fully saturates the on-switching process and all the fluorophores are activated regardless of their orientation the signal will be close to 1. In the other limit, when the on-switching is purely linear, the signal will be close to 2, i.e. each 405 nm pulse is on-switching an equal small amount of fluorophores. For this modulation to be representative of the rotational diffusion it is important to induce ON-switching away from the linear regime but also before saturation. If the ON-switching is driven in the linear regime a distinct population of molecules are independently and additively photo-switched into the ON-state. On the other hand, if we saturate the transition by switching all the rsFPs into the ON-state with the first pulse no modulation can be detected. To obtain the best modulation as a function of the rotational diffusion, the level of saturation should be intermediate and a signal around 1.5 is desirable. When the normalized signal is close to 1 or 2 the rotational diffusion has a small effect.

This experiment has very low hardware requirements on the detection, i.e. there is no need of polarization-sensitive and time-resolved detectors. During the readout with 488 nm light, the fluorescence signal is expected to decay because of the off-switching process. The final observable is the integral of the total counts of the detector during the off-switching curve. An example of off-switching decay after the double pulse activation is reported in Figure S23. STARSS method 3 signals fully neglect the time information of the fluorescence observable during the off-switching process. For this reason it is not necessary to study the evolution of the orientational population along the full pulse scheme.

Repeating the pulse scheme in Figure S22 for several delay times generated values plotted in Figure S24. An increase of the total fluorescence is observed when the delay between the pulses is increased. Experimentally a bigger dynamic range for STARSS method 3 experiments was observed, with signals ranging from 1.5 to about 1.7. Nevertheless the shape of the simulated signal capture the same trends observed in experiments (see Figure 2i of main manuscript).

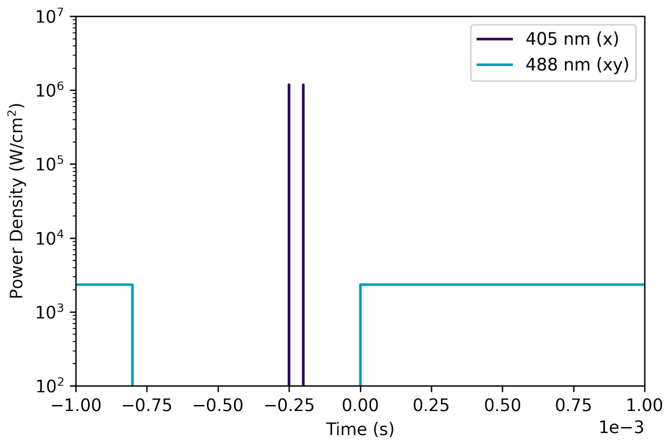

Figure S22: Power density of laser beams used in the simulation of STARSS with long-lived states method 3.

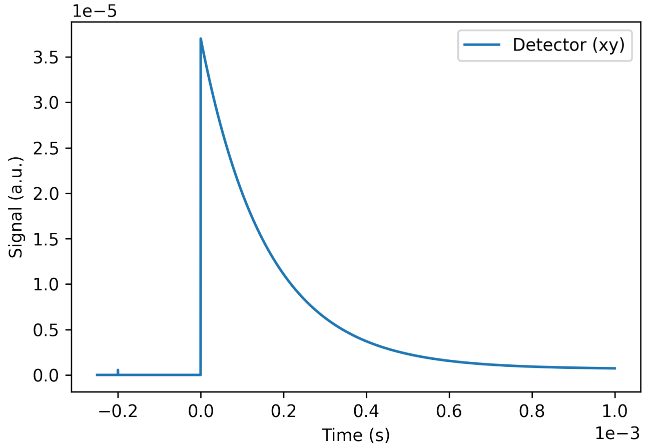

Figure S23: Simulated detector signal for a system with a rotational diffusion time of 100 μs and a delay of 50 μs between the on-switching pulses of STARSS with long-lived states method 3.

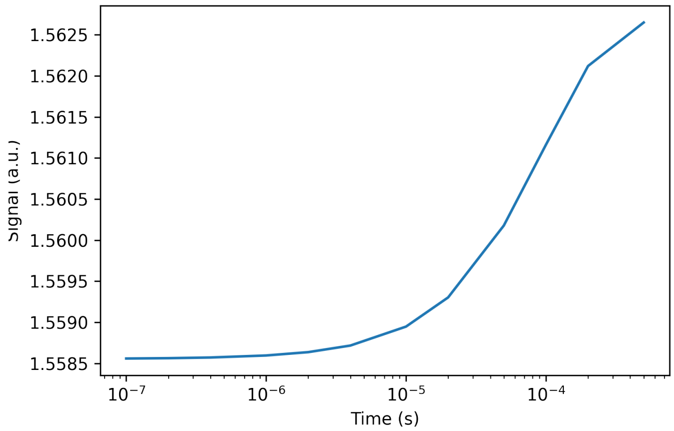

Figure S24: Integrated fluorescence signal computed repeating the simulation of STARSS with long-lived states method 3 as a function of the 405 nm pulse pair delay, which is reported as time axis in the plot. The rotational diffusion time is 100 μs. The normalization of the signal is done by dividing the total counts obtained with a pulse scheme with two 405 nm pulses by the total counts of a pulse scheme with just one 405 nm pulse, i.e. where the first pulse is not shined on the sample.

S10 Saturation curve of ON-switching photo-physics of rsEGFP2

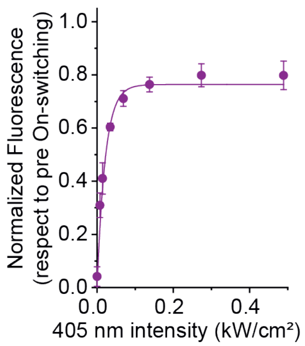

Figure S25: ON-switching saturation curve for rsEGFP2. A sample of U2OS cells endogenously expressing vimentin-rsEGFP2 was measured in a widefield microscope using 1 ms of 405 nm light for ON-switching for each power density condition. A pulse of 1 ms of 488 nm light is used as readout. The fluorescence signal is normalized using the the first cycle of OFF-switching performed with 1 ms of 488 nm light, when the rsFP is fully in the ON-state because of thermal relaxation. Details on the setup and on the cell line are available from Masullo et al. [19].

S11 Structural predictions for rigid fusion of rsEGFP2

We used the crystal structure of rsEGFP2 [20] (5DTX, RCSB Protein Data Bank (PDB, https://www.rcsb.org/)) as reference for the design of rigidly designed STARSS probes. The fluorescent protein beta barrel is flanked by a N-terminal helix and a flexible C-terminus. Since structural information about the trans-membrane domains of the mitochondrial outer membrane proteins OMP25 and was not available in the RCSB PDB, we used structural predictions from the RaptorX web server [21] and the UniProt Knowledgebase (UniProtKB).

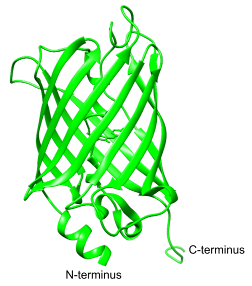

Figure S26: rsEGFP2 crystal structure.

S11.1 OMP25

RaptorX prediction for the OMP25 localization sequence (aa 101-145):

```
Sequence = QVQNGPIGHRGEGDPSGIPIFMVLVPVFALTMVAAWAFMRYRQQL
SSEseq   = LLLLLLLLLLLLLLLLLHHHHHHHHHHHHHHHHHHHHHHHHHHLL
SSEconf  = 987899988998899997658988899999999999999988569
ACCseq   = EEEEEEMEEEEEEEEEEBBBBBBBBBBBBBBBBBBBBBBMMEEEE
ACCconf  = 988777476688664757688887747787788888777555976
```

Topology (from UniProtKB - P57105 (SYJ2B-HUMAN)
Recommended name: Synaptojanin-2-binding protein
Alternative name(s): Mitochondrial outer membrane protein 25

Feature key	Position(s)	Description/Actions	Length
Topological domain	1 – 117	Cytoplasmic	117
Trans-membrane	118 – 138	Helical	21
Topological domain	139 – 145	Mitochondrial intermembrane	7

The linker contains 17 flexible aa from the Omp25 sequence + 2 aa linker (cloning artifact, restriction site) + last 11 aa of rsEGFP2 are a flexible loop. That are 30 aa in a presumably unstructured conformation, which leads to a 10,5 nm flexible linker. Estimated length per aa according to Alberts *et al.* Mol. Biol. Cell 5th Ed., p. 134.[22]

The resulting protein sequence for rsEGFP2-OMP25:

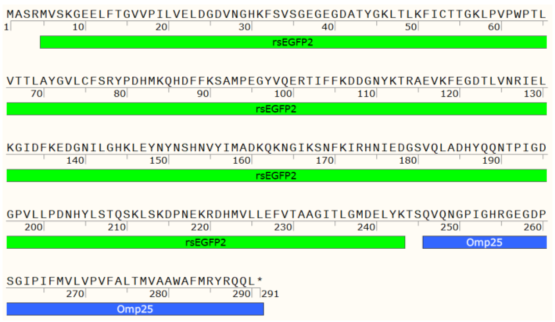

Figure S27: Protein sequence for rsEGFP2-OMP25.

S11.2 TOM20

RaptorX prediction for full length TOM20:
Length = 145

```
Sequence = MVGRNSAIAAGVCGALFIGYCIYFDRKRRSDPNFKNRLRERRKKQKLAKERAGLSKLPDLKDAE
SSEseq   = LLHHHHHHHHHHHHHHHHHHHHHHLLLLLLLHHHHHHHHHHHHHHHHHHHHLLLLLLLLLLLHH
SSEconf  = 9758999999999999999999935666799788999999999999999985899999999999
ACCseq   = EEEEMBBBBBBBBBBBBBBBBBBBBMMMMEMEEBMMEBMEEMEEEEMEEEEEEEEEMMEMEEEE
ACCconf  = 7776455877688787787788685646546768657868757979559887856944557688

AVQKFFLEEIQLGEELLAQGEYEKGVDHLTNAIAVCGQPQQLLQVLQQTLPPPVFQMLLTKLPTISQRIVSAQSLAEDDVE
HHHHHHHHHHHHHHHHHHLLLLHHHHHHHHHHHHLLLHHHHHHHHHHHLLHHHHHHHHHHHLHHHHHHHHHHHHHLLLLLL
999999999997978996999556899999877885995799999999689999999998665556888987555688899
MBEEBBBEMMEMBMMBMEEMEMEEBBMBBBMBBMBBEEMEEBBEBBMEEMMEEBBEMBBEEBEEMMEEMMEEEEEEEEEME
455757474486544647747547895798499455653757646857644886675854547665885586644678849
```

explanations:
(a) 3-class secondary structure (SS3) prediction, L:loop, H: helix
(b) 8-class secondary structure (SS8)prediction,
(c) 3-state solvent accessibility (ACC) prediction, and
(d) disorder (DISO)prediction, respectively.

The part that we used for the rigidly fused Tom20-rsEGFP2 contains only the localization sequence (aa 1-29):

```
Sequence =  MVGRNSAIAAGVCGALFIGYCIYFDRKRR
SSEseq   =  LLHHHHHHHHHHHHHHHHHHHHHHLLLLL
SSEconf  =  97589999999999999999999356667
ACCseq   =  EEEEMBBBBBBBBBBBBBBBBBBBBMMMM
ACCconf  =  77764558776887877877886856465
```

Topology (from UniProtKB - Q15388 (TOM20-HUMAN))

Feature key	Position(s)	Description/Actions	Length
Topological domain	1 – 6	Mitochondrial inter-membrane	6
Trans-membrane	7 – 24	Helical	18
Topological domain	25 – 145	Cytoplasmic	121

We kept the first cytoplasmic amino acids (DRKRR) because these juxtamembrane motif of arginines (R) is known to stabilize the membrane localization of the protein. We then added the rigid helix forming linker (EAAAKA) into the N-terminal helix of (dN)rsEGFP (first 6 aa removed) according to Nakai et al. Microscopy 2019.
Hence, the estimated linker length would be 5x 0.35 nm/aa(loop) + 6x 0.108 nm/aa(helix) = 1,4 nm + 0,65 nm = 2,05 nm whereas only the first 5 aa could be flexible (1,4 nm), but the arginines should have a stabilizing effect as well.

The resulting protein sequence for rigidly fused TOM20-rsEGFP2:

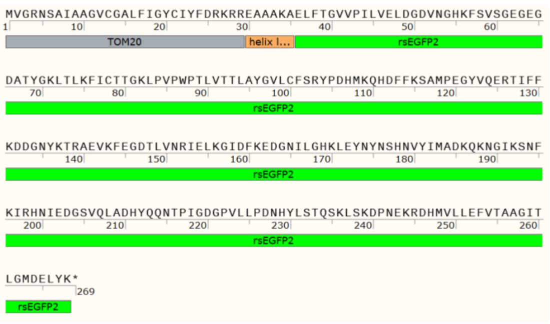

Figure S28: Protein sequence for rigidly fused TOM20-rsEGFP2.

S11.3 H2B

We used the crystal structure PDB: 1ZBB as reference for a nucleosome. Histone

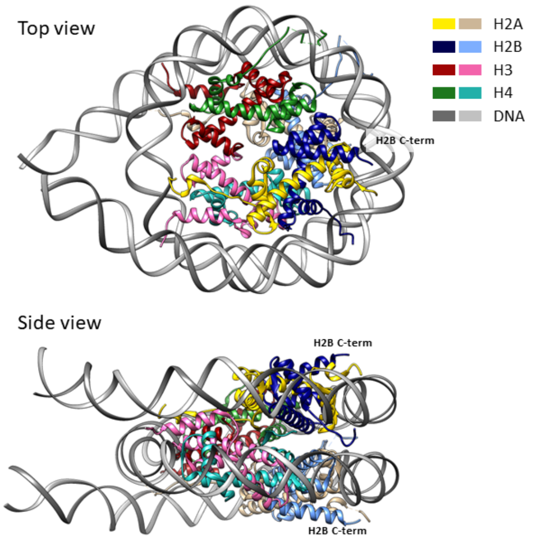

Figure S29: Crystal structure of a nucleosome. The crystal structure model was derived from PDB: 1ZBB. The C-terminal helix of histone H2B are labelled (H2B C-term).

H2B has a C-terminal helix, which was directly fused with rsEGFP2 whereas the first 6 aa were replaced with the rigid helix linker (EAAAKA). Thus the helical structure should be prolonged and the two proteins are fused without flexible linker.

The resulting protein sequence for the rigidly fused H2B-rsEGFP2:

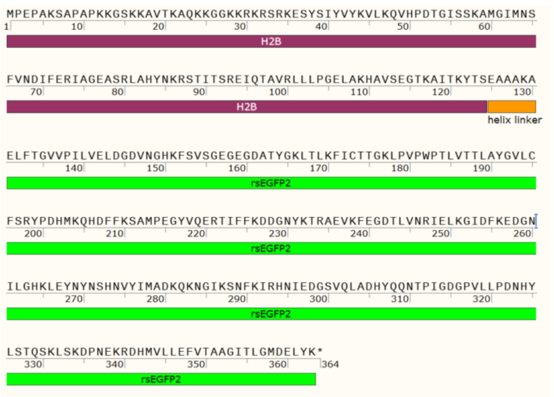

Figure S30: Protein sequence for the rigidly fused H2B-rsEGFP2.

In comparison, in the flexibly fused H2B-rsEGFP2 the C-terminal helix is followed by a linker (DPPVAT); the two prolines break the secondary structure, i.e. they make sure the helix is not prolonged into the N-terminal helix of rsEGFP2. The estimated length of the flexible sequence is less than 2.1 nm because the two prolines should induces a kink.

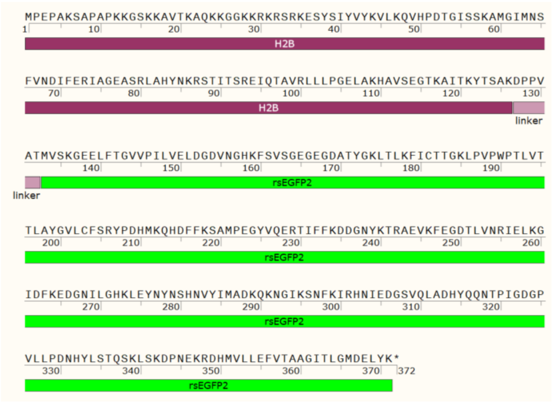

Figure S31: Protein sequence for the flexibly fused H2B-rsEGFP2.

S12 STARSS method 1 compared with steady-state FA for H2B-rsEGFP2 rigid and flexible labels

Standard steady-state fluorescence anisotropy (FA) experiments were performed by illuminating the sample simultaneously with 405 nm at 0.8 kW/cm^2, circularly polarized, and 488 nm at 0.32 kW/cm^2, linearly polarized. The intensity of the 405 nm light was chosen such that the majority of molecules would be in the ON state, i.e. just before saturation of the ON-switching process. The signal was detected in the p and s aligned detection channels. For the steady-state analysis the fluorescence in the detectors was then simply integrated. The result is plotted in blue and orange in the graph for the rigid and flexible H2B-rsEGFP2 samples displaying no significant differences in anisotropy values.

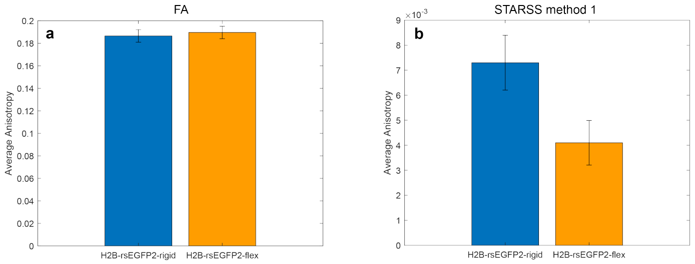

Figure S32: Steady-state anisotropy values (a) and STARSS method 1 average anisotropy values (b) for rigid and flexible H2B-rsEGFP2 label in U2OS live cells. Experimental conditions where chosen in order to achieve a photon counts of approximately 2^7, on top of background, for both techniques. Steady-state ansiotropy (FA) was averaged over n=5 cells, while STARSS method 1 average anisotorpy was integrated over n=3 cells. Errorbar represent confidence intervals (CI 95%). This is an extended dataset of Figure 3c of the main manuscript.

S13 HIV-1 virus-like particle maturation assessment

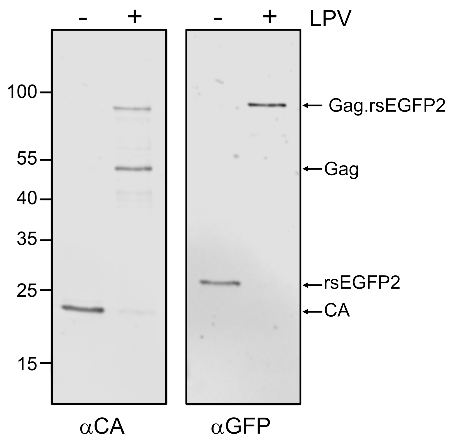

Figure S33: Immunoblot characterization of virus-like particles. Particles were prepared by ultracentrifugation from the tissue culture supernatant of HEK293T cells transfected with an equimolar mixture of plasmids pCHIV and pCHIVirsEGFP2 as described in Materials and Methods. Transfected cells were grown in the absence (-) or presence (+) of 2 µM LPV. Proteins in particle lysates were separated by SDS-PAGE (15%; acrylamide:bis-acrylamide 200:1), transferred to a nitrocellulose membrane by semi-dry blotting. Particle associated proteins were detected by quantitative immunoblot (LI-Cor Biosciences) using polyclonal antisera raised against recombinant HIV-1 CA (sheep; in house) or GFP (rabbit; in house) and cognate secondary antibodies. Bound antibodies were detected using a LI-COR CLx infrared scanner. Positions of molecular mass standards (in kDa) are indicated at the left; positions of immature Gag polyproteins, as well as of the mature CA and rsEGFP2 subunits are marked by arrows.

S14 STARSS method 1 size of HIV-1 virus-like particle

Exponential fitting of the STARSS decay is reported in Table S5 and Figure S34. The fitting procedure is equivalent to what reported in Chapter S4 for the analysis of anisotropy decays recorded for silica beads coated with rsEGFP2.

Sample	τ_R (μs)	r_0	r_∞	d (nm)
Immature	133 (18)	0.071 (0.005)	0.016 (0.002)	100.8 (4.5)
Mature	118 (34)	0.013 (0.002)	0.0045 (0.0009)	97.0 (9.3)

Table S5: Fitted parameters and standard errors between brackets (expressed as square root of the estimated variance computed from the Jacobian of the residuals of the fit). The model function is reported in eq. S5 of Chapter S1. The calculations of the sizes was performed with eq. S3, using 1.0049 cP as viscosity of aqueous environment at 20°C.

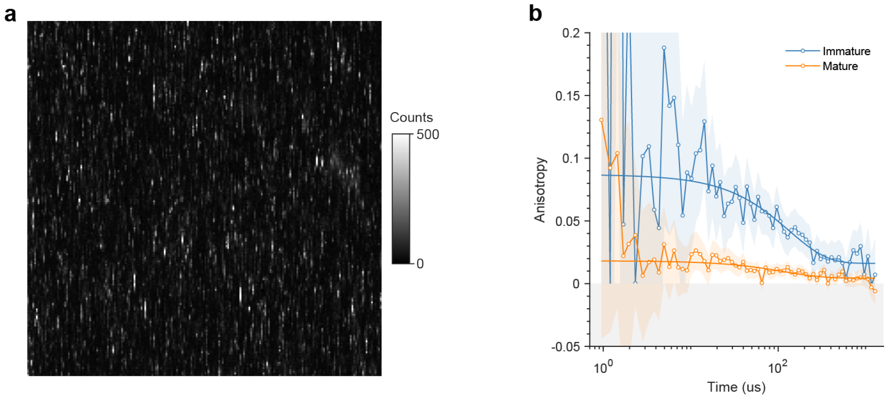

Figure S34: Fit of the STARSS method 1 decay recorded in HIV-1 virus-like particles suspended in liquid solution. (a) Example of counts from the detectors ($I_\parallel + I_\perp$) showed in the spatial domain, every pixel represents a STARSS pulse scheme. Bright spots are virus-like particles temporarily staying inside the confocal spot. Small streaks of high counts appear when freely diffusing virus-like particles are moving by chance along the direction of the fast scanning axis, i.e. vertical axis. (b) Fit of fluorescence anisotropy decays for immature and mature particles.

S15 RESOLFT imaging size of HIV-1 virus-like particles

Super-resolution RESOLFT imaging with a custom MoNaLISA system [19] was performed on HIV-1-rsEGFP2 mature and immature particles anchored on a coverslip using poly-L-lysine. Examples of confocal and RESOLFT images can be found in Figures S35 and S36. Sample preparation was done as described in the Methods. The images are acquired with a sequential illumination of 405 nm light at 0.3 kW/cm^2 for 0.5 ms for on-switching, 488 nm light at 1 kW/cm^2 for 1.5 ms for off-switching, and finally the sub-diffracted areas are read-out with 488 nm light at 1 kW/cm^2 for 1 ms. In the MoNaLISA set-up the three steps of illumination of the RESOLFT acquisition scheme are carried out by a combination of extended light patterns: orthogonally overlapped line patterns with 312.5 nm periodicity for the off-switching and a 2D multi-spots array with a 625 nm period for the on-switching and read-out. The unit area defined by the pattern is scanned with a step of 30 nm for a global frame time of 2.4 sec.

Single particle patches 11 pixels wide (0.330 $\times$ 0.330 μm^2) were extracted from the images. The patches were then fitted using a two-dimensional Lorentzian model function, specified as

$$L_{2D}(x, y) = A\ \frac{1}{\pi} \frac{\Gamma/2}{(x - x_0)^2 + (y - y_0)^2 + (\Gamma/2)^2} + B, \tag{S36}$$

where A is the amplitude, (x_0, y_0) is the center position, Γ is the full-width-half-maximum (FWHM) of the Lorentzian function, and B is the local background. Each particle was fitted using the optimization routine `lsqnonlin` of MATLAB (The MathWorks, Inc.). A collection of the fitted particle patches can be found in Figures S37 and S38.

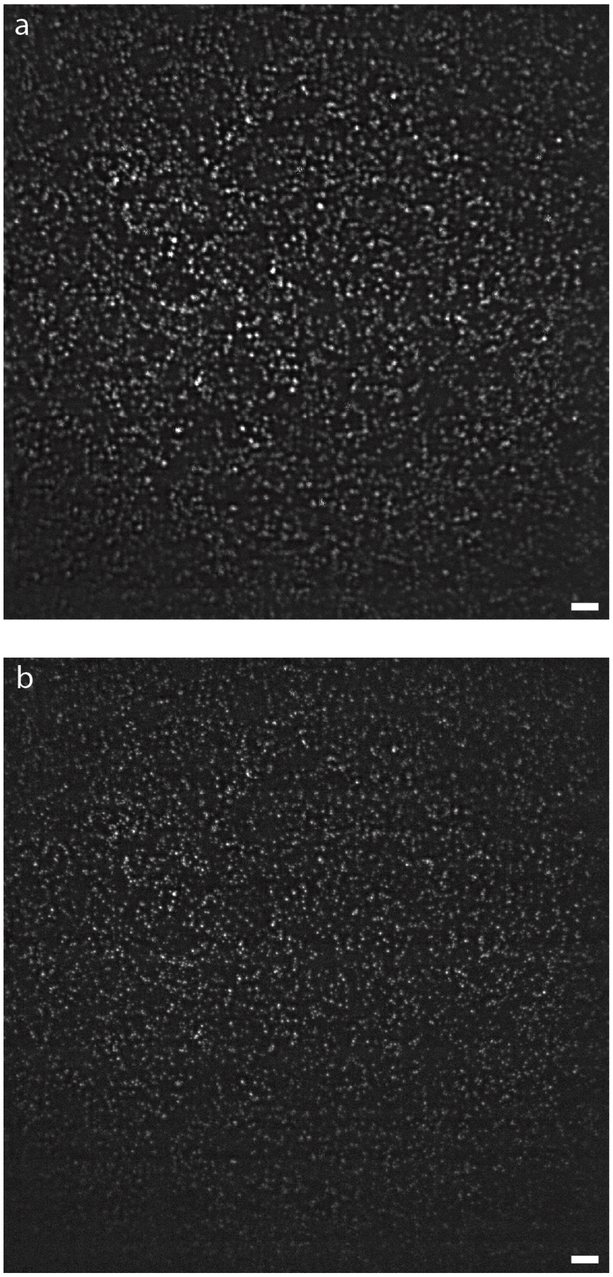

Figure S35: Examples of 40×40 μm^2 confocal (a) and RESOLFT (b) images if mature HIV-1-rsEGFP2 particles. Scale bars are 2 μm.

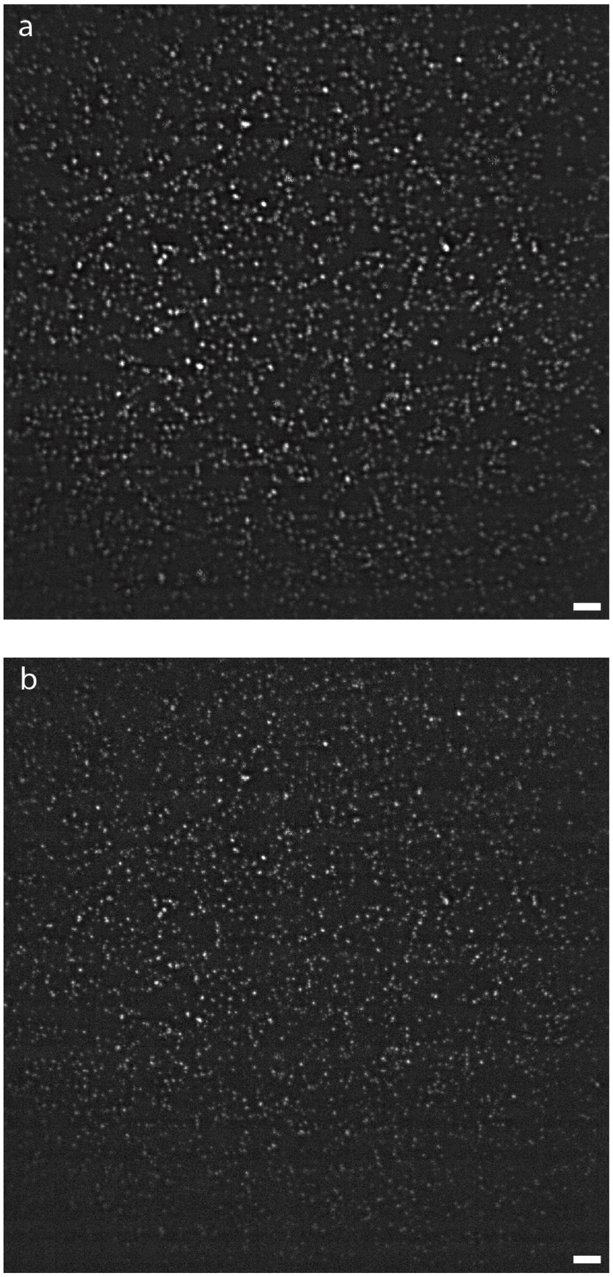

Figure S36: Examples of 40×40 μm^2 confocal (a) and RESOLFT (b) images if immature HIV-1-rsEGFP2 particles. Scale bars are 2 μm.

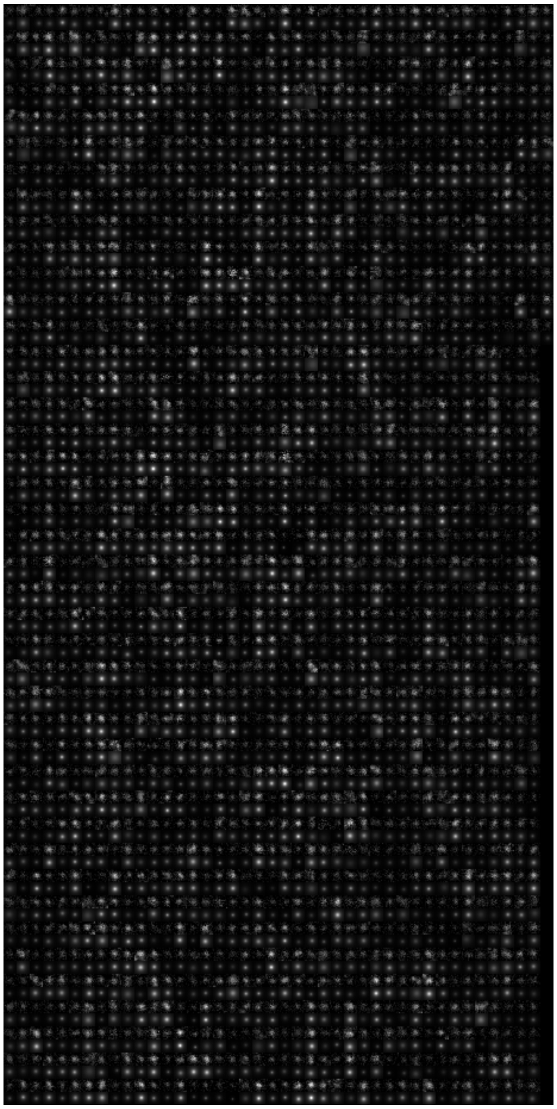

Figure S37: Fitting of single particles extracted from RESOLFT images of HIV-1-rsEGFP2 mature virions. The rows of the image alternately show experimental data and fitted model, i.e. first row is experimental data, second row are fitted model functions, etc. Patch size is $0.330 \times 0.330\ \mu m^2$.

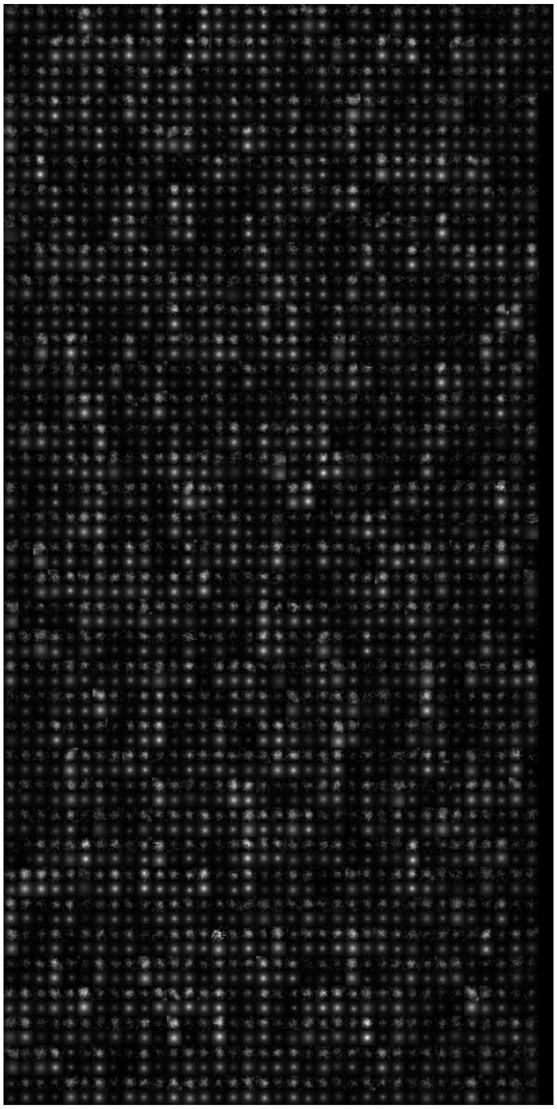

Figure S38: Fitting of single particles extracted from RESOLFT images of HIV-1-rsEGFP2 immature virions. The rows of the image alternately show experimental data and fitted model, i.e. first row is experimental data, second row are fitted model functions, etc. Patch size is $0.330 \times 0.330\ \mu m^2$.

S16 STARSS method 1 fit of Arc-rsEGFP2-IM-fusion data

Sample	τ_R (μs)	r_0	r_∞	d (nm)
Clusters	22.2 (6.1)	0.014 (0.034)	0.0023 (0.0004)	55.5 (5.1)
Cytosol	8.3 (3.2)	0.018 (0.008)	0.0025 (0.0003)	40.0 (5.1)

Table S6: Fitting of Arc-rsEGFP2-IM-fusion data reported in Figure 4h of the main manuscript. Parameters and standard errors between brackets (expressed as square root of the estimated variance from the Jacobian of the residuals of the fit, note that in the main manuscript errors are reported as confidence intervals α=0.05). The model function is reported in eq. S5 of Chapter S1. The calculations of the sizes was performed with eq. S3, using 1.0049 cP as viscosity of aqueous environment at 20°C. Power densities and pulse scheme are reported in Table S7 of Chapter S19.

S17 Photo-bleaching in STARSS experiments

Photo-bleaching in rsFPs due to oxidative chemistry of the chromophore is mainly related to the population of the triplet state from inter-system crossing [23]. The photo-bleaching channel was introduced in the 8-state model by adding a ninth state connected to the anionic excited states, with a rate calculated with a bleaching quantum yield. The ninth state effectively acts as a sink for the bleached population coming from the excitation of the cis-anionic and trans-anionic species. Bleaching coming from the neutral species is neglected in this minimal bleaching model, because the photo-switching by 405 nm illumination is much more efficient and on average less energy is deposited by this wavelength.

For rsEGFP2 typically hundreds-thousands of photo-switching cycles are possible before photo-bleaching. Given the quantum yield of cis-to-trans conformational change of about 10^{-2}, a rough estimate of the effective bleaching quantum yield should be around 10^{-4}-10^{-5}. Using the simulation tool developed as part of this work, a few conditions of bleaching quantum yield were tested and the results are provided in Figures S39 and S40. The simulation uses a rotational diffusion time constant of 100 μs and the same pulse scheme and power densities reported in SI Chapter S2.

In this simplified model of photo-bleaching, the main effect of adding a bleaching channel is to have a faster effective off-switching. For example in Method 1, increased photobleaching induces faster fluorescence decay in both channels, but the circularly polarized 488 light photobleaches fluorophores isotropically, so the anisotropy measurement is not biased. Therefore anisotropies from method 1 are roughly independent of the off-switching kinetics (Figure S39e), thus the addition of bleaching with typical quantum yields does not compromise or affect substantially the ability to retrieve the rotational diffusion time. Conversely, in method 2 the anisotropy values are a result of the competition of rotational diffusion and off-switching. The addition of bleaching makes the new effective off-switching faster, this changes the anisotropy decays (Figure S40e). However, for quantum yields of bleaching up to 10^{-4} a minimal effect is predicted.

In method 3 we expect a minimal effect of photo-bleaching from the on-state since the readout counts are always normalized in successive cycles. Generally, the bleaching induced by 405 nm excitation can be more damaging due to the more energetic wavelength and high power densities. Nevertheless, even if high power densities are involved, the total energy dose deposited is quite low due to the short pulse duration. Indeed, only roughly 50-60 % of the fluorophores are switched into the ON state in 50 ns. With this energy dose, comparable to values used in RESOLFT nanoscopy, thousands of photo-switching cycle can be achieved. As in STARSS method 3 only a handful of photo-switching cycles are required to complete the full delay scan experiments, the fatigue is not severely affected.

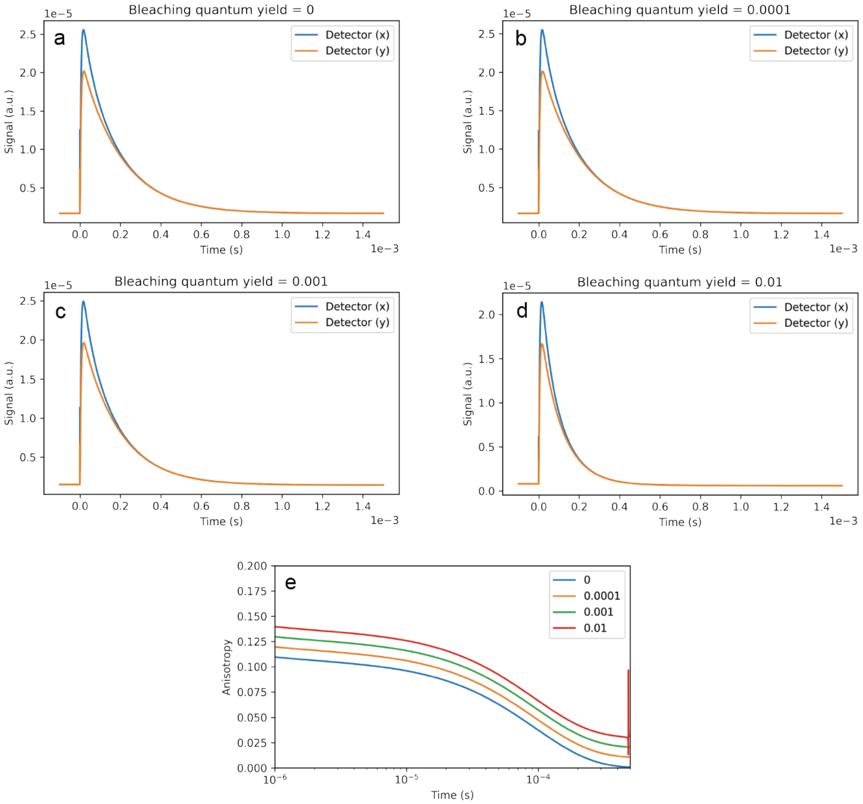

Figure S39: Effect of photo-bleaching in STARSS method 1 experiments. (a-d) Simulation of signals recorded in the virtual detectors at four different values of photo-bleaching quantum yield. (e) Anisotropy curves were obtained from the signals of (a)-(d). The curves are shifted by 0.01 anisotropy units on the vertical axis from each other for visual convenience, i.e. in reality they are superimposed.

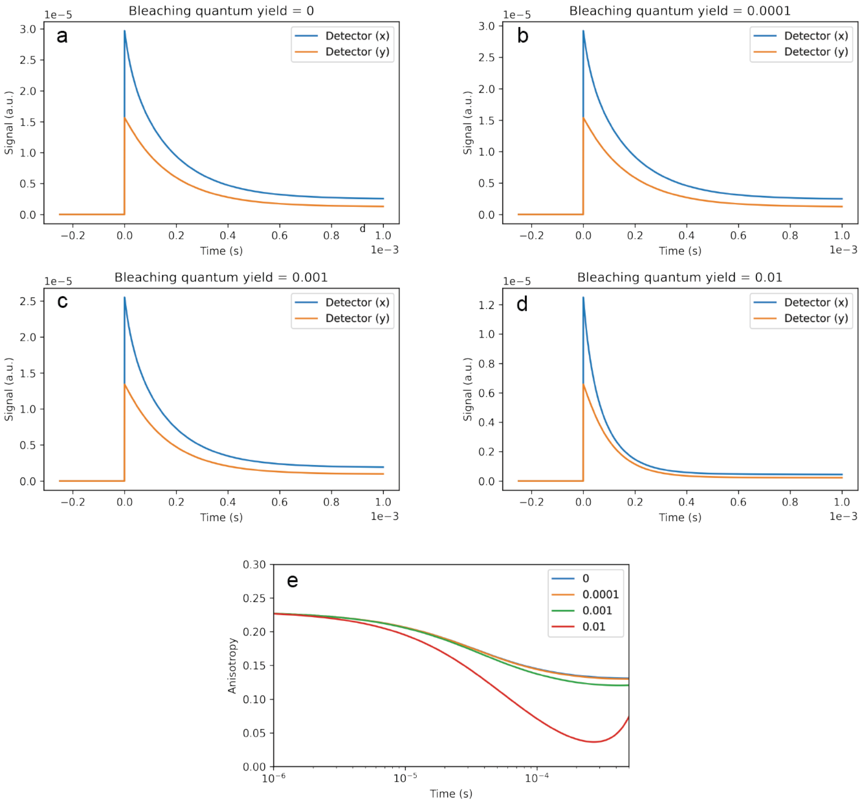

Figure S40: Effect of photo-bleaching in STARSS method 2 experiments. (a-d) Simulation of signals recorded in the virtual detectors at four different values of bleaching quantum yield. (e) Anisotropy curves were obtained from the signals of (a)-(d).

S18 Sensitivity of STARSS for distinguishing complexes of similar sizes

The ability of distinguishing between a complex with size X from a complex with size X + ΔX is related to the signal to noise of the collected signals. Thus, this capability depends on properties of the signal such as background level, total counts, and shape of the curve. To investigate the sensitivity of STARSS curves by examples, we generated signals for beads of diameters 100 and 110 nm with method 1 and method 2 (Figure S41). From this example it can be extrapolated that the max amount of counts necessary to tell apart beads with diameters differing by 10%, given the experimental conditions used in the simulations, should be roughly 10^5. As a practical criteria to verify if the curves are statistically different, they should be separated more than the confidence interval. Experimentally, in the live cell applications reported in the main manuscript, $5 \cdot 10^4$ to 10^6 counts are easily achievable with state of the art rsFPs in standard experimental conditions. Total photon counts can be increased though averaging, by extending the field of view of the measurements or by performing multiple pulse scheme per pixel, at the cost of longer experiment time.

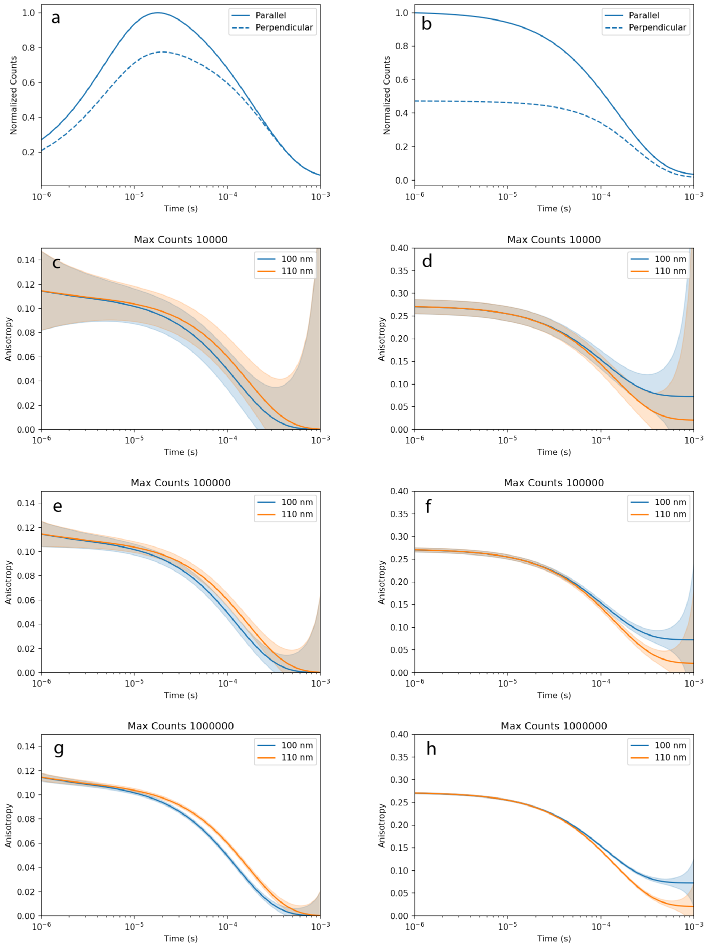

Figure S41: Simulations of STARSS method 1 (left panels) and STARSS method 2 (right panels) experiments of two systems with beads of diameters 100 nm and 110 nm in water at room temperature, using the experiments as defined previously in the chapter. (a,b) Parallel and perpendicular signals simulated in the virtual detectors for a sample with 100 nm beads and rsEGFP2. (c-h) Fluorescence anisotropy curves and the confidence interval at 95% propagated from the photon noise according to the equations in SI Chapter S4, assuming as maximum counts in the parallel channel 10^4, 10^5, and 10^6.

S19 STARSS with long-lived states setup

STARSS with long-lived states experiments were carried out using a custom-built setup as illustrated in Extended Data Fig. 4, adapted from a RESOLFT system originally presented in [24]. Three continuous-wave lasers, one at 405 nm and two at 488 nm (Cobolt AB 06-MLD, Solna, Sweden), were chosen because of the fast modulation in time with sub microsecond accuracy (rise and fall time is less than 10 nanoseconds).

The on-switching laser (405) is expanded using a 4f telescope and then the polarization is cleaned using a Glan-Thomson polarising prism and finely controlled with achromatic $\lambda/2$ and $\lambda/4$ mounted on remotely controlled rotating stages. With this configuration it is possible to calibrate several polarization states of the 405 nm beam, e.g. vertical (V), horizontal (H), and circular (C), and it is possible to reliably inter-convert among them in few seconds.

Two off-switching lasers are installed in the setup. In the pulse schemes where just one is used, the 488-1 is selected. Both of the 488 beams have fine tuning of the polarization with achromatic $\lambda/2$ and $\lambda/4$. A removable spiral 0-2π phase plate is present on the 488-1 beam line, which is used for the generation of a donut point-spread-function only in STARSS/RESOLFT modality 2 super-resolution experiments. Both off-switching laser beams, 488-1 and 488-2, are combined using a 50:50 beam splitter, and then they are further combined with the on-swiching light using a dichroic mirror. A relay 4f 1:1 telescope is used to conjugate the plane of the phase plate with the galvo scanner position. The combined beams are X-Y scanned and sent to the Leica microscope stand, where the sample is holded and scanned in the Z direction using a piezo stage.

The emission light was de-scanned and decoupled using a dichroic mirror and filtered using a band pass, then it is analized using a couple of cross polarized single photon avalanche detectors. The detection light is filtered using a common confocal pinhole, equivalent to about one Airy units. A single pinhole is used to improve the balance of the detectors, i.e. rejecting exactly the same amount of out-of-focus light.

S19.1 Control of the setup using an FPGA

The setup is controlled using a custom modified LabVIEW FPGA software presented in [24]. The FPGA guarantees high time precision for the control of outputs and data acquisition. Briefly, the PCIe-7852R card from National Instruments, which is equiped with a Virtex-5 FPGA chip, is programmed to control all digital/analog input/outputs, and it performs photon counting and time binning of the counts.

The main loop of the FPGA chip is running at 100 MHz. Three digital outputs allow the fast modulation of the laser beams. The system is able to produce down to 50 ns pulses of laser light that have a clean time profile. This performance is achieved exploiting the in-built fast digital modulation provided by the Cobolt lasers. The pulse scheme is controlled with TTL signals with up to 10 programmable time windows. The time binning of the photons is

programmed with a separate synchronized time window, that allow an arbitrary number of bins. The counting is performed inside the FPGA with a second loop running at 200 MHz. Each bin must have a time duration that is a multiple of the period of the main loop of the FPGA, i.e. 10 ns. The galvo scanner and the piezo stage are controlled with three analog output signals. These signals are controlled with a slower repetition rate (750 KHz, limited by the card). The slower rate of analog outputs is not an issue because they are changed only once at the beginning of each pulse scheme.

The smart-scanning system implemented in [24] is fully functional also in the STARSS system. A decision time window can be set, and then the photon counts of both detectors are used to decide if the full STARSS pulse scheme is performed. If the thresholds of photon counts are not met, the system can skip directly to the next pixel. The decision time is usually set to tens of microseconds while a complete STARSS pulse scheme lasts few milliseconds.

Table S7: Experimental conditions for STARSS experiments, where τ_{405} is the duration of each pulse of on-switching light, and I_{405} and I_{488} are the power densities of the corresponding beams. Power densities were measured replacing the objective with an iris of the size of the back-aperture pupil, and considering the power focused on a circular target with diameter equal to the full-width-half-maximum (FWHM) of the confocal point spread function (PSF). FWHM values were 205 nm for 405 nm, and 220 nm for 488 nm. PSFs were mesasured using the scattering signal from a gold beads sample.

Experiment	τ_{405} (s)	I_{405} (kW/cm^2)	I_{488} (kW/cm^2)	Pixel size (nm)	Repetitions per pixel	FOV (pixel)	Number of FOV (or cells)
**STARSS method 1**							
rsFPs on coverslip (Figure 2)	$250 \cdot 10^{-9}$	235	55.1	100	1	200x200	3
Silica beads library (Figure 2)	$250 \cdot 10^{-9}$	177	17.6	80	5	200x200	1
Mithocondria rsEGFP2 (Figure 3a)	$250 \cdot 10^{-9}$	115	4.89	80	1	200x200	9 (TOM20) - 7 (OMP25)
Histone rsEGFP2 live-fixed (Figure 3c)	$250 \cdot 10^{-9}$	115	7.34	80	1	200x200	8
Histone rsEGFP2 (Figure 3d-f)	$250 \cdot 10^{-9}$	109	21.5	80	1	256x256	8 - 18 (mitotic)
HIV-1 virions rsEFGP2 (Figure 4)	$250 \cdot 10^{-9}$	142	26.3	80	1	200x200	5 (imm.) - 4 (mat.)
HIV-1 virions rsEFGP2 single-particle (Figure 4)	$250 \cdot 10^{-9}$	142	6.58	40	10	9x9	36 (imm.) - 57 (mat.)
Arc rsEGFP2 (Figure 4)	$250 \cdot 10^{-9}$	109	21.5	80	1	256x256	5 (ΔNTD) - 12 (IM-fusion)
**STARSS method 2**							
Silica beads library (Figure 2)	$250 \cdot 10^{-9}$	115	19.6	80	1	200x200	1
HIV-1 virions rsEFGP2 (Figure 4)	$250 \cdot 10^{-9}$	115	19.6	80	1	200x200	1
HIV-1 virions rsEFGP2 single-particle (Figure 4)	$2 \cdot 10^{-6}$	328	52	25	1	11x11	136 (imm.) - 179 (mat.)
**STARSS method 2 (super-res)**							
HIV-1 virions rsEFGP2 (Figure 4)	$2 \cdot 10^{-6}$	328	35 (donut)<br>52 (readout)	25	1	11x11	122 (imm.) - 185 (mat.)
**STARSS method 3**							
Silica beads library (Figure 2)	$50 \cdot 10^{-9}$	7120	14	80	3	20x20	12 delay times

S20 Notes on data analysis and anisotropy variance estimation

Anisotropy data obtained with STARSS rsFP-based methods is affected by background fluorescence signal generated by simultaneous on- and off-switching, i.e., crosstalk. It is especially present when, in order to reset the system, a long pulse of 488 nm light is applied to the sample until the system reaches steady state. In this state, most of the molecules are off-switched. Nevertheless, there is still a small on-switching probability that keeps a fraction of molecules in the on-state. The background is computed by averaging multiple bins in the steady state regime to reduce the variance. We then assume that the calculated background is a deterministic variable for simplicity.

S20.1 Estimation of anisotropy variance

The anisotropy value is computed from the intensity values at the detectors, which are single photon counters.

We define the intensities at each detector, $I_{\parallel}$ and $I_{\perp}$, as Poisson distributed random variables, since they are mainly corrupted by photon noise.

$$\begin{aligned} I_{\parallel} &\sim \text{Poisson}(\lambda_{I_{\parallel}}) \\ I_{\perp} &\sim \text{Poisson}(\lambda_{I_{\perp}}) \end{aligned} \tag{S37}$$

where $\lambda_{I_{\parallel}} = E(I_{\parallel})$ and $\lambda_{I_{\perp}} = E(I_{\perp})$ are the expected values of the distributions. The variance (Var) is directly defined by the Poison distribution and the covariance (Cov) is derived from the statistics of the beam splitter

$$\begin{aligned} Var(I_{\parallel}) &= E(I_{\parallel}) \\ Var(I_{\perp}) &= E(I_{\perp}) \\ Cov(I_{\parallel}, I_{\perp}) &= -\frac{E(I_{\parallel})E(I_{\perp})}{E(I_{\parallel}) + E(I_{\perp})}. \end{aligned} \tag{S38}$$

Subtracting the background levels $b_{\parallel}$ and $b_{\perp}$ of the two detector leads to two new random variables

$$\begin{aligned} I'_{\parallel} &= I_{\parallel} - b_{\parallel} \\ I'_{\perp} &= I_{\perp} - b_{\perp}. \end{aligned} \tag{S39}$$

The fluorescence anisotropy is computed as

$$r = \frac{I'_{\parallel} - I'_{\perp}}{I'_{\parallel} + 2I'_{\perp}} = \frac{N}{D}, \tag{S40}$$

where N and D are defined as the numerator and denominator respectively.

From the properties in eq. S38 and the definition in eq. S39 we estimate the variance of the anisotropy as

$$\widehat{Var}(r) = \frac{Var(N)}{E(D)^2} - \frac{2E(N)Cov(N,D)}{E(D)^3} + \frac{Var(D)}{E(D)^4}, \tag{S41}$$

where

$$\begin{aligned}
E(N) &= E(I_{\|}) - E(I_{\perp}) + b_{\|} - b_{\perp} \\
E(D) &= E(I_{\|}) + E(I_{\perp}) + b_{\|} + 2b_{\perp} \\
Var(N) &= E(I_{\|}) + E(I_{\perp}) - 2Cov(I_{\|}, I_{\perp}) \\
Var(N) &= E(I_{\|}) + 4E(I_{\perp}) + 4Cov(I_{\|}, I_{\perp}) \\
Cov(N, D) &= E(I_{\|}) - 2E(I_{\perp}) + Cov(I_{\|}, I_{\perp}).
\end{aligned} \tag{S42}$$

In the main manuscript, all the anisotropy plots have confidence intervals ($\alpha = 0.05$) estimated in the Gaussian approximation as

$$r_{\pm}^{\alpha=0.05} = r \pm 1.96\sqrt{\widehat{Var}(r)}. \tag{S43}$$

S20.2 Log-scale time binning of counts

Signals collected using STARSS with rsFPs were plotted using a logarithmic time scale. The signal was binned in a non-linear logarithmic fashion in order to show the details of the signal at both fast and slow timescales.

The signal was processed by removing the background and a certain number of raw time bins were summed. The time associated to the final bin was the average of the raw bin times. The number of summed bins was varied logarithmic-ally with the time axis.

We define the signal from the i-th detector as I_i, with associated time t_i. After the non-linear binning of the raw data we obtain a new series of data points $\tilde{I}_j$ with new time coordinates $\tilde{t}_j$. The new data points are computed as

$$\begin{aligned}
\tilde{I}_j &= \sum_{i \in U_j} I_i \\
\tilde{t}_j &= \frac{\sum_{i \in U_j} t_i}{N_j},
\end{aligned} \tag{S44}$$

where N_j is the number of bins, and U_j is the ensemble of bin indexes. Typical length of raw time bins is 0.5-1.0 μs, while after the log-spacing time bins ranges from 0.5-1.0 μs to approximately 50-100 μs.

S20.3 Average and time resolved anisotropy data

In STARSS method 1 (Fig.2a) two time-resolved curves are recorded respectively from channel 1 and 2 (Fig.2b), which are sensitive to p and s polarized light. The background fluorescence is then subtracted from the curves, the background value is encoded in each pulse scheme after completing the switching off (first blue pulse in Fig.2a). For the average anisotropy data the total counts in each channel for the temporal bin 1-100 μs are then summed up. Each temporal bin has more or less photons due on the ON and OFF switching properties; therefore not all temporal bins will contribute equally on the final result but each will have more or less weight. The temporal bins around 10-100 μs typically show the highest photon counts and drive the final average value.

For the time resolved curves, the photon counts detected in each temporal bin in the p and s channel are averaged firsty for each X and Y pixels and when the number of pixels of a single FOV are not enough also within different FOV. This is done in order to achieve an anisotropy curve with enough count to be significantly different from the zero values and not dominated by the detector shot noise. For anisotropy value of about 10^{-1} as the one measured in the beads sample we need at least 10^3 photons per temporal bin to build a STARSS time resolved curve. In cells, where the anisotropy values are lower and around 10^{-2} the total photon counts needed is at least $\sim 10^5$. To reach this photon count value within a temporal bin in cells typically 4-10 cells are needed. The shadow curve in each figure is the propagation of the detector shot noise as described in Section S20.1 and eq. S43.

In STARSS method 2 the raw counts in both channels are treated in the very same way. STARSS method 3 instead averaged all the photon counts during the blue light illumination, which are then normalized after background subtraction. The normalization value is the photon count after background subtraction, generated upon blue light illumination after a single ON-switching pulse.

S21 Primer list

	Sequence	Template		Target		Fragment
1	TAC CGC GGG CCC GGG ATC CAC CGG TCG CCA CCA TGG TGA GCA AGG GCG AGG AG	pDOS03	N-cadherin-rsEGFP2	pDOS66	H2B-rsEGFP2	rsEGFP2
2	ATC TAG AGT CGC GGC CGC TTT ACT TGT ACA GCT CGT CCA TGC	pDOS03	N-cadherin-rsEGFP2	pDOS66	H2B-rsEGFP2	rsEGFP2
3	CGG CAT GGA CGA GCT GTA CAA G	pDOS66	H2B-rsEGFP2	pDOS73	H2B-rigid-rsEGFP2	H2B-backbone
4	AAG TAC ACC AGC GAG GCT GCT GCT AAA GCT GAG CTG TTC ACC GGG GTG GTG CCC ATC C	pDOS66	H2B-rsEGFP2	pDOS73	H2B-rigid-rsEGFP2	H2B-backbone
5	ACC CCG GTG AAC AGC TCA GCT TTA GCA GCA GCC TCG CTG GTG TAC TTG GTG ATG GCC	pDOS66	H2B-rsEGFP2	pDOS73	H2B-rigid-rsEGFP2	rigid-rsEGFP2
6	CTT GTA CAG CTC GTC CAT GCC G	pDOS66	H2B-rsEGFP2	pDOS73	H2B-rigid-rsEGFP2	rigid-rsEGFP2
7	TAA TAC GAC TCA CTA TAG GG	pDOS49	mitoLAMA-F98	pDOS79	TOM20-rigid-rsEGFP2	TOM20
8	AAC AGC TCA GCT TTA GCA GCA GCC TCG CGG CGT TTG CGA TCA AAG TAA ATG C	pDOS49	mitoLAMA-F98	pDOS79	TOM20-rigid-rsEGFP2	TOM20
9	GAG GCT GCT GCT AAA GCT GAG CTG TTC ACC	pDOS64	UrsG7	pDOS79	TOM20-rigid-rsEGFP2	backbone
10	TAG AAG GCA CAG TCG AGG	pDOS64	UrsG7	pDOS79	TOM20-rigid-rsEGFP2	backbone

	Sequence	Template		Target		Fragment
11	ATG ACT GGA TCC ATG GTG AGC AAG GGC GAG GAG	IT006	rsEGFP2-Omp25	AAV003	EF1a-rsEGFP2	rsEGFP2
12	AGT CAT GAA TTC TCA CTT GTA CAG CTC GTC CAT GCC G	IT006	rsEGFP2-Omp25	AAV003	EF1a-rsEGFP2	rsEGFP2
13	ATG ACT GGT ACC GGA TTG GCC ACC ATG GAG CTG GAC CAT ATG ACG	IT046	Arc-C-EGFP	pAAV_MD005	Arc-C-rsEGFP2	Arc
14	AGT CAT GGA TCC GCT ACC GCT GCC TTC AGG CTG GGT CCT GTC ACT	IT046	Arc-C-EGFP	pAAV_MD005	Arc-C-rsEGFP2	Arc
15	GCA TGG ACG AGC TGT ACA AGG GCG GCT CCA TCA TGG GCG GCA AGT ACC CA	IT046	Arc-C-EGFP	pAAV_MD018	Arc-IM-fusion-rsEGFP2	Arc-CTD
16	TAT CGA TAA GCT TGA TAT CGT TAT TCA GGC TGG GTC CTG TC	IT046	Arc-C-EGFP	pAAV_MD018	Arc-IM-fusion-rsEGFP2	Arc-CTD
17	GGG CCG ACC GCC TGG AGT CCG GCT CCA TCG GCG GCG TGA GCA AGG GCG AGG	IT006	rsEGFP2-Omp25	pAAV_MD018	Arc-IM-fusion-rsEGFP2	rsEGFP2
18	ACT GGG TAC TTG CCG CCC ATG ATG GAG CCG CCC TTG TAC AGC TCG TCC ATG C	IT006	rsEGFP2-Omp25	pAAV_MD018	Arc-IM-fusion-rsEGFP2	rsEGFP2
19	TTT CAG GTG TCG TGA GGT ACG GAT TGG CCA CCA TGG AG	pAAV_MD014	Arc-C-SNAP	pAAV_MD021	Arc-only	Arc
20	TAT CGA TAA GCT TGA TAT CGT TAT TCA GGC TGG GTC CTG TC	pAAV_MD014	Arc-C-SNAP	pAAV_MD021	Arc-only	Arc

References

[1] J. J. Fisz, Chemical Physics Letters **262**, 495 (1996).

[2] J. J. Fisz, Chemical Physics Letters **262**, 507 (1996).

[3] R. Courant and D. Hilbert, *Methods of Mathematical Physics, Volume 1* (Wiley-VCH, 1991).

[4] M. A. Wieczorek and M. Meschede, Geochemistry, Geophysics, Geosystems **19**, 2574 (2018).

[5] J. R. Lakowicz, *Principles of Fluorescence Spectroscopy* (Kluwer Academic Publishers, 1999).

[6] G. R. Satchler and D. M. Brink, *Angular Momentum* (Oxford University Press, 1994).

[7] J. J. Fisz, M. Buczkowski, and P. Zuchowski, Chemical Physics Letters **412**, 263 (2005).

[8] N. Tkachenko, *Optical Spectroscopy* (Elsevier Science, 2007).

[9] S. P. Laptenok, A. A. Gil, C. R. Hall, A. Lukacs, J. N. Iuliano, G. A. Jones, G. M. Greetham, P. Donaldson, A. Miyawaki, P. J. Tonge, and S. R. Meech, Nature Chemistry **10**, 845 (2018).

[10] J. Woodhouse, G. Nass Kovacs, N. Coquelle, L. M. Uriarte, V. Adam, T. R. Barends, M. Byrdin, E. de la Mora, R. Bruce Doak, M. Feliks, M. Field, F. Fieschi, V. Guillon, S. Jakobs, Y. Joti, P. Macheboeuf, K. Motomura, K. Nass, S. Owada, C. M. Roome, C. Ruckebusch, G. Schirò, R. L. Shoeman, M. Thepaut, T. Togashi, K. Tono, M. Yabashi, M. Cammarata, L. Foucar, D. Bourgeois, M. Sliwa, J. P. Colletier, I. Schlichting, and M. Weik, Nature Communications **11**, 1 (2020).

[11] K. Nienhaus and G. U. Nienhaus, Journal of Physics Condensed Matter **28**, 443001 (2016).

[12] D. Yadav, F. Lacombat, N. Dozova, F. Rappaport, P. Plaza, and A. Espagne, Journal of Physical Chemistry B **119**, 2404 (2015).

[13] M. El Khatib, A. Martins, D. Bourgeois, J. P. Colletier, and V. Adam, Scientific Reports **6**, 1 (2016).

[14] I. Testa, E. D'Este, N. T. Urban, F. Balzarotti, and S. W. Hell, Nano Letters **15**, 103 (2015).

[15] D. Yadav, F. Lacombat, N. Dozova, F. Rappaport, P. Plaza, and A. Espagne, Journal of Physical Chemistry B **119**, 2404 (2015).

[16] M. Chattoraj, B. A. King, G. U. Bublitz, and S. G. Boxer, Proceedings of the National Academy of Sciences **93**, 8362 (1996).

[17] E. Fron, C. Flors, G. Schweitzer, S. Habuchi, H. Mizuno, R. Ando, F. C. De Schryver, A. Miyawaki, and J. Hofkens, Journal of the American Chemical Society **129**, _eprint: https://doi.org/10.1021/ja069365v, 4870 (2007).

[18] Y. Teijeiro-Gonzalez, A. Crnjar, A. J. Beavil, R. L. Beavil, J. Nedbal, A. Le Marois, C. Molteni, and K. Suhling, Biophysical Journal **120**, 254 (2021).

[19] L. A. Masullo, A. Bodén, F. Pennacchietti, G. Coceano, M. Ratz, and I. Testa, Nature Communications **9**, 10.1038/s41467-018-05799-w (2018).

[20] M. El Khatib, A. Martins, D. Bourgeois, J. P. Colletier, and V. Adam, Scientific Reports **6**, 1 (2016).

[21] M. Källberg, H. Wang, S. Wang, J. Peng, Z. Wang, H. Lu, and J. Xu, Nature Protocols **7**, 1511 (2012).

[22] B. Alberts, A. Johnson, P. Walter, J. Lewis, M. Raff, and K. Roberts, *Molecular biology of the cell 5E*, 5th ed. (CRC Press, Boca Raton, FL, Nov. 2007).

[23] M. Byrdin, C. Duan, D. Bourgeois, and K. Brettel, Journal of the American Chemical Society **140**, 2897 (2018).

[24] J. Dreier, M. Castello, G. Coceano, R. Cáceres, J. Plastino, G. Vicidomini, and I. Testa, Nature Communications **10**, 10.1038/s41467-019-08442-4 (2019).
