# Supplementary material for: Extending fluorescence anisotropy to large complexes using reversibly switchable proteins
Source: Nat Biotechnol. 2022 Oct 10;41(4):552–9. doi: 10.1038/s41587-022-01489-7 (PMC10110461; doi:10.1038/s41587-022-01489-7)
Supplement: Supplementary file 2 — Reporting Summary [file 41587_2022_1489_MOESM2_ESM.pdf]

nature portfolio

Corresponding author(s): Ilaria Testa

Last updated by author(s): Aug 22, 2022

Reporting Summary

Nature Portfolio wishes to improve the reproducibility of the work that we publish. This form provides structure for consistency and transparency in reporting. For further information on Nature Portfolio policies, see our Editorial Policies and the Editorial Policy Checklist.

Statistics

For all statistical analyses, confirm that the following items are present in the figure legend, table legend, main text, or Methods section.

n/a	Confirmed	
☒	☐	The exact sample size (*n*) for each experimental group/condition, given as a discrete number and unit of measurement
☐	☒	A statement on whether measurements were taken from distinct samples or whether the same sample was measured repeatedly
☒	☐	The statistical test(s) used AND whether they are one- or two-sided<br>*Only common tests should be described solely by name; describe more complex techniques in the Methods section.*
☒	☐	A description of all covariates tested
☒	☐	A description of any assumptions or corrections, such as tests of normality and adjustment for multiple comparisons
☐	☒	A full description of the statistical parameters including central tendency (e.g. means) or other basic estimates (e.g. regression coefficient) AND variation (e.g. standard deviation) or associated estimates of uncertainty (e.g. confidence intervals)
☒	☐	For null hypothesis testing, the test statistic (e.g. *F*, *t*, *r*) with confidence intervals, effect sizes, degrees of freedom and *P* value noted<br>*Give P values as exact values whenever suitable.*
☒	☐	For Bayesian analysis, information on the choice of priors and Markov chain Monte Carlo settings
☒	☐	For hierarchical and complex designs, identification of the appropriate level for tests and full reporting of outcomes
☒	☐	Estimates of effect sizes (e.g. Cohen's *d*, Pearson's *r*), indicating how they were calculated

Our web collection on statistics for biologists contains articles on many of the points above.

Software and code

Policy information about availability of computer code

Data collection	For the imaging, the control of the hardware are part of our open-source microscope control project developed in Python available at https://github.com/TestaLab, additional LabVIEW code for FPGA control is available upon request.
Data analysis	Code for simulation of STARSS experiments is available at https://github.com/TestaLab, any other script and small piece of code is available upon request.

For manuscripts utilizing custom algorithms or software that are central to the research but not yet described in published literature, software must be made available to editors and reviewers. We strongly encourage code deposition in a community repository (e.g. GitHub). See the Nature Portfolio guidelines for submitting code & software for further information.

Data

Policy information about availability of data

All manuscripts must include a data availability statement. This statement should provide the following information, where applicable:

- Accession codes, unique identifiers, or web links for publicly available datasets
- A description of any restrictions on data availability
- For clinical datasets or third party data, please ensure that the statement adheres to our policy

No restriction on data availability. Minimal datasets are available on Zenodo (10.5281/zenodo.7010471). All code and raw data are available upon request.

Field-specific reporting

Please select the one below that is the best fit for your research. If you are not sure, read the appropriate sections before making your selection.

☒ Life sciences ☐ Behavioural & social sciences ☐ Ecological, evolutionary & environmental sciences

For a reference copy of the document with all sections, see nature.com/documents/nr-reporting-summary-flat.pdf

Life sciences study design

All studies must disclose on these points even when the disclosure is negative.

Sample size	No sample size dependent statistical testing was performed.
Data exclusions	No data were excluded from the analysis.
Replication	The STARSS curve presented were reproducible in different days of recording and for extended times after system alignment. System alignment was stable over time for the full day of recording.
Randomization	No allocation into experimental group were performed
Blinding	No allocation into experimental group were performed

Reporting for specific materials, systems and methods

We require information from authors about some types of materials, experimental systems and methods used in many studies. Here, indicate whether each material, system or method listed is relevant to your study. If you are not sure if a list item applies to your research, read the appropriate section before selecting a response.

Materials & experimental systems

n/a	Involved in the study	
☐	☒	Antibodies
☐	☒	Eukaryotic cell lines
☒	☐	Palaeontology and archaeology
☒	☐	Animals and other organisms
☒	☐	Human research participants
☒	☐	Clinical data
☒	☐	Dual use research of concern

Methods

n/a	Involved in the study	
☒	☐	ChIP-seq
☒	☐	Flow cytometry
☒	☐	MRI-based neuroimaging

Antibodies

Antibodies used	Polyclonal antisera raised against recombinant HIV-1 capsid (CA) (sheep; in house) or GFP (rabbit; in house); secondary antibodies donkey anti-sheep IgG DyLight 680 (#613-744-168; Rockland Immunochemicals, USA), IRdye800CW donkey anti-rabbit IgG (#926-32213; LI-COR Biosciences). Recombinant Anti-GFP antibody [EPR14104] (ab183734) was used to decorate rsEGFP2 coated silica beads.
Validation	The in-house antibodies have tested and used in many publications over the past 20 years. Here are few examples: doi.org/10.1016/j.cell.2021.01.025, doi.org/10.7554/eLife.41800, doi:10.1038/nature13838. We think that a formal validation is not necessary in this case.

Eukaryotic cell lines

Policy information about cell lines

Cell line source(s)	ATCC
Authentication	None of the cell lines used were authenticated.
Mycoplasma contamination	All cell lines were tested negative for mycoplasma contamination.
Commonly misidentified lines (See ICLAC register)	HEK293T cells were validated in 2017 by Eurofins Medigenomics Forsensic GmbH (Ebersberg, Germany).
